# Celecoxib Enhances Oxidative Muscle Fibre Formation and Improves Muscle Functions Through Prokr1 Activation in Mice

**DOI:** 10.1002/jcsm.13704

**Published:** 2025-01-30

**Authors:** Jeong Hwan Park, Jongsoo Mok, Seoah Park, Dooho Kim, Min‐Su Kang, Tae Sub Park, Joonghoon Park

**Affiliations:** ^1^ Department of International Agricultural Technology, Graduate School of International Agricultural Technology Seoul National University Seoul Republic of Korea; ^2^ Institute of Green Bio Science and Technology Seoul National University Seoul Republic of Korea

**Keywords:** celecoxib, exercise performance, muscle mass, oxidative muscle fibre, PROKR1

## Abstract

**Background:**

Muscle diseases are serious challenges to human health. Prokineticin receptor 1 (PROKR1) has emerged as a potential target to improve muscle function through increasing oxidative muscle fibres, but there are no clinically applicable synthetic PROKR1 agonists.

**Methods:**

Drugs with biological properties of prokineticin 2 (PK2) were discovered through connectivity map (CMap) analysis. Their effects on PROKR1 were evaluated using molecular docking, PROKR1 signalling and competitive binding assays. Pregnant dams were fed diets containing varying celecoxib concentrations (0, 500, 1000 and 1500 ppm) from gestation day 5 through weaning. Offspring were given high‐fat diets (HFD) from weaning until 20 weeks old, and body composition, insulin resistance, energy expenditure, exercise performance and histological analysis of muscle tissues were evaluated.

**Results:**

Celecoxib, with a connectivity score of 64.19 to PK2 and a docking score of −9.0 to PROKR1, selectively activated Gs signalling at 4 μM of EC_50_ and increased NR4A2 protein levels by 1.6‐fold (*p* < 0.01) in PROKR1‐overexpressing cells. It competitively inhibited PK2 binding to PROKR1 and reduced cAMP accumulation. In murine and human myotubes, celecoxib increased Prokr1 protein levels by 1.8‐fold (*p* < 0.05), pCreb by 1.5‐fold (*p* < 0.05) and Nr4a2 by 1.3‐fold (*p* < 0.05). It also elevated Myh7 index by 2.2‐fold (*p* < 0.0001), mitochondrial content by 1.6‐fold (*p* < 0.001) and fatty acid oxidation (FAO) activity by 4.1‐fold (*p* < 0.05). Offspring exposed to celecoxib during pre‐ and postnatal muscle development exhibited activated Prokr1 signalling, enhanced oxidative muscle fibre formation and improved muscle phenotype despite HFD. At weaning, both male and female offspring showed dose‐dependent increases in lean mass (> 9.35%, *p* < 0.001) and grip strength (< 18.0%, *p* < 0.01). At 12 weeks old, mice displayed a dose‐dependent decrease in weight loss (> 13.3%, *p* < 0.05), increased lean mass (> 16.2%, *p* < 0.05), improved insulin resistance (> 70.4%, *p* < 0.0001), energy expenditure (> 173%, *p* < 0.0001) and grip strength (> 23.5%, *p* < 0.001). Celecoxib also increased Myh7‐positive muscle fibre composition (> 10.8%, *p* < 0.05) and mitochondrial mass (> 32.8%, *p* < 0.05) in the gastrocnemius and soleus muscles, accompanied by significant Prokr1 signalling activation. These effects persisted in both male and female mice at 20 weeks old.

**Conclusions:**

Celecoxib shows promise as a PROKR1 agonist and clinically applicable exercise mimetic for the treatment of muscular disorders.

## 1 Introduction

Muscle development is an intricate and tightly regulated process that plays a vital role in the growth and function of an organism [1], and muscular disorders encompass a wide range of conditions that affect the muscles. Thus, muscle diseases such as sarcopenia and cachexia pose significant challenges to human health [2, 3]. In addition, the impaired glucose and lipid metabolism observed in obesity and Type 2 diabetes contributes to an imbalance in muscle fibre composition and muscle wasting [4, 5]. These muscular disorders not only affect the quality of life of individuals but also impose a significant burden on healthcare systems worldwide. Therefore, there is an urgent need for effective therapeutic strategies to prevent or treat these conditions.

Exercise has long been recognized as a potent intervention for improving muscle function and overall health [6, 7, 8]. However, not everyone is able to engage in regular exercise due to various limitations, such as age, disability or medical conditions. This has prompted research groups to investigate exercise mimetics [9, 10]. Although certain exercise mimetics have shown promising results in preclinical and early clinical trials, the identification of therapeutic targets that can mimic the various effects of exercise remains a significant challenge [9]. Exercise involves many pathways and factors, including AMP‐activated protein kinase (AMPK), peroxisome proliferator‐activated receptor delta (PPARδ) and myostatin. However, targeting these alone may not fully replicate the benefits of exercise, and the adverse effects of traditional targets are still a challenge to overcome [11]. Therefore, the lack of well‐defined therapeutic targets may have contributed to the absence of clinically approved exercise mimetics to date.

Prokineticin receptor 1 (PROKR1), a G protein‐coupled receptor with prokineticin 2 (PK2) as an intrinsic ligand, plays a vital role in metabolism [12]. PROKR1 activation inhibits adipocyte proliferation and promotes angiogenesis [13, 14, 15]. It also enhances insulin sensitivity via PI3K/AKT signalling in skeletal muscles [16]. Recently, we found that PROKR1 induces NR4A2 expression, leading to the oxidative muscle fibre specification [17]. These metabolic functions of PROKR1 were observed in the normal chow condition but were more pronounced in HFD condition. Therefore, these findings indicate that targeting PROKR1 could potentially mimic some of the beneficial effects of exercise on muscle function and overall metabolism. Since 2012, a total of three PROKR1 synthetic agonists, including IS1 and IS20, have been investigated (http://www.cortellis.com/drugdiscovery/) [18]; however, they remain in the preclinical phase due to limitations in their physico‐chemical properties and toxicity concerns. Currently, no clinically applicable agonists are available.

In this study, we discovered celecoxib as a novel PROKR1 agonist, inducing oxidative muscle fibre properties by activating PROKR1‐NR4A2 signalling in mouse and human myotubes. Celecoxib exposure during the pre‐ and postnatal muscle development activated Prokr1 signalling, enhanced oxidative muscle fibres and improved muscle phenotype in offspring at weaning. Furthermore, these effects persisted in both male and female mice at 12 and 20 weeks old, even after discontinuation of celecoxib treatment, while they were fed on HFD. In summary, celecoxib offers promise as a novel PROKR1 agonist and a potential therapeutic option for treating muscular disorders.

## 2 Methods

### 2.1 Reagents

Recombinant human PK2 (PeproTech, 100‐46), IS20 (Mcule), deracoxib (MedChemExpress, 169590‐41‐4) and celecoxib (Sigma‐Aldrich, 169590‐42‐5) were purchased and used.

### 2.2 Drug Discovery

The connectivity between PK2 and chemical perturbations was investigated using Connectivity Map (CMap) [19]. CMap analysis was performed using the top 30 up‐ and down‐regulated genes by PK2 (GSE148443) [16], and we selected approved drugs with ATC codes based on muscle‐related indications, oral administration and high connectivity score with PK2.

### 2.3 Molecular Docking

We performed molecular docking between the protein structure of human PROKR1, obtained from AlphaFold2 [20] and each compound. For docking, we utilized the DiffDock engine (https://github.com/gcorso/DiffDock), which identifies potential binding sites without the need for a grid map. The docking process was conducted using DeepZema (https://deepzema.ai) [21].

### 2.4 Cell Culture

HEK293T cells (ATCC) were maintained in high glucose DMEM supplemented with 10% (v/v) FBS (Gibco, 16000044) and 1% (v/v) antibiotic–antimycotic solution (Gibco). C2C12 cells (ATCC) were cultured in low‐glucose DMEM containing 20% (v/v) FBS and 1% (v/v) antibiotic–antimycotic solution (Gibco) and differentiated into myotubes for 5 days using high‐glucose DMEM supplemented with 2% (v/v) horse serum (Gibco). Human skeletal muscle cells (HSKMCs) (PromoCell) were differentiated into myotubes for 8 days according to the manufacturer's instructions.

### 2.5 cAMP and Inositol 1‐Phosphate (IP1) Assays

PROKR1‐overexpressing and deficient HEK293T cells at a density of 7 × 10^4^ cells per well of 96‐well plate (Corning) were plated and cultured in high‐glucose DMEM containing 2% (v/v) FBS and 1% (v/v) antibiotic–antimycotic solution (Gibco) overnight at 37°C in the presence of 5% CO_2_. PK2, deracoxib and celecoxib in serum‐free DMEM supplemented with 0.5 mM IBMX (Sigma‐Aldrich, 28822‐58‐4) were added to cells at various concentrations and incubated for 1 h. cAMP levels were measured in cell lysate with Cyclic AMP XP assay kit (Cell Signaling, 4339S) according to manufacturer protocol. IP1 levels were measured in cell lysate with IP‐One Gq ELISA kit (Cisbio, 72IP1PEA) according to manufacturer's instruction.

### 2.6 Western Blotting

Cells and tissue samples were lysed in RIPA buffer (Thermo Fisher Scientific) with protease and phosphatase inhibitors (GenDEPOT). Protein was separated on 9% (w/v) SDS‐PAGE gel, transferred to 0.45 μm PVDF membrane (Thermo Fisher Scientific) and blocked with 5% (w/v) BSA (Sigma‐Aldrich). Primary antibodies (Table S1) were applied overnight at 4°C. HRP‐conjugated secondary antibodies (Invitrogen) were used and enhanced using a chemiluminescence kit (Bio‐Rad). Protein bands were visualized using ImageJ software [22].

### 2.7 siRNA Transfection

C2C12 cells were transfected with 50 μM of siRNA targeting *Prokr1* (Bioneer, Daejeon, Republic of Korea) using Lipofectamine RNAiMAX Reagent (Thermo Fisher Scientific). The cells were then maintained for 5 days in a differentiation medium containing 2% (v/v) horse serum (Gibco). siRNA‐scramble (Bioneer) was used as control.

### 2.8 Mitochondria Quantification

Cells and muscle tissues were stained with MitoTracker Red CMXRos for mitochondria (Cell Signaling, 9082), Phalloidin‐488 for actin filaments (Cell Signaling, 12935S) and DAPI for nuclei (Vector Laboratories). The fluorescence intensity was measured using the Cytation 5 (BioTek). ImageJ software was applied to quantify the number of mitochondria, normalized by the fluorescence intensity of the nuclear stain.

### 2.9 Fatty Acid Oxidation (FAO)

The fatty acid oxidation (FAO) activity was measured using the Fatty Acid Oxidation Assay kit (Assay Genie, BR00001) according to the manufacturer's instructions. Briefly, during the beta‐oxidation of octanoyl‐CoA to acetyl‐CoA, NAD+ is reduced to NADH. The generated NADH then reduces the tetrazolium salt, iodonitrotetrazolium, to form a red‐coloured formazan. The absorbance of formazan, measured at 492 nm, indirectly reflects the FAO activity. C2C12 and HSKMC cell lysates were centrifuged at maximal speed for 5 min, and the resulting supernatant was collected and transferred to a 96‐well plate (Corning). Each well was then added with 50 μL of reaction solution and incubated at 37°C for 2 h. The formation of formazan was subsequently measured at 492 nm.

### 2.10 Animals

Male (12 weeks old) and female (9 weeks old) C57BL6/N mice (*n* = 32 each) were acquired from Koatech. The mice were kept in controlled conditions (22°C ± 2°C temperature, 50% ± 10% humidity, 12‐h light–dark cycle). Female mice were mated and pregnancy was confirmed by vaginal smear. Pregnant mice were assigned to groups and fed a customized diet containing varying concentrations of celecoxib (Saeronbio) from Day 5 of gestation until weaning at 4 weeks old. Offspring received a 60 kcal% fat diet (Research Diets Inc.) until 20 weeks. Mice were euthanized at 4, 12 or 20 weeks old to observe the effects of celecoxib in neonatal, young adult and adult mice. All animal procedures followed Seoul National University Institutional Animal Care and Use Committee (IACUC) guidelines (approval number: SNU‐220504‐2‐1).

### 2.11 Dual‐Energy X‐Ray Absorptiometry (DEXA)

Body composition were evaluated using the InAlyzer dual‐energy x‐ray absorptiometry (DEXA) system (Medikors). Mice were anaesthetized with an intraperitoneal injection of 300 mg/kg avertin (Sigma‐Aldrich) during the procedure. The InAlyzer system utilizes radiation absorption measurements to accurately determine material density. The InAlyzer software calculates essential body composition parameters, including fat mass, lean mass and body fat percentage.

### 2.12 Grip Strength

The grip strength of mice was assessed using a digital grip‐strength meter (BIOSEB). The mice were lowered over the grid, ensuring their torso remained horizontal and only their forepaws contacted the grid. The mouse was gently pulled back by its tail to encourage a firm grip on the top portion of the grid while maintaining a horizontal torso position. Each mouse underwent three trials to ensure accuracy, and the peak force achieved was subsequently normalized based on the bodyweight.

### 2.13 Glucose Tolerance and Insulin Tolerance Tests (GTT and ITT) and Insulin Resistance

Mice were fasted for 6 h before the tests, and they were conducted a week apart. For glucose tolerance test (GTT), mice received an oral administration of 1.5 g/kg glucose (Sigma‐Aldrich), and blood glucose levels were monitored for 120 min using a glucometer (Roche). In ITT, 30 min after an oral administration of 1.5 g/kg glucose, mice were intraperitoneally given 1.0 U/kg insulin (Sigma‐Aldrich), and blood glucose levels were measured. The area under the curve (AUC) for glucose levels during both GTT and insulin tolerance test (ITT) was calculated using Prism software (GraphPad). To measure insulin resistance, insulin level was measured by ELISA (Crystal Chem). Homeostatic Model Assessment for Insulin Resistance (HOMA‐IR) was calculated by multiplying fasting insulin (mU/L) and fasting glucose (mg/dL) and dividing the result by 405.

### 2.14 Indirect Calorimetry

Mice were housed individually in metabolic cages (TSE Systems). After a 2‐day acclimatization, they were monitored for 72 h. Energy expenditure (EE) was estimated using the Weir equation [23]: EE (kcal/day) = (VO_2_ × 3.941 + VCO_2_ × 1.106) × 1440. Ambulatory activity was assessed by counting interruptions via an infrared beam system in both the X‐ and Y‐axes. Data from the final 48 h of indirect calorimetry and ambulatory activity were used for analysis.

### 2.15 Immunocytochemistry (ICC) and Immunohistochemistry (IHC)

For ICC, myotubes were fixed in 4% (w/v) paraformaldehyde (Biosesang) and permeabilized with TRIS‐buffered saline containing 0.1% (v/v) Tween 20 (Sigma‐Aldrich). For immunohistochemistry (IHC), after necropsy, skeletal muscle tissues were frozen using isopentane (Thermo Fisher Scientific) cooled by liquid nitrogen. Frozen sections with a thickness of 10 μm were prepared and mounted onto slides. Immunostaining was performed using primary antibodies specific to myosin heavy chain proteins. Subsequently, secondary antibodies (Table S1) were applied and counterstained with DAPI (Sigma‐Aldrich). Images were captured and analysed using Cytation 5 (BioTek), and the fluorescence intensity of each MYH was normalized to the DAPI signal.

### 2.16 Statistical Analysis

Data were analysed using Prism software (GraphPad). Parametric data were analysed using a one‐way analysis of variance (ANOVA) test, followed by Dunnett's post hoc test and non‐parametric data using the Kruskal–Wallis test followed by Dunn's post hoc test. A significance level of *p* < 0.05 was used to determine statistical significance. Statistical methods applied for each data are described in the figure legend.

## 3 Results

### 3.1 Celecoxib Was Identified as a Novel Synthetic Agonist of PROKR1

**FIGURE 1:**
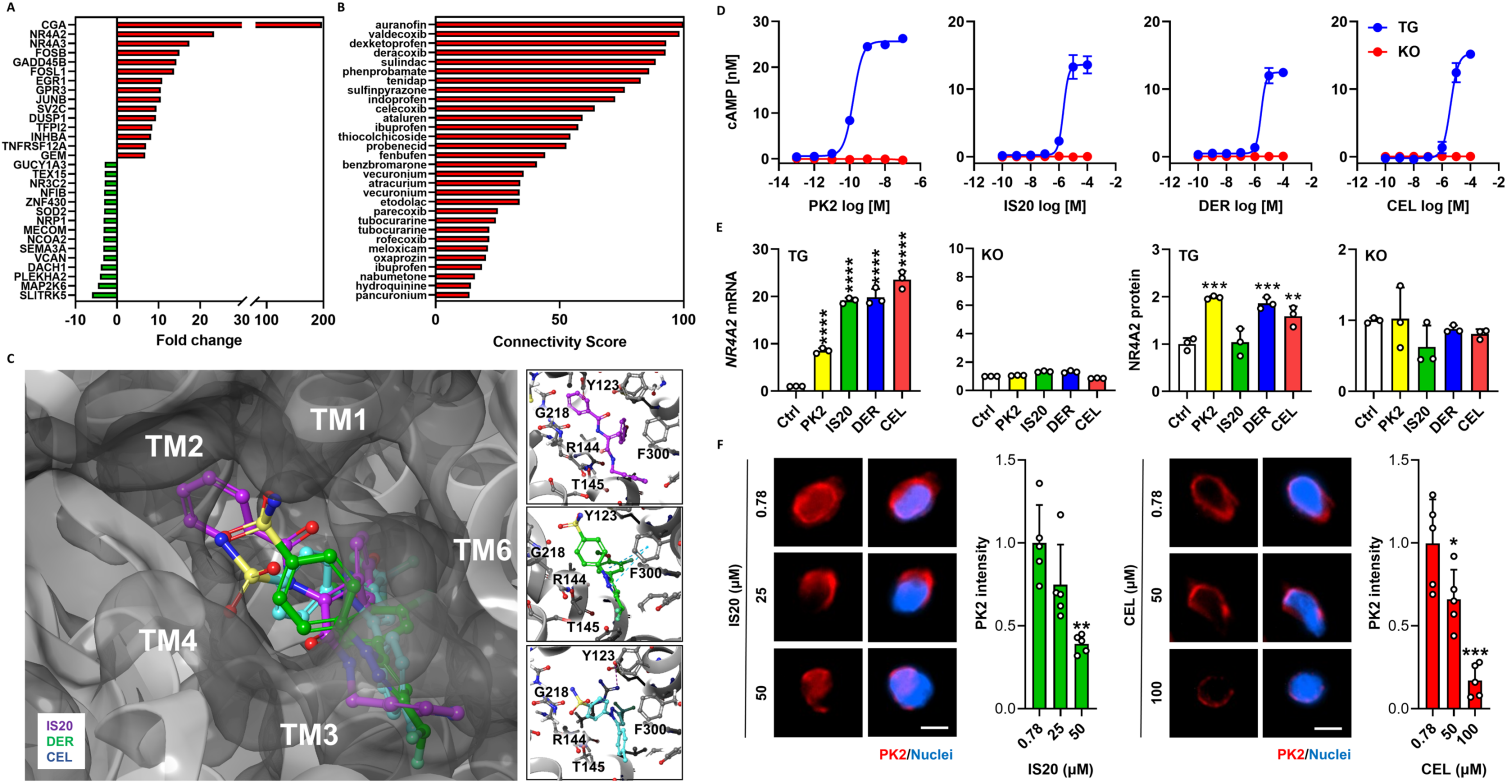
Discovery of PROKR1 agonists. (A) CMap query gene set. X‐axis indicates fold change after PK2 treatment in human PROKR1‐overexpressing HEK293T cells, Y‐axis for the top 15 up‐regulated (red bars) and down‐regulated genes (green bars), respectively. (B) Connected drugs to PK2. X‐axis indicates connectivity score between PK2 and each drug. (C) Molecular docking of coxibs to human PROKR1. Top view of the predicted protein shows IS20 (purple), deracoxib (DER, green) and celecoxib (CEL, cyan) bound to a binding pocket surrounded by the transmembrane domain (TM) of human PROKR1. The boxes on the right show the binding pose of IS20 (top), deracoxib (middle) and celecoxib (bottom), and the interacting residues of human PROKR1 are represented by single letter amino acid symbols (F: phenylalanine, G: glycine, R: arginine, T: threonine, Y: tyrosine). (D) cAMP response. cAMP accumulation is measured using human PROKR1‐overexpressing (TG) or deficient (KO) cells at different concentrations of each compound. (E) NR4A2 mRNA and protein expression. Values are mean ± SD, *n* = 3. *p < 0.05, **p < 0.01, ***p < 0.001, ****p < 0.0001 vs. control group (Ctrl), one‐way ANOVA followed by Dunnett's post hoc test. (F) Competitive assay to PK2. Red signals indicate PK2 and blue for nuclei. Scale bar is 10 μm. Values are mean ± SD, *n* = 8. *p < 0.05, **p < 0.01, ***p < 0.001 vs. 0.78 μM of each compound, Kruskal–Wallis test followed by Dunn's post hoc test.

Drugs with biological properties of PK2 were discovered through CMap analysis using PK2 signature genes (Figure 1A). Selection criteria included musculoskeletal indications and long‐term safety. Among 28 candidates with high connectivity scores to PK2 (Table S2), five were coxibs: valdecoxib (98.33), deracoxib (92.77), celecoxib (64.19), parecoxib (25.09) and rofecoxib (21.64) (Figure 1B). Molecular docking with PROKR1 protein revealed parecoxib with the highest docking score (−9.1), followed by celecoxib (−9.0), deracoxib, valdecoxib (−8.3) and rofecoxib (−8.0), comparable to IS20 (−9.0). The coxib binding pocket was located near the PROKR1 transmembrane domains and interacted with specific residues (Y123, R144, T145, G218 and F300) that corresponded to the same residues that IS20 interacted with (Figure 1C and Figure S1). This binding mode resembled the AVITGA motif crucial for PK2‐PROKR1 binding [24].

The on‐target effect of each compound was investigated. In PROKR1‐overexpressing HEK293T cells, PK2 activated Gs and Gq signal with EC_50_ values of 0.1 and 3 nM, respectively. In contrast, IS20, deracoxib and celecoxib only activated Gs signal with EC_50_ values of 2, 3 and 4 μM, respectively, and this effect was not observed in PROKR1‐deficient cells (Figure 1D and Figure S2). Treatment with PK2, IS20, deracoxib and celecoxib also significantly increase in NR4A2 expression only in PROKR1‐overexpressing cells. PK2 induced an 8.5‐fold increase in *NR4A2* mRNA, whereas IS20, deracoxib and celecoxib induced 19.1‐fold, 19.8‐fold and 23.5‐fold increases, respectively (*p* < 0.0001). Similarly, PK2 increased NR4A2 protein levels by 2.0‐fold (*p* < 0.001), whereas deracoxib and celecoxib increased NR4A2 protein levels by 1.9 (*p* < 0.001) and 1.6‐fold (*p* < 0.01), respectively. IS20 did not alter NR4A2 protein level (Figure 1E). IS20 and celecoxib dose‐dependently competed with PK2 to reduce cAMP accumulation by 11.9 and 1.9‐fold (*p* < 0.001), respectively (Figure S3), and reduced PK2 binding to PROKR1 by 2.6 (*p* < 0.01) and 5.9‐fold (*p* < 0.001), respectively (Figure 1F). This indicated that celecoxib would be an orthosteric modulator for PRORK1. Thus, celecoxib, discovered through drug repositioning approach, was shown to activate PROKR1 signalling in a PROKR1‐dependent manner, comparable to the natural ligand of PROKR1.

### 3.2 Celecoxib Induced Oxidative Muscle Fibre Specification Through PROKR1‐NR4A2 Signalling Activation

The effects of coxibs on myogenic differentiation were evaluated using murine (C2C12) and human myocytes (HSKMC). In differentiated C2C12 myotubes, celecoxib increased the protein levels of Prokr1 (1.8‐fold, *p* < 0.05), phosphorylated Creb (pCreb) (1.5‐fold, *p* < 0.05), Nr4a2 (1.3‐fold, *p* < 0.05), Pgc1a (1.7‐fold, *p* < 0.05), Tfam (4.2‐fold, *p* < 0.0001) and Myh7 (1.3‐fold, *p* < 0.05) compared to vehicle control (Figures S4A and S5). Myh4 and Myh2 expression remained unchanged (Figure S6). Immunocytochemistry confirmed a 2.2‐fold increase in Myh7‐positive myotube formation (*p* < 0.0001) (Figure S4B). On the other hand, Myh4 and Myh2 expression decreased significantly (*p* < 0.05) (Figure S7). The fusion index supported these results. Myh7‐positive myofibers were significantly increased in all treatments, with more than 34.1% compared to 19.7% in vehicle control (*p* < 0.0001) (Figure S8). Celecoxib increased the mitochondrial respiratory chain complex II–V levels, particularly complex V (3.1‐fold, *p* < 0.01) (Figure S4C), and increased the mitochondrial content by 1.6‐fold (*p* < 0.001) (Figures S4D and S9). This enhancement led to a 4.1‐fold increase in FAO activity compared to vehicle control (*p* < 0.05) (Figure S4E).

**FIGURE 2:**
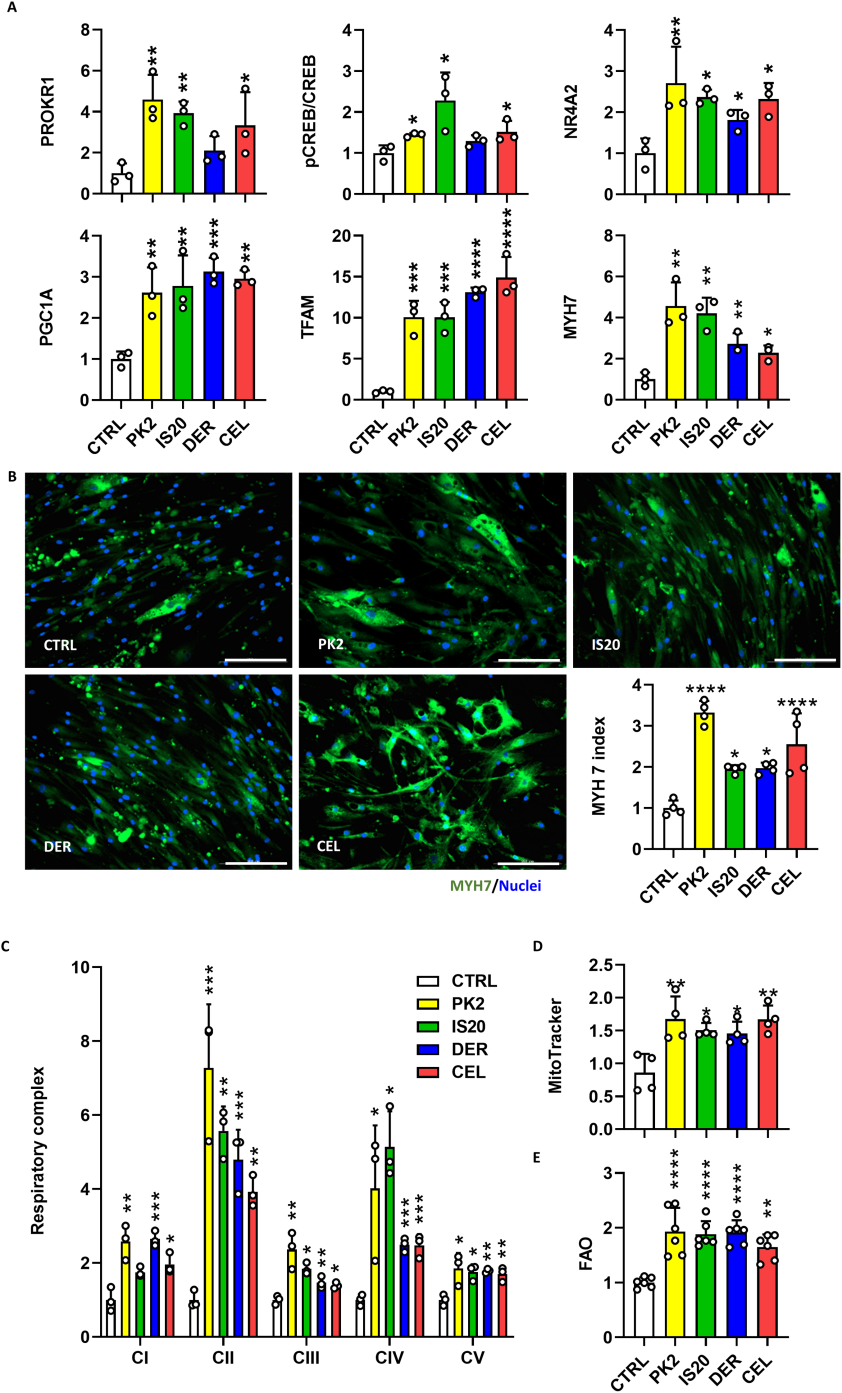
Coxib‐induced oxidative muscle fibre specification. (A) PROKR1 signalling in HSKMC myotubes. (B) Immunocytochemistry of HSKMC myotubes. Scale bar is 200 μm. (C) Respiratory chain complex in HSKMC myotubes. (D) Mitochondrial content in HSKMC myotubes. (E) FAO activity in HSKMC myotubes. CEL, celecoxib; CTRL, vehicle control; DER, deracoxib. Values are mean ± SD, *n* = 3. *p < 0.05, **p < 0.01, ***p < 0.001, ****p < 0.0001 vs. control group (CTRL), one‐way ANOVA followed by Dunnett's post hoc test (A,C,D,E) or Kruskal–Wallis test followed by Dunn's post hoc test (B).

The myogenic effects of coxibs were verified in human myotubes. Specifically, celecoxib increased PROKR1 (3.3‐fold, *p* < 0.05), pCREB (1.5‐fold, *p* < 0.05), NR4A2 (2.3‐fold, *p* < 0.05), PGC1A (3.0‐fold, *p* < 0.01), TFAM (14.9‐fold, *p* < 0.0001) and MYH7 (2.3‐fold, *p* < 0.05) protein levels (Figure 2A and Figure S10). MYH2 increased by 1.3‐fold (*p* < 0.05) without altering MYH4 (Figure S11). Immunocytochemistry confirmed a 2.6‐fold increase in MYH7 (*p* < 0.0001) (Figure 2B) and 2.2‐fold decrease in MYH4 (*p* < 0.05) (Figure S12). In addition, all treatments significantly increased the fusion index of MYH7‐positive myofibers to over 45.8% compared to 29.1% in vehicle control (*p* < 0.01) (Figure S13). Celecoxib increased complex I–V levels, a particularly 3.9‐fold increase in complex II (*p* < 0.01) (Figure 2C), and increased mitochondrial content by 1.7‐fold (*p* < 0.01) (Figure 2D and Figure S14). This enhancement led to a 1.7‐fold increase in FAO activity compared to vehicle control (*p* < 0.01) (Figure 2E). In contrast, both Prokr1 signalling activity and Myh7‐positive oxidative muscle fibre specification by celecoxib were attenuated in *Prokr1* knock‐downed muscle cells (Figure S15). Therefore, these results suggest that celecoxib activates PROKR1 signalling in both mouse and human myotubes, which promotes oxidative muscle fibre specification.

### 3.3 Pre‐ and Postnatal Treatment With Celecoxib Improved the Muscle Phenotypes of Neonatal Mice at 4 Weeks Old

**FIGURE 3:**
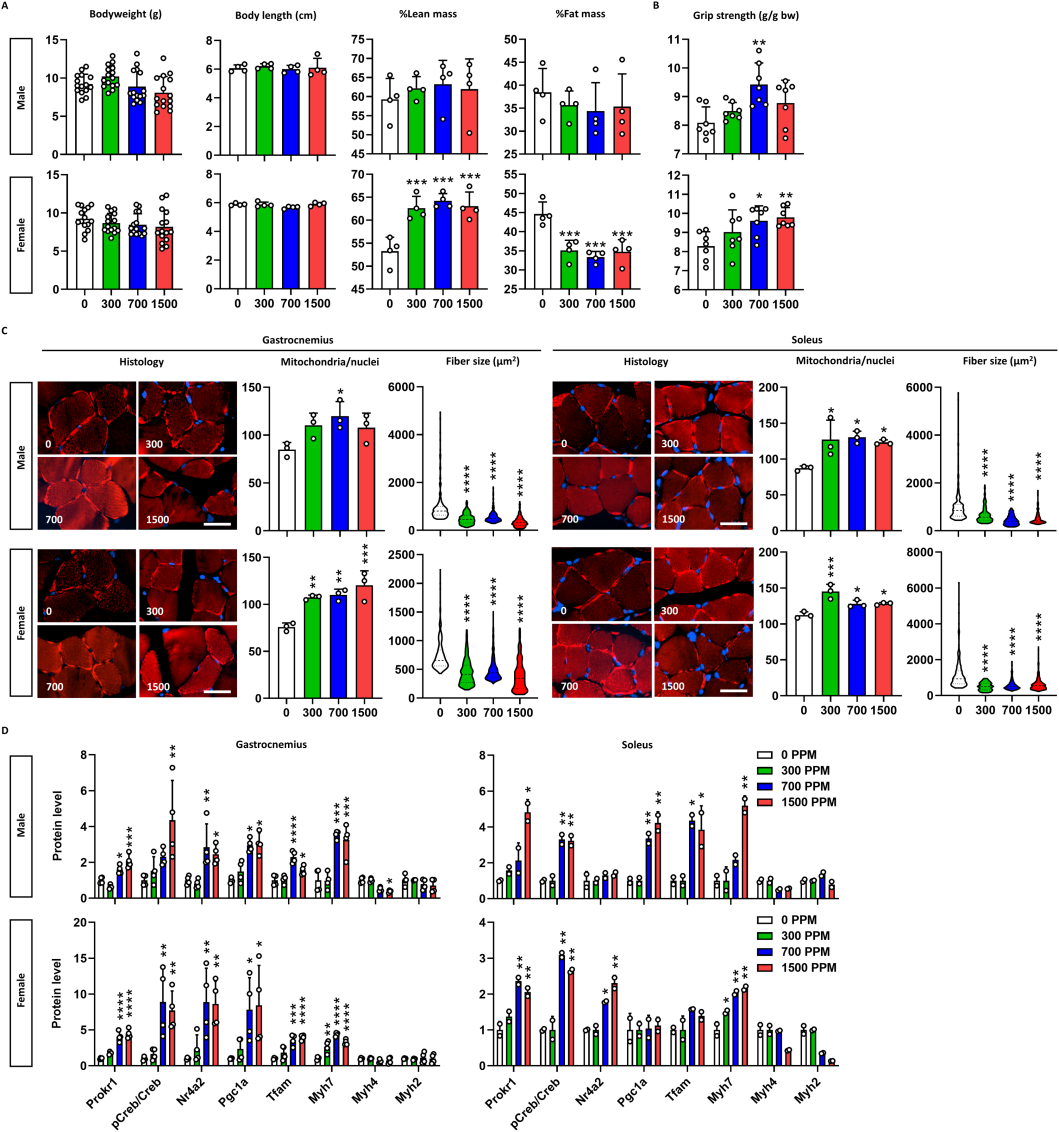
Muscular phenotypes of neonatal mice. (A) Body composition. Bodyweight (*n* = 15), body length, %lean mass and %fat mass of mice (*n* = 4) are depicted. X‐axis denotes the concentration of celecoxib added to the diet (ppm). (B) Grip strength. Grip strength of mice (*n* = 7) are corrected for bodyweight. (C) Muscle fibre composition. Histology shows the mitochondria (red) and nuclei (blue) of muscle fibres. Scale bar is 100 μm. Mitochondrial content is given by the mitochondria count divided by the nuclei count (*n* = 3), and fibre size is the area of each fibre (*n* ≥ 229). Data are presented as violin plots with the median, quartiles and distribution of the values. (D) Prokr1 signalling. Protein levels of Prokr1, phosphorylated Creb (pCreb), Nr4a2, Pgc1a, Tfam, Myh7, Myh4 and Myh2 are depicted (*n* = 3). Values are mean ± SD. *p < 0.05, **p < 0.01, ***p < 0.001, ****p < 0.0001 vs. 0 ppm, Kruskal–Wallis test followed by Dunn's post hoc test for fibre size and one‐way ANOVA followed by Dunnett's post hoc test for others.

Celecoxib, preferred over deracoxib due to its suitability for human use, was chosen for an in vivo pharmacological study. Pregnant dams were fed diets containing varying celecoxib concentrations (0, 500, 1000 and 1500 ppm) from gestation day 5 through weaning to indirectly expose fetuses during muscle development. At weaning, offspring showed improved muscle phenotype without affecting bodyweight or length compared to vehicle controls (Figure 3A). Body composition analysis revealed dose‐dependent increases in lean mass and decreases in fat mass only in females (more than 9.35% increase in lean mass and 9.49% decrease in fat mass, *p* < 0.001) (Figure 3A and Figure S16). Grip strength also increased significantly in males (up to 16.6%, *p* < 0.01 at 700 ppm) and females (up to 18.0%, *p* < 0.01 at 1500 ppm), correlating with increased lean body mass and muscle weight. Interestingly, this was accompanied by an increase in muscle fibres per unit area of muscle tissue, despite a reduction in muscle fibre size, suggesting that changes in fibre density and organization may play a role in accounting for the observed strength enhancement (Figure 3B and Figure S17). Macroscopic examination indicated a dose‐dependent enhancement of thigh muscle redness (Figure S18).

Histological examination revealed that celecoxib induced the myofibers to acquire oxidative muscle fibre properties. Celecoxib significantly increased mitochondrial content in the gastrocnemius muscle of both sexes, with males up to 41.3% at 700 ppm (*p* < 0.05) and females up to 58.6% at 1500 ppm (*p* < 0.001) compared to vehicle controls (Figure 3C). Muscle fibre size decreased in a dose‐dependent manner, with reductions up to 64.0% in males and 52.1% in females at 1500 ppm (*p* < 0.0001) (Figure S19). Similar changes were observed in the soleus muscle. These alterations, marked by increased mitochondrial content and decreased fibre size, indicate a shift towards oxidative muscle fibre formation due to fetal exposure to celecoxib during muscle development.

To explore the role of Prokr1 signalling in the myofiber composition changes, we measured the protein levels of the signalling molecules. In the gastrocnemius muscle of male offspring, celecoxib dose‐dependently increased the expression of Prokr1 signalling molecules, especially at 1500 ppm, increasing Prokr1 (2.1‐fold, *p* < 0.001), pCreb (4.4‐fold, *p* < 0.01), Nr4a2 (2.5‐fold, *p* < 0. 05), Pgc1a (3.1‐fold, *p* < 0.05) and Tfam (1.6‐fold, *p* < 0.05) (Figure 3D). Myh7 increased by celecoxib (3.3‐fold, *p* < 0.001), whereas Myh4 and Myh2 remained unchanged. This dose‐dependent activation of the Prokr1 signal was also observed in female gastrocnemius and partially in the soleus muscle of both sexes (Figures S20 and S21). Taken together, celecoxib exposure during muscle development activates Prokr1 signalling in offspring, promoting oxidative muscle fibre formation and enhancing muscle phenotype at weaning.

### 3.4 Improved Muscle Phenotypes by Celecoxib Were Maintained in Young Adult Mice at 12 Weeks Old

**FIGURE 4:**
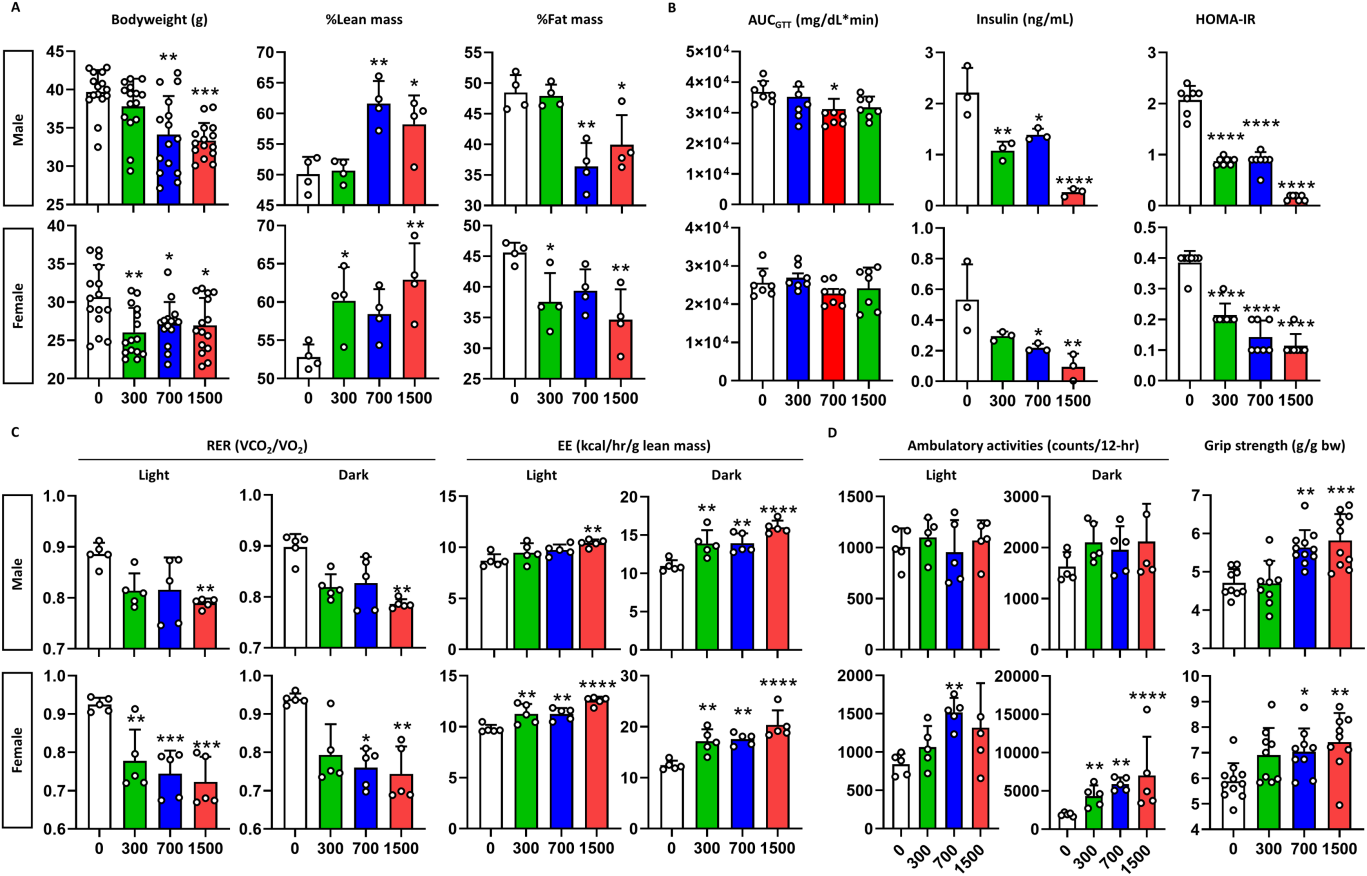
Metabolic phenotypes of young adult mice. (A) Body composition. Bodyweight (*n* = 15), %lean mass, and %fat mass of mice (*n* = 4) are depicted. X‐axis denotes the concentration of celecoxib added to the diet (ppm). (B) Insulin sensitivity. The graphs show, from left to right, area under the curve (AUC) of blood glucose after oral administration of glucose (*n* = 7), basal insulin level and homeostatic model assessment for insulin resistance (HOMA‐IR) (*n* = 3). (C) Energy expenditure. The graphs show the respiratory exchange ratio (RER) and the energy expenditure (EE) for the light and the dark phases (*n* = 5). (D) Exercise performance. In‐cage ambulatory activities for the light and the dark phases (*n* = 5) and bodyweight‐corrected grip strength (*n* = 9–11) are depicted. Values are mean ± SD. *p < 0.05, **p < 0.01, ***p < 0.001, ****p < 0.0001 vs. 0 ppm, Kruskal–Wallis test followed by Dunn's post hoc test for male GTT, male RER in both phases, female RER in dark phase, female ambulatory activity in light phase, western blot in male gastrocnemius (Pgc1a, Myh4 and Myh2), male soleus (Nr4a2, Myh4 and Myh2), female gastrocnemius (Pgc1a and Myh4), female soleus (Tfam, Myh4 and Myh2) and one‐way ANOVA followed by Dunnett's post hoc test for others.

To determine the mid‐term effects of pre‐ and postnatal exposure to celecoxib, we fed offspring HFD from weaning and analysed young adult mice at 12 weeks old. Both male and female mice exposed to celecoxib above 700 ppm exhibited reduced body weights from 7 weeks (*p* < 0.01) (Figure S22). By 12 weeks, a dose‐dependent decrease in bodyweight was evident, with a weight loss of 15.9% (*p* < 0.001) in males and 13.3% (*p* < 0.05) in females, especially at 1500 ppm of celecoxib compared to vehicle controls (Figure 4A). Celecoxib had no impact on food consumption (Figure S23). It increased lean mass and decreased fat mass in both sexes. Especially at 1500 ppm, lean body mass increased by 16.2% (*p* < 0.05) and 19.1% (*p* < 0.01) and fat mass decreased by 17.6% (*p* < 0.05) and 23.9% (*p* < 0.01) in males and females, respectively (Figure 4A and Figure S24). Whereas no glucose or insulin tolerance improvement was seen (Figures S25 and S26), insulin resistance (HOMA‐IR) showed a dose‐dependent improvement, with a 92.5% and 70.4% in males and females at 1500 ppm of celecoxib, respectively (*p* < 0.0001) (Figure 4B and Figure S27). Celecoxib shifted the primary energy source from carbohydrates to fats in both sexes (Figure 4C and Figure S28), enhancing energy expenditure by 46.0% and 63.7% in males and females, particularly at 1500 ppm in the dark phase (*p* < 0.0001) (Figure 4C and Figure S29). Ambulatory activities increased significantly, particularly in females, and grip strength increased dose‐dependently in males and females by 23.5% (*p* < 0.001) and 26.1% (*p* < 0.01) especially at 1500 ppm of celecoxib, respectively (*p* < 0.05) (Figure 4D). These findings suggest lasting metabolic effects of pre‐ and postnatal exposure of celecoxib on offspring.

**FIGURE 5:**
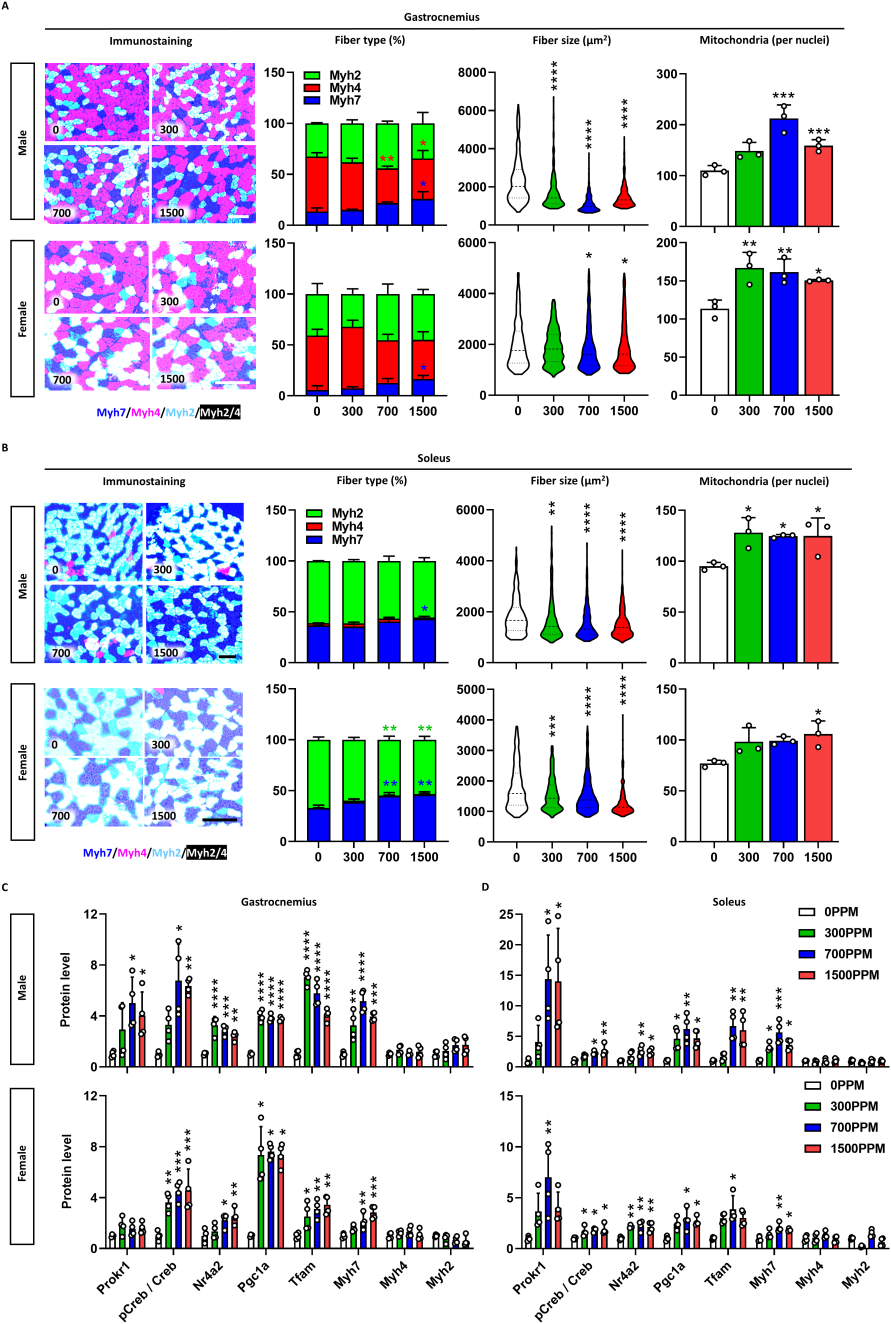
Muscular phenotypes of young adult mice. Characterization of gastrocnemius (A) and soleus muscle tissues (B). In immunostaining, dark blue indicates Myh7‐positive fibres, light red for Myh4‐positive fibres, light blue for Myh2‐positive fibres and white for Myh2/4 mixed fibres. The concentration of celecoxib added to the diet (ppm) is shown at the bottom left of each figure. Scale bar is 100 μm. Stacked bars represent the fibre composition by concentration, fibre size is presented as violin plots with the median, quartiles and distribution of the values (*n* ≥ 222), and mitochondrial content is given by the mitochondria count divided by the nuclei count (*n* = 3). Prokr1 signalling in gastrocnemius (C) and soleus muscle (D). Protein levels of Prokr1, phosphorylated Creb (pCreb), Nr4a2, Pgc1a, Tfam, Myh7, Myh4 and Myh2 are depicted (*n* = 3). Values are mean ± SD. *p < 0.05, **p < 0.01, ***p < 0.001, ****p < 0.0001 vs. 0 ppm, Kruskal–Wallis test followed by Dunn's post hoc test for western blot in male gastrocnemius (pCreb/Creb, Myh4 and Myh2), male soleus (pCreb/Creb, Myh4 and Myh2), female gastrocnemius (Pgc1a and Tfam), female soleus (Prokr1, Pgc1a, Tfam, Myh4 and Myh2) and one‐way ANOVA followed by Dunnett's post hoc test for others.

Consistent with the observed changes in body composition, a dose‐dependent increase in muscle weight and decrease in fat weight was observed in both sexes. At 1500 ppm of celecoxib, the gastrocnemius muscle weight increased by 28.6% (*p* < 0.01) and 24.8% (*p* < 0.05), whereas visceral fat weight decreased by 28.6% (*p* < 0.05) and 68.6% (*p* < 0.001) in males and females, respectively (Figure S30). Celecoxib exposure increased Myh7‐positive muscle fibres in the gastrocnemius muscles of male and female mice by 12.3% and 10.8% (*p* < 0.05), respectively (Figure 5A). It reduced muscle fibre size dose‐dependently, decreasing by 35.2% (*p* < 0.0001) in males and 5.4% (*p* < 0.05) in females at 1500 ppm (Figure S31) and increased mitochondrial content by 44.7% (*p* < 0.001) and 32.8% (*p* < 0.05), respectively (Figure S32). Moreover, celecoxib significantly reduced intramuscular lipid accumulation by 69% in male (*p* < 0.05) and by 64% in female at 1500 ppm (Figure S33), as evidenced by the decreased expression of perilipin 2 (Plin2), a well‐established marker of intramuscular triglyceride storage and lipid droplet [25, 26, 27]. Similarly, in soleus muscle tissues, celecoxib increased Myh7‐positive muscle fibres, reduced fibre size, and increased mitochondrial content in both sexes compared to vehicle controls (Figure 5B). Western blotting revealed that these changes were attributed to Prokr1 signal activation. In male gastrocnemius muscle tissues, celecoxib increased Prokr1 (4.0‐fold, *p* < 0.05), pCreb (6.3‐fold, *p* < 0.01), Nr4a2 (2.4‐fold, *p* < 0.01), Pgc1a (3.7‐fold, *p* < 0.0001), Tfam (4.1‐fold, *p* < 0.0001) and Myh7 (4.0‐fold, *p* < 0.001), particularly at 1500 ppm (Figure 5C and Figure S34). Similar increases were observed in male soleus (Figure 5D) and female muscle tissues (Figure S35).

### 3.5 Improved Muscle Phenotypes by Celecoxib Were Maintained in Adult Mice at 20 Weeks Old

**FIGURE 6:**
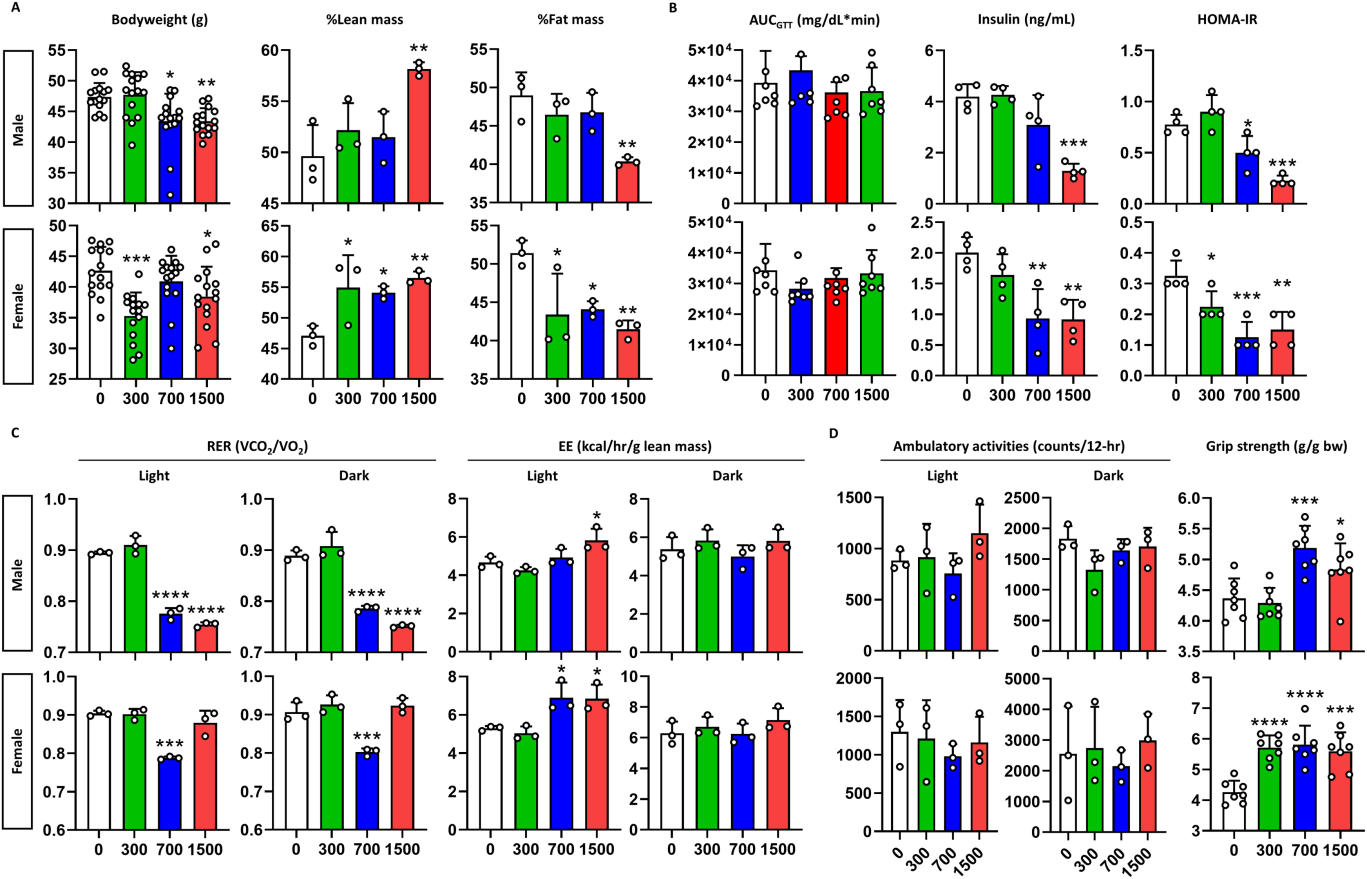
Metabolic phenotypes of adult mice. (A) Body composition. Bodyweight (*n* = 15), %lean mass and %fat mass of mice (*n* = 3) are depicted. X‐axis denotes the concentration of celecoxib added to the diet (ppm). (B) Insulin sensitivity. The graphs show, from left to right, area under the curve (AUC) of blood glucose after oral administration of glucose (*n* = 7), basal insulin level and homeostatic model assessment for insulin resistance (HOMA‐IR) (*n* = 4). (C) Energy expenditure. The graphs show the respiratory exchange ratio (RER) and the energy expenditure (EE) for the light and the dark phases (*n* = 3). (D) Exercise performance. In‐cage ambulatory activities for the light and the dark phases (*n* = 3) and bodyweight‐corrected grip strength (*n* = 7) are depicted. Values are mean ± SD. *p < 0.05, **p < 0.01, ***p < 0.001, ****p < 0.0001 vs. 0 ppm, Kruskal–Wallis test followed by Dunn's post hoc test for male bodyweight, and one‐way ANOVA followed by Dunnett's post hoc test for others.

In 20‐week‐old adult mice continuously fed HFD, celecoxib induced significant reductions in bodyweight by 8.3% (*p* < 0.01) in males and 9.8% (*p* < 0.05) in females, particularly at 1500 ppm (Figure 6A and Figure S36). Food consumption fluctuated but showed no overall difference (Figure S37). Celecoxib dose‐dependently altered body composition, with 1500 ppm particularly increasing lean mass by 22.1% and 20.0% (*p* < 0.01) and decreasing fat mass by 17.6% and 19.3% (*p* < 0.01) in males and females, respectively (Figure 6A and Figure S38). Glucose and insulin tolerance remained unchanged (Figures S39 and S40), but basal insulin levels decreased significantly, and HOMA‐IR improved consistently in both sexes (Figure 6B and Figure S41). Celecoxib maintained fat as the primary energy source and significantly increased EE by 24.8% and 29.1% in male and female mice, especially at 1500 ppm in light phase (*p* < 0.05), respectively (Figure 6C and Figures S42 and S43). The effect on ambulatory activity was attenuated, whereas celecoxib increased grip strength in adult mice by 10.8% (*p* < 0.05) in males and 31.3% (*p* < 0.001) in females, particularly at 1500 ppm (Figure 6D).

**FIGURE 7:**
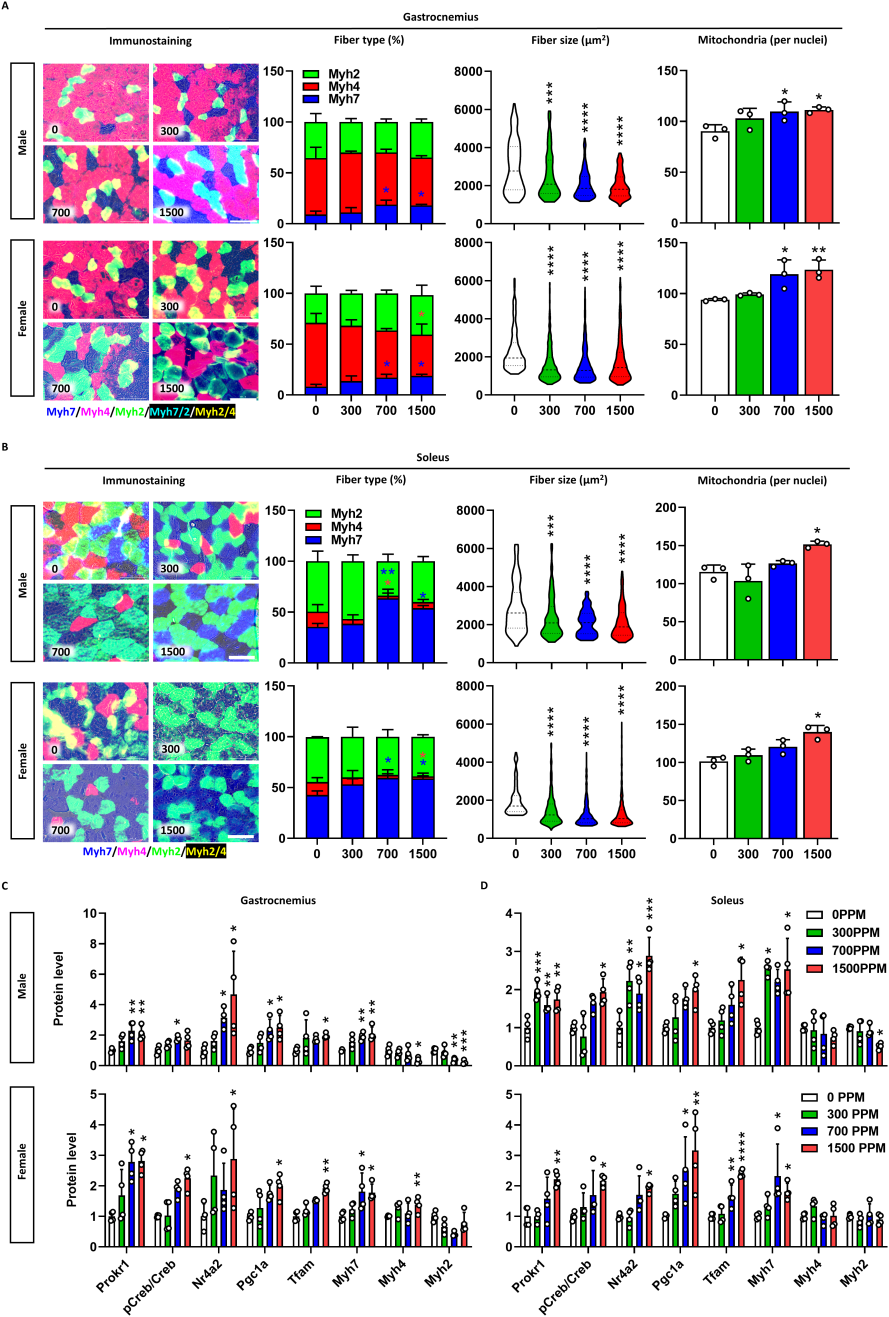
Muscular phenotypes of adult mice. Characterization of gastrocnemius (A) and soleus muscle tissues (B). In immunostaining, dark blue indicates Myh7‐positive fibres, light red for Myh4‐positive fibres, light blue for Myh2‐positive fibres, light blue for Myh7/2 mixed fibres and yellow for Myh4/2 mixed fibres. The concentration of celecoxib added to the diet (ppm) is shown at the bottom left of each figure. Scale bar is 200 μm. Stacked bars represent the fibre composition by concentration, fibre size is presented as violin plots with the median, quartiles and distribution of the values (*n* ≥ 123), and mitochondrial content is given by the mitochondria count divided by the nuclei count (*n* = 3). Prokr1 signalling in gastrocnemius (C) and soleus muscle (D). Protein levels Prokr1, phosphorylated Creb (pCreb), Nr4a2, Pgc1a, Tfam, Myh7, Myh4 and Myh2 are depicted (*n* = 3). Values are mean ± SD. *p < 0.05, **p < 0.01, ***p < 0.001, ****p < 0.0001 vs. 0 ppm, Kruskal–Wallis test followed by Dunn's post hoc test for western blot in male gastrocnemius (pCreb/Creb, Nr4a2 and Tfam), male soleus (pCreb/Creb, Pgc1a, Tfam, Myh7, Myh4 and Myh2), female gastrocnemius (Prokr1, pCreb/Creb, Nr4a2, Pgc1a and Tfam), female soleus (pCreb/Creb, Nr4a2, Pgc1a and Myh7) and one‐way ANOVA followed by Dunnett's post hoc test for others.

Consistent with the observed changes in body composition, male and female adult mice at 1500 ppm of celecoxib showed an 11.0% (*p* < 0.05) and 30.3% (*p* < 0.01) increase in gastrocnemius muscle weight and a 23.4% and 32.2% (*p* < 0.05) decrease in subcutaneous fat weight, respectively (Figure S44). Celecoxib increased Myh7‐positive muscle fibres in the gastrocnemius, particularly by 9.1% and 10.6% (*p* < 0.05) at 1500 ppm (Figure 7A), and reduced muscle fibre size (Figure S45), whereas mitochondrial content increased dose‐dependently (Figure S46). Intramuscular fat accumulation was also significantly reduced in both male and female mice at all dose levels (> 48%, *p* < 0.01) (Figure S47). In soleus muscle, Myh7‐positive fibres increased, fibre size decreased, and mitochondrial content increased significantly in both sexes (Figure 7B). Celecoxib dose‐dependently increased Prokr1 signalling activity in male gastrocnemius muscle tissue, with Prokr1 (2.1‐fold, *p* < 0. 01), pCreb (1. 7‐fold), Nr4a2 (4.7‐fold, *p* < 0.05), Pgc1a (2.5‐fold, *p* < 0.05), Tfam (1.9‐fold, *p* < 0.05) and Myh7 (2.1‐fold, *p* < 0.01) significantly increased at 1500 ppm (Figure 7C and Figure S48). Similar increases were observed in male and female soleus muscle tissues (Figure 7D and Figure S49). Taken together, these results suggest that pre‐ and postnatal exposure to celecoxib induces oxidative muscle fibre formation through activation of Prokr1 signalling, leading to mid‐ and long‐term improvements in muscle and metabolic function in the offspring.

## 4 Discussion

Despite successful therapeutic target families, the development of GPCR‐specific modulators is challenging due to the limited protein structure knowledge [28, 29, 30]. For similar reasons, PROKR1‐specific agonists have not been clinically developed. Connectivity maps (https://clue.io/) offer a robust opportunity to overcome these limitations [19, 31]. To identify PROKR1‐targeted agonists, we applied the PK2 transcriptional signature to the connectivity map. This analysis identified coxibs, particularly celecoxib, as potential PROKR1 agonists. Cell‐based assays confirmed their PROKR1‐dependent activity. Therefore, celecoxib could be repurposed as a therapeutic agent for PROKR1 activation, with clinical potential due to its existing approval for human use.

Celecoxib, a well‐known cyclooxygenase‐2 (COX‐2) inhibitor and non‐steroidal anti‐inflammatory drug [32, 33], has shown promise in benefiting muscle health by protecting against muscle atrophy and promoting muscle mass gains. Studies have revealed positive outcomes, such as weight gain and improved quality of life in cachectic cancer patients taking celecoxib [34]. Long‐term use also resulted in increased lean body mass, improved grip strength and better performance status [35]. Celecoxib has also been reported to improve age‐related muscle loss and muscle function [36]. Although its anti‐inflammatory properties explain some benefits, the full scope of its effects on muscle tissue that does not express COX‐2 remains unclear [37]. In this regard, the present study is of great significance, as it demonstrated a novel mechanism of action of celecoxib via PROKR1 in skeletal muscle.

Celecoxib was found to enhance muscle development and phenotypes by activating PROKR1 signalling. It induced oxidative muscle fibre specification through PROKR1‐NR4A2 activation, benefiting neonates and adult mice. These enduring effects suggest the imprinting effect of celecoxib on skeletal muscle during development. Studies on maternal exercise and substances like resveratrol and metformin in pregnant rats indicate prenatal factors influence long‐term health outcomes [38, 39, 40, 41]. Therefore, celecoxib‐induced PROKR1 signalling presents an exciting avenue for enhancing muscle development and improving muscle phenotypes. Further investigations are warranted to unravel the precise mechanisms involved and to explore the broader metabolic implications of celecoxib‐PROKR1 signalling, which may have implications for therapeutic interventions in muscle‐related conditions and metabolic disorders.

This study has limitations to acknowledge. Firstly, there was no direct evidence confirming whether the effects of celecoxib on muscle and metabolic phenotypes stem from COX‐2 inhibition and/or PROKR1 activation. However, given the muscle tissue‐specific expression of Prokr1, and the muscular effects of celecoxib that are kept in adult mice despite discontinuation of celecoxib after weaning, the effects of celecoxib appear to be dependent on Prokr1 activity imprinted during muscle development. Another limitation is the ongoing debate over the specific metabolic mechanisms of celecoxib. Although this study suggests metabolic improvements could result from muscle remodelling, fat loss is also conceivable. We have previously observed decreased expression of Prokr1 in both muscle and adipose tissue in HFD‐fed mice, suggesting that the long‐term effects of celecoxib likely involve reconstructed muscle rather than fat. However, both fat and muscle influence systemic metabolism, raising the possibility of muscle‐fat cross‐talk in celecoxib‐induced effects. Therefore, further studies are needed to fully understand the metabolic mechanisms of celecoxib and the roles of muscle remodelling and fat loss in these processes. Lastly, though the current study focuses on an HFD model to reveal the pharmacologic effects of celecoxib, it remains to be seen how these effects might manifest under a normal chow. Given that *Prokr1*‐deficient mice exhibit some metabolic disturbances even on a normal chow diet [17], it is plausible that celecoxib might still confer benefits, albeit to a lesser extent. Future studies should explore the effects of celecoxib administered postnatally or concomitant with a traditional diet to fully understand its therapeutic potential.

In conclusion, the results presented in this study highlight the therapeutic potential of celecoxib as a PROKR1 agonist. Celecoxib was effective in enhancing oxidative muscle fibre properties and improving overall muscle phenotype; therefore, celecoxib holds promise as a novel PROKR1 agonist and clinically applicable therapeutic agent for the treatment of muscle diseases.

## Author Contributions

Conceptualization: J.H.P., J.M. and J.P. Methodology: J.H.P. and J.M. Investigation: J.H.P., J.M., S.P., D.K., M.K. and T.S.P. Formal analysis: J.H.P., J.M., M.P. and J.P. Writing – original draft: J.H.P. and J.P. Writing – revision: J.H.P., J.M. and J.P. Data curation: J.H.P. and J.P. Funding acquisition: J.P. Supervision: J.P. J.P. is the guarantor of this work and, as such, had full access to all the data in the study and takes responsibility for the integrity of the data and the accuracy of the data analysis.

## Conflicts of Interest

The authors declare no conflicts of interest.

## Supporting information


**Figure S1.** Molecular docking of coxibs to human PROKR1.
**Figure S2.** IP1 accumulation response in HEK293T cells.
**Figure S3.** cAMP competition assay.
**Figure S4.** Coxib‐induced oxidative muscle fibre specification.
**Figure S5.** Western blotting of Prokr1 signalling molecules in C2C12 myotubes.
**Figure S6.** Quantification of Myh4 and Myh2 in C2C12 myotubes.
**Figure S7.** Immunocytochemistry of Myh4 and Myh2 in C2C12 myotubes.
**Figure S8.** Total nuclei number and fusion index for C2C12 myotubes.
**Figure S9.** Mitochondrial mass in C2C12 myotubes.
**Figure S10.** Western blotting of PROKR1 signalling molecules in HSKMC myotubes.
**Figure S11.** Quantification of MYH4 and MYH2 in HSKMC myotubes.
**Figure S12.** Immunocytochemistry of MYH4 and MYH2 in HSKMC myotubes.
**Figure S13.** Total nuclei number and fusion index for HSKMC myotubes.
**Figure S14.** Mitochondrial mass in HSKMC myotubes.
**Figure S15.** Attenuation of celecoxib‐induced oxidative muscle fibre specification in *Prokr1* knock‐downed mouse myotubes.
**Figure S16.** Body composition of neonatal mice.
**Figure S17.** Relative muscle weights and muscle fibre counts of neonatal mice.
**Figure S18.** Macroscopic examination of thigh muscle.
**Figure S19.** H&E staining of neonate muscle tissues.
**Figure S20.** Western blotting of Prokr1 signal molecules in male neonate muscle tissues.
**Figure S21.** Western blotting of Prokr1 signal molecules in female neonate muscle tissues.
**Figure S22.** Bodyweight changes of young adult mice.
**Figure S23.** Food consumption of young adult mice.
**Figure S24.** Body composition of young adult mice.
**Figure S25.** Glucose tolerance of young adult mice.
**Figure S26.** Insulin tolerance of young adult mice.
**Figure S27.** Area under the glucose curve during ITT and basal (fasting) glucose levels of young adult mice.
**Figure S28.** Respiratory exchange ratio of young adult mice.
**Figure S29.** Energy expenditure of young adult mice.
**Figure S30.** Organ weights of young adult mice.
**Figure S31.** H&E staining of young adult mice muscle tissues.
**Figure S32.** Mitochondria mass in young adult mice muscle tissues.
**Figure S33.** Lipid content in young adult mice muscle tissues.
**Figure S34.** Western blotting of Prokr1 signalling molecules in male young adult mice muscle tissues.
**Figure S35.** Western blotting of Prokr1 signalling molecules in female young adult mice muscle tissues.
**Figure S36.** Bodyweight changes of adult mice.
**Figure S37.** Food consumption of adult mice.
**Figure S38.** Body composition of adult mice.
**Figure S39.** Glucose tolerance of adult mice.
**Figure S40.** Insulin tolerance of adult mice.
**Figure S41.** Area under the glucose curve during ITT and basal (fasting) glucose levels of adult mice.
**Figure S42.** Respiratory exchange ratio of adult mice.
**Figure S43.** Energy expenditure of adult mice.
**Figure S44.** Organ weights of adult mice.
**Figure S45.** H&E staining of adult mice muscle tissues.
**Figure S46.** Mitochondria mass in adult mice muscle tissues.
**Figure S47.** Lipid content in adult mice muscle tissues.
**Figure S48.** Western blotting of Prokr1 signalling molecules in male adult mice muscle tissues.
**Figure S49.** Western blotting of Prokr1 signalling molecules in female adult mice muscle tissues

**Table S1** Antibody information.
**Table S2.** Connectivity map analysis result.
