# Supplementary material for: Celecoxib Enhances Oxidative Muscle Fibre Formation and Improves Muscle Functions Through Prokr1 Activation in Mice
Source: J Cachexia Sarcopenia Muscle. 2025 Jan 30;16(1):e13704. doi: 10.1002/jcsm.13704 (PMC11780397; doi:10.1002/jcsm.13704)

**Supplementary Information**

**Supplementary tables**

Supplementary Table 1. Antibody information
Supplementary Table 2. Connectivity Map analysis result

**Supplementary Figure Legends**

**Supplementary Fig. 1. Molecular docking of coxibs to human PROKR1**
Top view of the predicted protein shows valdecoxib (VAL, blue), rofecoxib (ROFE, red), paracoxib (PARE, yellow) bound to a binding pocket surrounded by the transmembrane domain (TM) of PROKR1. The boxes on the right show the binding pose of valdecoxib (top), rofecoxib (middle), and parecoxib (bottom), and the interacting residues of PROKR1 are represented by single letter amino acid symbols (F: phenylalanine, G: glycine, R: arginine, T: threonine).

**Supplementary Fig. 2. IP1 accumulation response in HEK293T cells**
IP1 accumulation is measured using human PROKR1-overexpressing (TG) or deficient (KO) HEK293T cells at different concentrations of each compound. Values are mean ± SD, n = 3.

**Supplementary Fig. 3. cAMP competition assay**
MTT assay is conducted using PROKR1-overexpressing (TG) or PROKR1-deficient (KO) HEK293T cells. cAMP accumulation is measured using human PROKR1-overexpressing cells at different concentrations of each compound. Values are mean ± SD, n = 3. $^{**}p < 0.01$, $^{***}p < 0.001$ vs. 0.78 μM, one-way ANOVA followed by Dunnett's post-hoc test. $EC_{99}$ is effective concentration 99.

**Supplementary Fig. 4. Coxib-induced oxidative muscle fiber specification**
**A**. Prokr1 signaling in C2C12 myotubes. Protein levels of Prokr1, phosphorylated Creb (pCreb), Nr4a2, Pgc1a, Tfam, and Myh7 are depicted. **B**. Immunocytochemistry of C2C12 myotubes. Green signals represent Myh7, blue for nuclei. Myh7 index is the Myh7-positive area divided by the nuclei count. Scale bar is 200 μm. **C**. Respiratory chain complex in C2C12 myotubes.

Protein levels of Ndufb8 (complex I: CI), Sdhb (CII), Uqcrc2 (CIII), Mtco1 (CIV), and Atp5a (CV) are depicted. **D**. Mitochondria mass in C2C12 myotubes. MitoTracker-positive area is divided by nuclei count. **E**. Fatty acid oxidation (FAO) activity in C2C12 myotubes. Y-axis indicates FAO activity (U/µg protein). Values are mean ± SD, n = 3. *$p < 0.05$, **$p < 0.01$, ***$p < 0.001$, ****$p < 0.0001$ vs. control group (CTRL), one-way ANOVA followed by Dunnett's post-hoc test (A, C, E) or Kruskal-Wallis test followed by Dunn's post-hoc test (B, D).

**Supplementary Fig. 5. Western blotting of Prokr1 signaling molecules in C2C12 myotubes**

Each protein symbol and MW were labelled on the left side of the panel. CTRL: vehicle control, PK2: prokineticin 2, DER: deracoxib, CEL: celecoxib.

**Supplementary Fig. 6. Quantification of Myh4 and Myh2 in C2C12 myotubes**

CTRL: vehicle control, PK2: prokineticin 2, DER: deracoxib, CEL: celecoxib. Values are mean ± SD, n = 3. *$p < 0.05$ vs. control group (CTRL), one-way ANOVA followed by Dunnett's post-hoc test.

**Supplementary Fig. 7. Immunocytochemistry of Myh4 and Myh2 in C2C12 myotubes**

Green signals represent Myh4 (top), Myh2 (bottom), and blue for nuclei. Myh index is the Myh-positive area divided by the nuclei count. Scale bar is 200 µm. CTRL: vehicle control, PK2: prokineticin 2, DER: deracoxib, CEL: celecoxib. Values are mean ± SD, n = 3. *$p < 0.05$, **$p < 0.01$ vs. control group (CTRL), one-way ANOVA followed by Dunnett's post-hoc test.

**Supplementary Fig. 8. Total nuclei number and fusion index for C2C12 myotubes**

Total nuclei number in a unit area, fusion index for Myh7-, Myh4-, and Myh2-positive myotubes are presented. CTRL: vehicle control, PK2: prokineticin 2, DER: deracoxib, CEL: celecoxib. Values are mean ± SD, n = 3. ****$p < 0.0001$ vs. control group (CTRL), one-way ANOVA followed by Dunnett's post-hoc test.

**Supplementary Fig. 9. Mitochondrial mass in C2C12 myotubes**

Red signals indicate mitochondria, green for F-actin, and blue for nuclei. Bar scale is 200 µm. CTRL: vehicle control, PK2: prokineticin 2, DER: deracoxib, CEL: celecoxib.

**Supplementary Fig. 10. Western blotting of PROKR1 signaling molecules in HSKMC myotubes**

Each protein symbol and MW were labelled on the left side of the panel. CTRL: vehicle control, PK2: prokineticin 2, DER: deracoxib, CEL: celecoxib.

**Supplementary Fig. 11. Quantification of MYH4 and MYH2 in HSKMC myotubes**

CTRL: vehicle control, PK2: prokineticin 2, DER: deracoxib, CEL: celecoxib. Values are mean ± SD, n = 3. *$p < 0.05$, **$p < 0.01$ vs. control group (CTRL), one-way ANOVA followed by Dunnett's post-hoc test.

**Supplementary Fig. 12. Immunocytochemistry of MYH4 and MYH2 in HSKMC myotubes**

Green signals represent MYH4 (top), MYH2 (bottom), and blue for nuclei. MYH index is the MYH-positive area divided by the nuclei count. Scale bar is 200 µm. CTRL: vehicle control, PK2: prokineticin 2, DER: deracoxib, CEL: celecoxib. Values are mean ± SD, n = 3. *$p < 0.05$ vs. control group (CTRL), one-way ANOVA followed by Dunnett's post-hoc test.

**Supplementary Fig. 13. Total nuclei number and fusion index for HSKMC myotubes**

Total nuclei number in a unit area, fusion index for MYH7-, MYH4-, and MYH2-positive myotubes are presented. CTRL: vehicle control, PK2: prokineticin 2, DER: deracoxib, CEL: celecoxib. Values are mean ± SD, n = 3. **$p < 0.01$, ***$p < 0.001$, ****$p < 0.0001$ vs. control group (CTRL), one-way ANOVA followed by Dunnett's post-hoc test.

**Supplementary Fig. 14. Mitochondrial mass in HSKMC myotubes**

Red signals indicate mitochondria, green for F-actin, and blue for nuclei. Bar scale is 200 µm. CTRL: vehicle control, PK2: prokineticin 2, DER: deracoxib, CEL: celecoxib.

**Supplementary Fig. 15. Attenuation of celecoxib-induced oxidative muscle fiber specification in *Prokr1* knock-downed mouse myotubes**

**A**. Attenuation of celecoxib-induced Prokr1 signaling activity. **B**. Attenuation of celecoxib-induced Myh7-postive myogenesis. Scale bar is 200 µm. CEL: celecoxib. Values are mean ± SD, n = 3. *$p < 0.05$, ***$p < 0.001$ vs. siRNA-scramble, one-way ANOVA followed by

Dunnett's post-hoc test.

**Supplementary Fig. 16. Body composition of neonatal mice**

Fat (red) and muscle distribution (blue) in mice by concentration using dual-energy X-ray absorptiometry (DEXA). The label at the top of each figure represents the concentration of celecoxib added in the diet. Scale bar is 3 cm.

**Supplementary Fig. 17. Relative muscle weights and muscle fiber counts of neonatal mice**

Bodyweight-relative gastrocnemius and soleus muscle weights (mg/g bw) and muscle fiber number per a unit area (0.44 $\mu m^2$) are presented. Values are mean ± SD, n = 3~4. *$p < 0.05$, **$p < 0.01$, ***$p < 0.001$ vs. 0 ppm, one-way ANOVA followed by Dunnett's post-hoc test.

**Supplementary Fig. 18. Macroscopic examination of thigh muscle**

Representative figures of different concentrations of celecoxib are presented. The concentration of celecoxib is indicated at the top of each figure (ppm). Scale bar is 1000 μm.

**Supplementary Fig. 19. H&E staining of neonate muscle tissues**

Representative figures of muscle tissue from male and female mice are shown. The concentration of celecoxib is indicated at the top of each figure. Scale bar is 100 μm.

**Supplementary Fig. 20. Western blotting of Prokr1 signal molecules in male neonate muscle tissues**

Each protein symbol and MW were labelled on the left side of the panel. The concentration of celecoxib is indicated at the top of each panel.

**Supplementary Fig. 21. Western blotting of Prokr1 signal molecules in female neonate muscle tissues**

Each protein symbol and MW were labelled on the left side of the panel. The concentration of celecoxib is indicated at the top of each panel.

**Supplementary Fig. 22. Bodyweight changes of young adult mice**

Each graph shows the weekly bodyweight change of male (left) and female mice (right) from 3 weeks old to 12 weeks old. Values are mean ± SD, n = 15. *$p < 0.05$, **$p < 0.01$, ***$p <$

0.001, ****$p < 0.0001$ vs. 0 ppm, one-way ANOVA followed by Dunnett's post-hoc test.

**Supplementary Fig. 23. Food consumption of young adult mice**

Each graph shows the daily food consumption of male (left) and female mice (right) from 3 weeks old to 12 weeks old. Values are mean ± SD, n = 15. *$p < 0.05$, **$p < 0.01$, ***$p < 0.001$, ****$p < 0.0001$ vs. 0 ppm, one-way ANOVA followed by Dunnett's post-hoc test.

**Supplementary Fig. 24. Body composition of young adult mice**

Fat (red) and muscle distribution (blue) in mice by concentration using dual-energy X-ray absorptiometry (DEXA). The label at the top of each figure represents the concentration of celecoxib added in the diet. Scale bar is 3 cm.

**Supplementary Fig. 25. Glucose tolerance of young adult mice**

Blood glucose levels during an oral glucose tolerance test (GTT) by concentration of celecoxib (ppm) are presented. Value is mean ± SD, n = 7. *$p < 0.05$ vs. 0 ppm, one-way ANOVA followed by Dunnett's post-hoc test.

**Supplementary Fig. 26. Insulin tolerance of young adult mice**

Blood glucose level during insulin tolerance test (ITT) by concentration of celecoxib (ppm) are presented. Value is mean ± SD, n = 7. *$p < 0.05$, **$p < 0.01$, ***$p < 0.001$, ****$p < 0.0001$ vs. 0 ppm, one-way ANOVA followed by Dunnett's post-hoc test.

**Supplementary Fig. 27. Area under the glucose curve during ITT and basal (fasting) glucose levels of young adult mice**

Area under the glucose concentration-time curve (AUC) during ITT and basal (fasting) glucose levels of young adult mice (12 weeks old) are presented. Value is mean ± SD, n = 3~4. *$p < 0.05$, **$p < 0.01$, ***$p < 0.001$, ****$p < 0.0001$ vs. 0 ppm, one-way ANOVA followed by Dunnett's post-hoc test.

**Supplementary Fig. 28. Respiratory exchange ratio of young adult mice**

The graph shows the respiratory exchange ratio (RER) by concentration of celecoxib (ppm) in male and female mice. White areas represent the light phase (8 am - 8 pm) and shaded areas for the dark phase (8 pm - 8 am).

**Supplementary Fig. 29. Energy expenditure of young adult mice**

Energy expenditure (EE) by concentration of celecoxib (ppm) in male and female mice are presented. White areas represent the light phase (8 am - 8 pm) and shaded areas for the dark phase (8 pm - 8 am).

**Supplementary Fig. 30. Organ weights of young adult mice**

**A**. Body weight-related organ weights. **B**. Absolute organ weights. White bar indicates 0 ppm of celecoxib, green for 300 ppm, blue for 700 ppm, and red for 1500 ppm. Values are mean ± SD, n = 4. *$p < 0.05$, **$p < 0.01$ vs. 0 ppm, one-way ANOVA followed by Dunnett's post-hoc test. Kruskal-Wallis test followed by Dunn's post-hoc text for male gastrocnemius muscle tissue, female soleus muscle tissue and liver weights, and one-way ANOVA followed by Dunnett's post-hoc test for others.

**Supplementary Fig. 31. H&E staining of young adult mice muscle tissues**

Representative figures of muscle tissue from male and female mice are shown. Animal sex, celecoxib concentration (ppm), and tissue are labeled in each figure. Scale bar is 100 μm. M: male, F: female, GAS: gastrocnemius muscle tissue, SOL: soleus muscle tissue.

**Supplementary Fig. 32. Mitochondria mass in young adult mice muscle tissues**

Histology shows the mitochondria (red) and nuclei (blue) of muscle fibers. The concentration of celecoxib is indicated at the top of each figure (ppm). Scale bar is 100 μm.

**Supplementary Fig. 33. Lipid content in young adult mice muscle tissues**

Qualitative and quantitative perilipin 2 (Plin2) expression levels in gastrocnemius muscle tissue of male and female young adult mice are presented. Green signal represents Plin2, blue for nuclei. Scale bar is 100 μm. M: male, F: female. Values are mean ± SD, n = 3. *$p < 0.05$ vs. 0 ppm, one-way ANOVA followed by Dunnett's post-hoc test

**Supplementary Fig. 34. Western blotting of Prokr1 signaling molecules in male young adult mice muscle tissues**

Each protein symbol and MW were labelled on the left side of the panel. The concentration of celecoxib is indicated at the top of each panel.

**Supplementary Fig. 35. Western blotting of Prokr1 signaling molecules in female young adult mice muscle tissues**

Each protein symbol and MW were labelled on the left side of the panel. The concentration of celecoxib is indicated at the top of each panel.

**Supplementary Fig. 36. Bodyweight changes of adult mice**

Each graph shows the weekly bodyweight change of male (left) and female mice (right) from 3 weeks old to 20 weeks old. Values are mean ± SD, n = 15. $*p < 0.05$, $**p < 0.01$, $***p < 0.001$, $****p < 0.0001$ vs. 0 ppm, Kruskal-Wallis test followed by Dunn's post-hoc text for male age (6, 7, 12, 13, 14, 15, 16, 17, 18, 19, 20 weeks) and one-way ANOVA followed by Dunnett's post-hoc test for others.

**Supplementary Fig. 37. Food consumption of adult mice**

Each graph shows the daily food consumption of male (left) and female mice (right) from 3 weeks old to 20 weeks old. Values are mean ± SD, n = 15. $*p < 0.05$, $**p < 0.01$, $***p < 0.001$, $****p < 0.0001$ vs. 0 ppm, Kruskal-Wallis test followed by Dunn's post-hoc text for male age (13,16, 17, 19, 20 weeks) and one-way ANOVA followed by Dunnett's post-hoc test for others.

**Supplementary Fig. 38. Body composition of adult mice**

Fat (red) and muscle distribution (blue) in mice by concentration using dual-energy X-ray absorptiometry (DEXA). The label at the top of each figure represents the concentration of celecoxib added in the diet. Scale bar is 2 cm.

**Supplementary Fig. 39. Glucose tolerance of adult mice**

Blood glucose levels during an oral glucose tolerance test (OGTT) by concentration of celecoxib (ppm) are presented. Value is mean ± SD, n = 7. $*p < 0.05$ vs. 0 ppm, one-way ANOVA followed by Dunnett's post-hoc test.

**Supplementary Fig. 40. Insulin tolerance of adult mice**

Blood glucose level during insulin tolerance test (UTT) by concentration of celecoxib (ppm) are presented. Value is mean ± SD, n = 7. $*p < 0.05$, $**p < 0.01$, $***p < 0.001$, $****p < 0.0001$ vs. 0 ppm, one-way ANOVA followed by Dunnett's post-hoc test.

**Supplementary Fig. 41. Area under the glucose curve during ITT and basal (fasting) glucose levels of adult mice**

Area under the glucose concentration-time curve (AUC) during ITT and basal (fasting) glucose levels of adult mice (20 weeks old) are presented. Value is mean ± SD, n = 3~4. *$p < 0.05$, **$p < 0.01$, ***$p < 0.001$, ****$p < 0.0001$ vs. 0 ppm, one-way ANOVA followed by Dunnett's post-hoc test.

**Supplementary Fig. 42. Respiratory exchange ratio of adult mice**

The graph shows the respiratory exchange ratio (RER) by concentration of celecoxib (ppm) in male and female mice. White areas represent the light phase (8 am – 8 pm) and shaded areas for the dark phase (8 pm – 8 am).

**Supplementary Fig. 43. Energy expenditure of adult mice**

Energy expenditure (EE) by concentration of celecoxib (ppm) in male and female mice are presented. White areas represent the light phase (8 am – 8 pm) and shaded areas for the dark phase (8 pm – 8 am).

**Supplementary Fig. 44. Organ weights of adult mice**

**A**. Body weight-related organ weights. **B**. Absolute organ weights. White bar indicates 0 ppm of celecoxib, green for 300 ppm, blue for 700 ppm, and red for 1500 ppm. Values are mean ± SD, n = 3. *$p < 0.05$, **$p < 0.01$ vs. 0 ppm, one-way ANOVA followed by Dunnett's post-hoc test. one-way ANOVA followed by Dunnett's post-hoc test.

**Supplementary Fig. 45. H&E staining of adult mice muscle tissues**

Representative figures of muscle tissue from male and female mice are shown. Animal sex, celecoxib concentration (ppm), and tissue are labeled in each figure. Scale bar is 100 μm. M: male, F: female, GAS: gastrocnemius muscle tissue, SOL: soleus muscle tissue.

**Supplementary Fig. 46. Mitochondria mass in adult mice muscle tissues**

Histology shows the mitochondria (red) and nuclei (blue) of muscle fibers. The concentration of celecoxib is indicated at the top of each figure (ppm). Scale bar is 100 μm.

**Supplementary Fig. 47. Lipid content in adult mice muscle tissues**

Qualitative and quantitative perilipin 2 (PLIN2) expression levels in gastrocnemius muscle tissue of male and female adult mice are presented. Green signal represents PLIN2, blue for nuclei. Scale bar is 100 µm. M: male, F: female. Values are mean ± SD, n = 3. *$p < 0.05$ vs. 0 ppm, one-way ANOVA followed by Dunnett's post-hoc test Values are mean ± SD, n = 3. **$p < 0.01$, ***$p < 0.001$ vs. 0 ppm, one-way ANOVA followed by Dunnett's post-hoc test

**Supplementary Fig. 48. Western blotting of Prokr1 signaling molecules in male adult mice muscle tissues**

Each protein symbol and MW were labelled on the left side of the panel. The concentration of celecoxib is indicated at the top of each panel.

**Supplementary Fig. 49. Western blotting of Prokr1 signaling molecules in female adult mice muscle tissues**

Each protein symbol and MW were labelled on the left side of the panel. The concentration of celecoxib is indicated at the top of each panel.

**Supplementary Fig. 1**

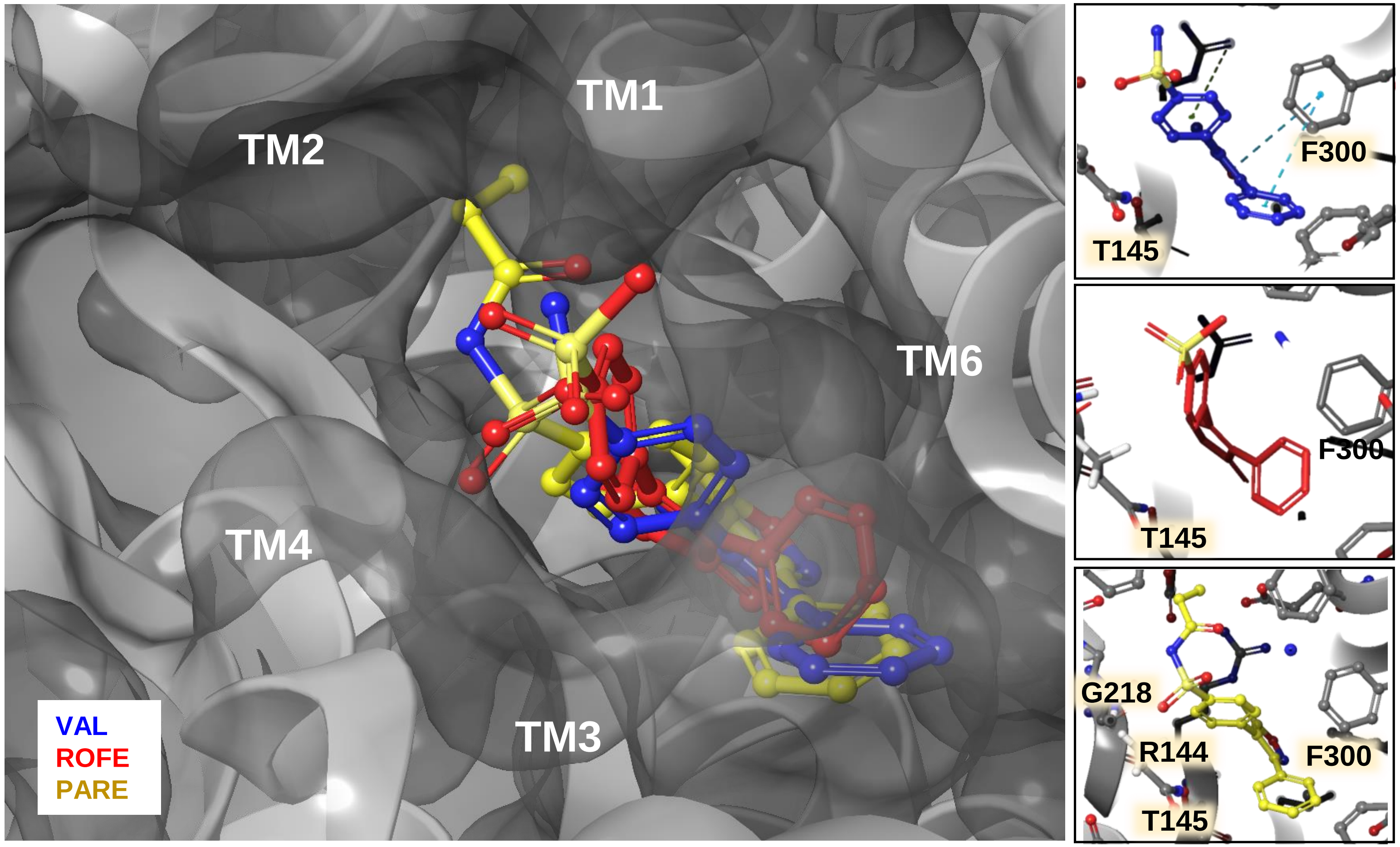

# Supplementary Fig. 2

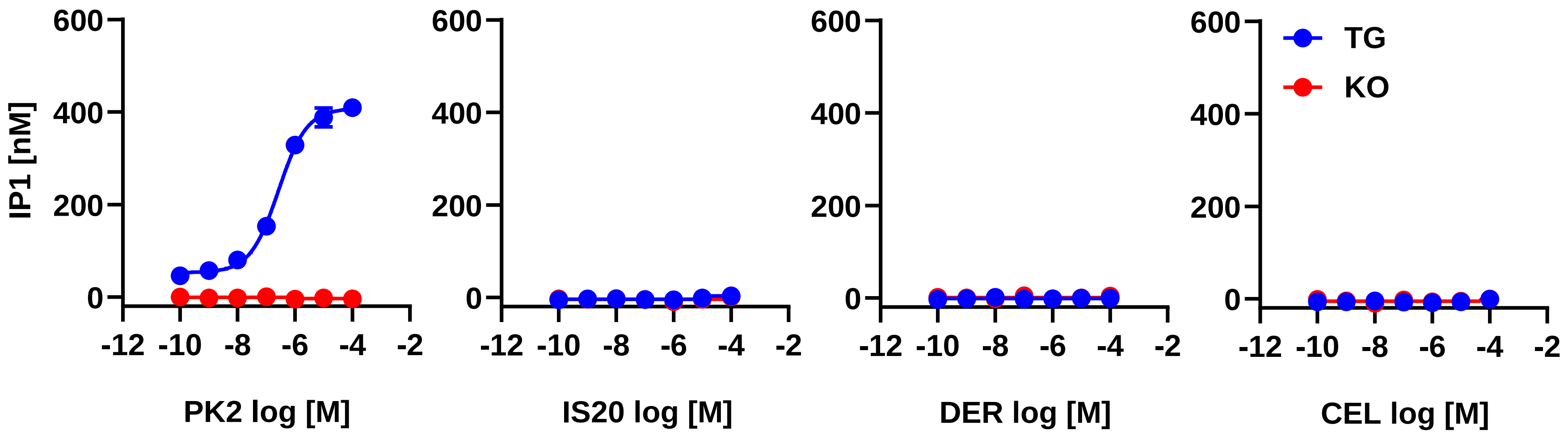

## Supplementary Fig. 3

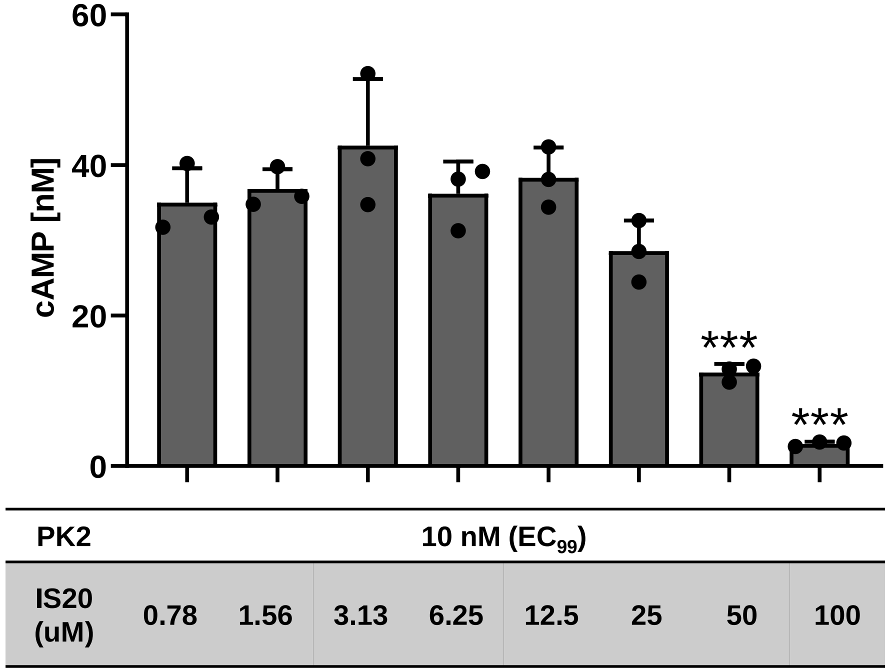


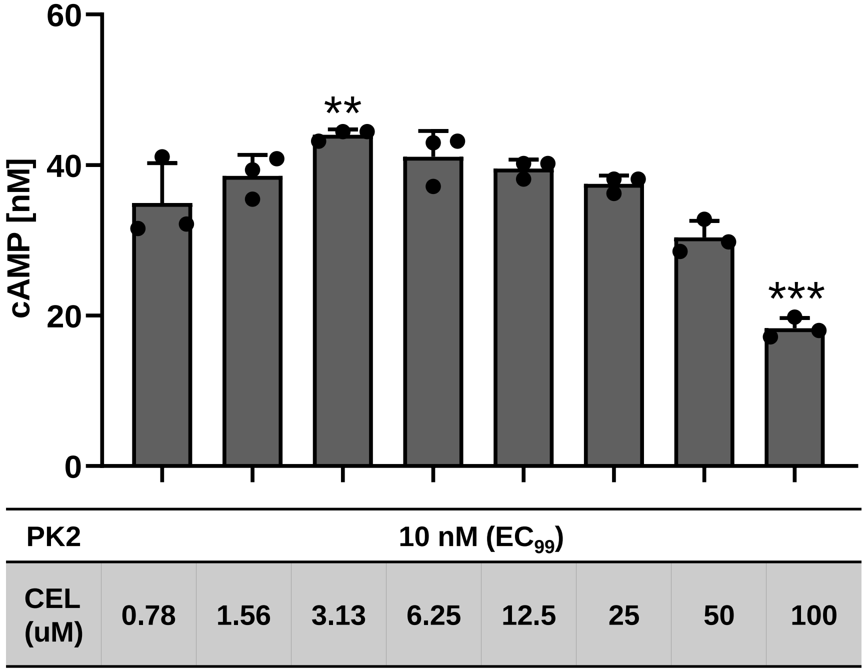

## Supplementary Fig. 4

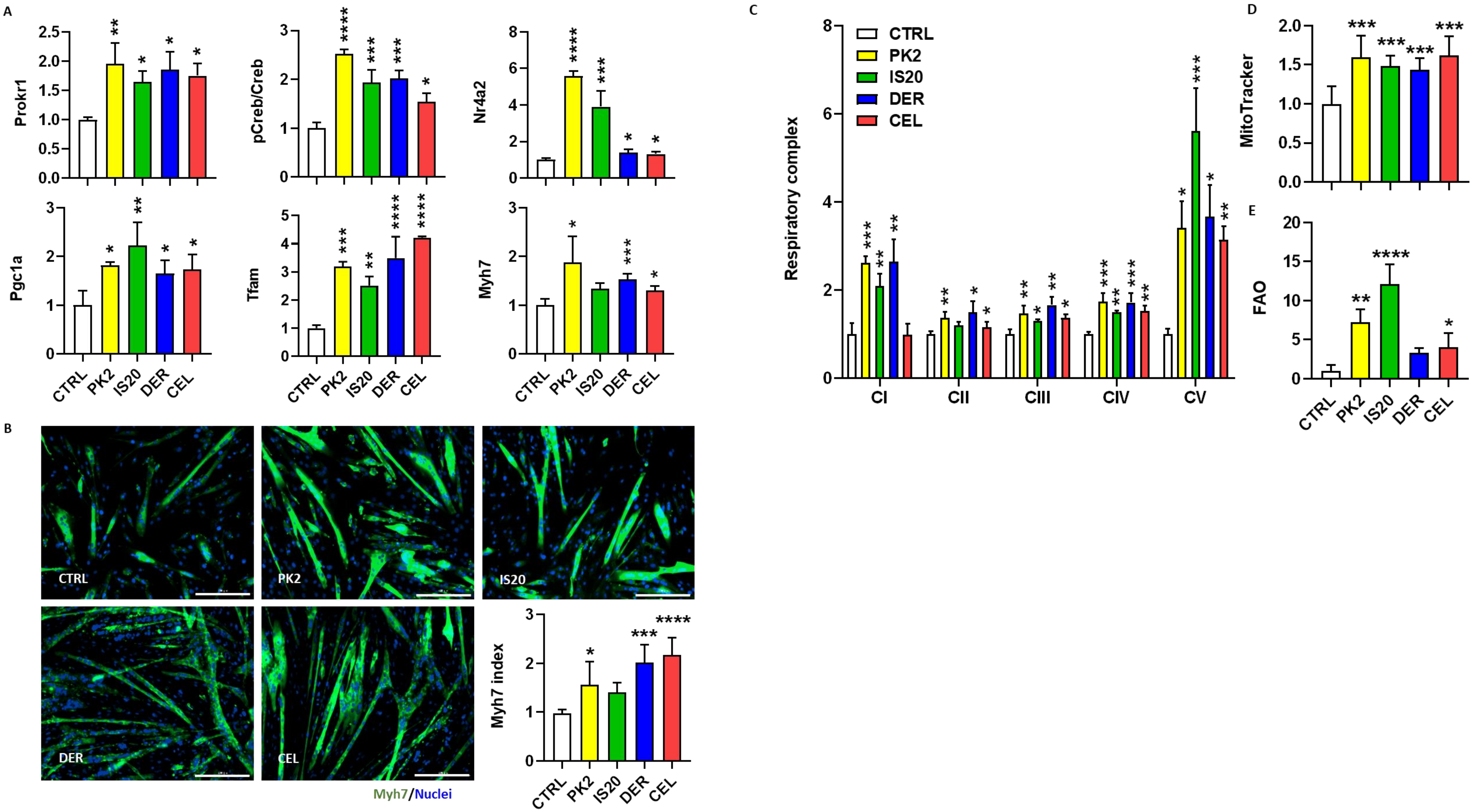

# Supplementary Fig. 5

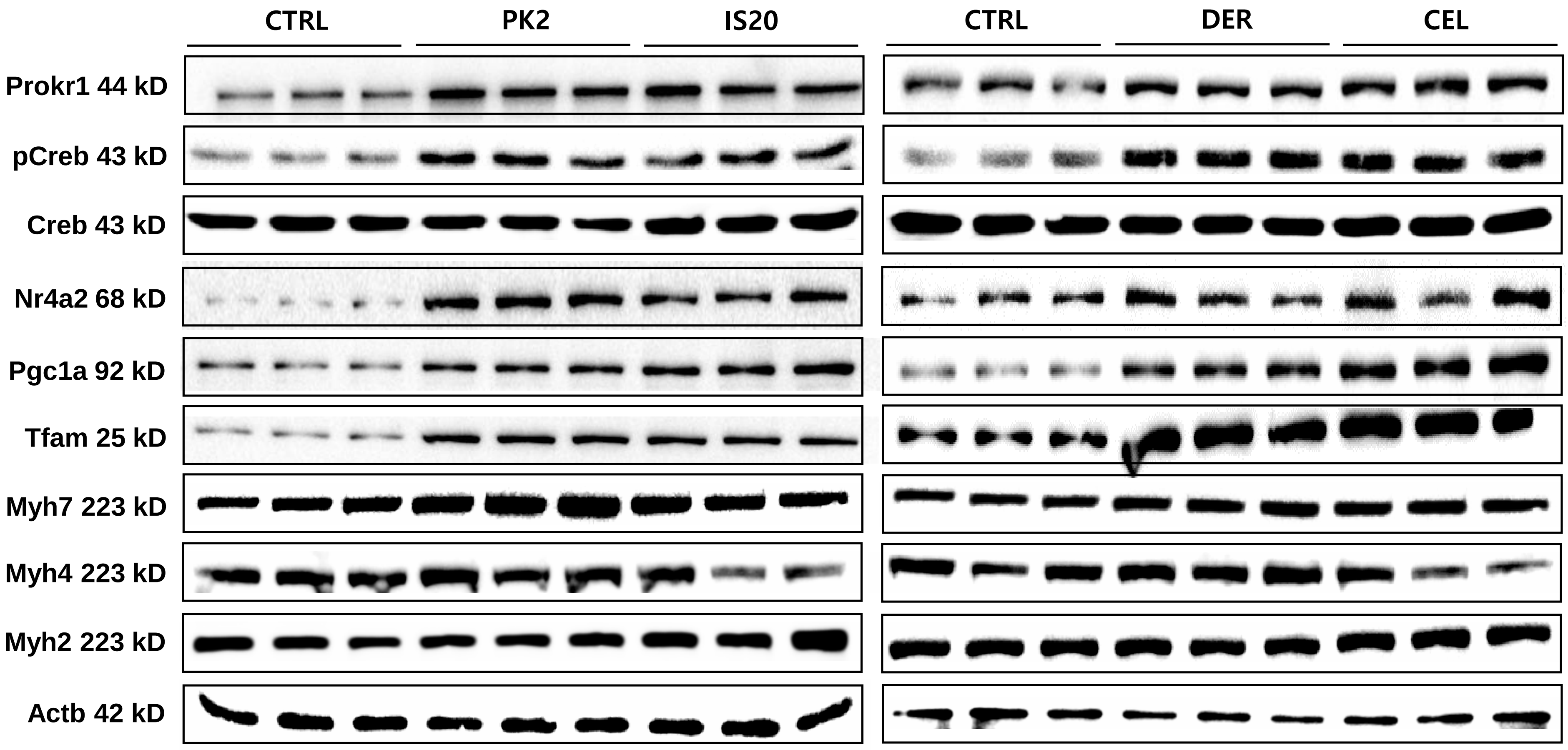

**Supplementary Fig. 6**

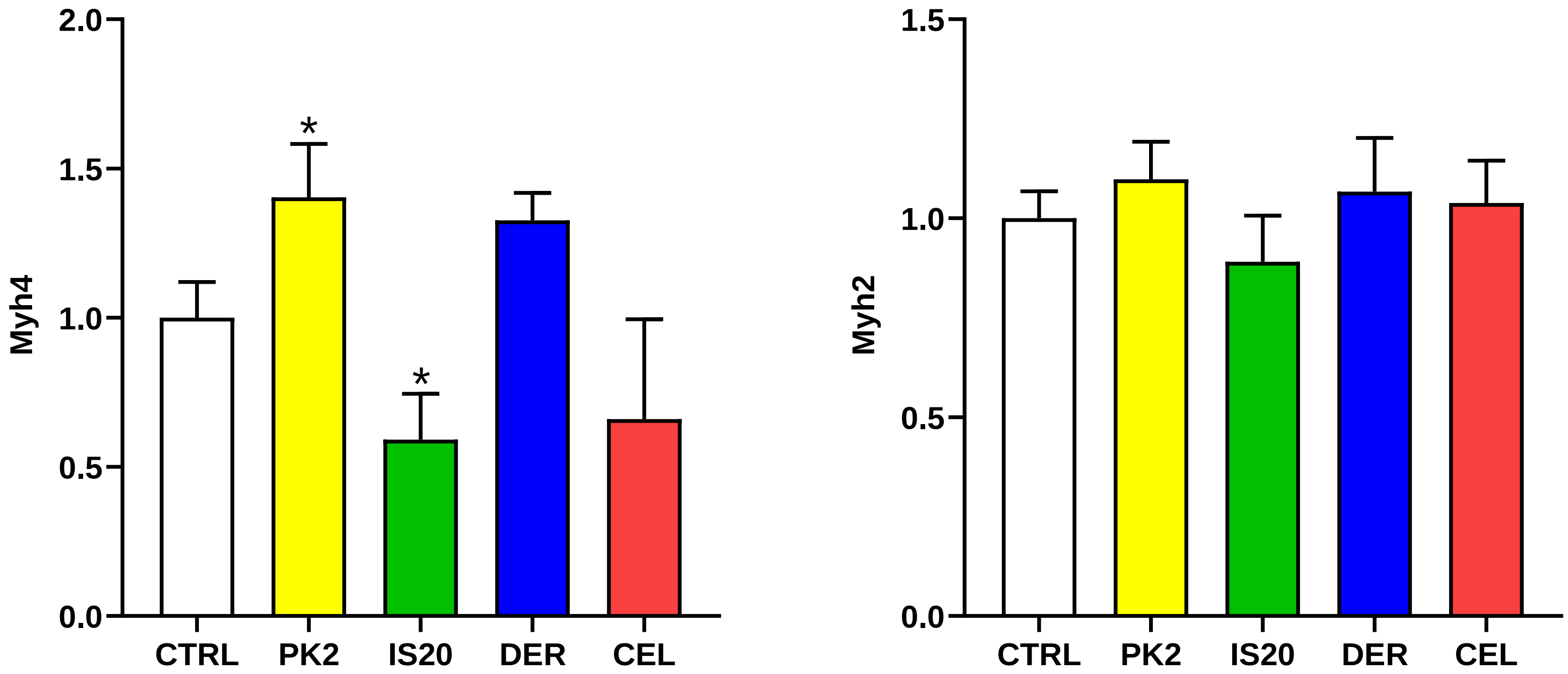

Supplementary Fig. 7

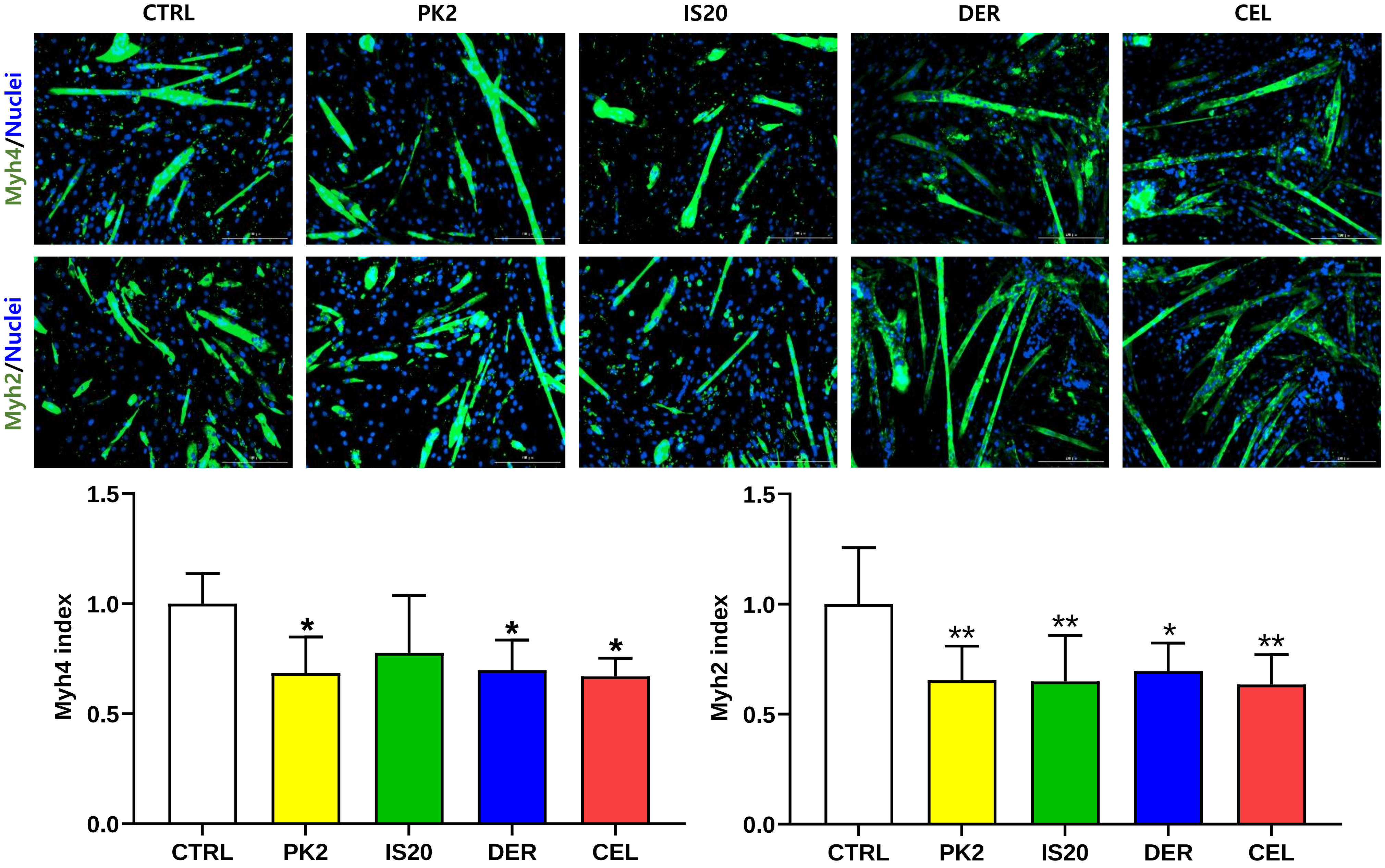

# Supplementary Fig. 8

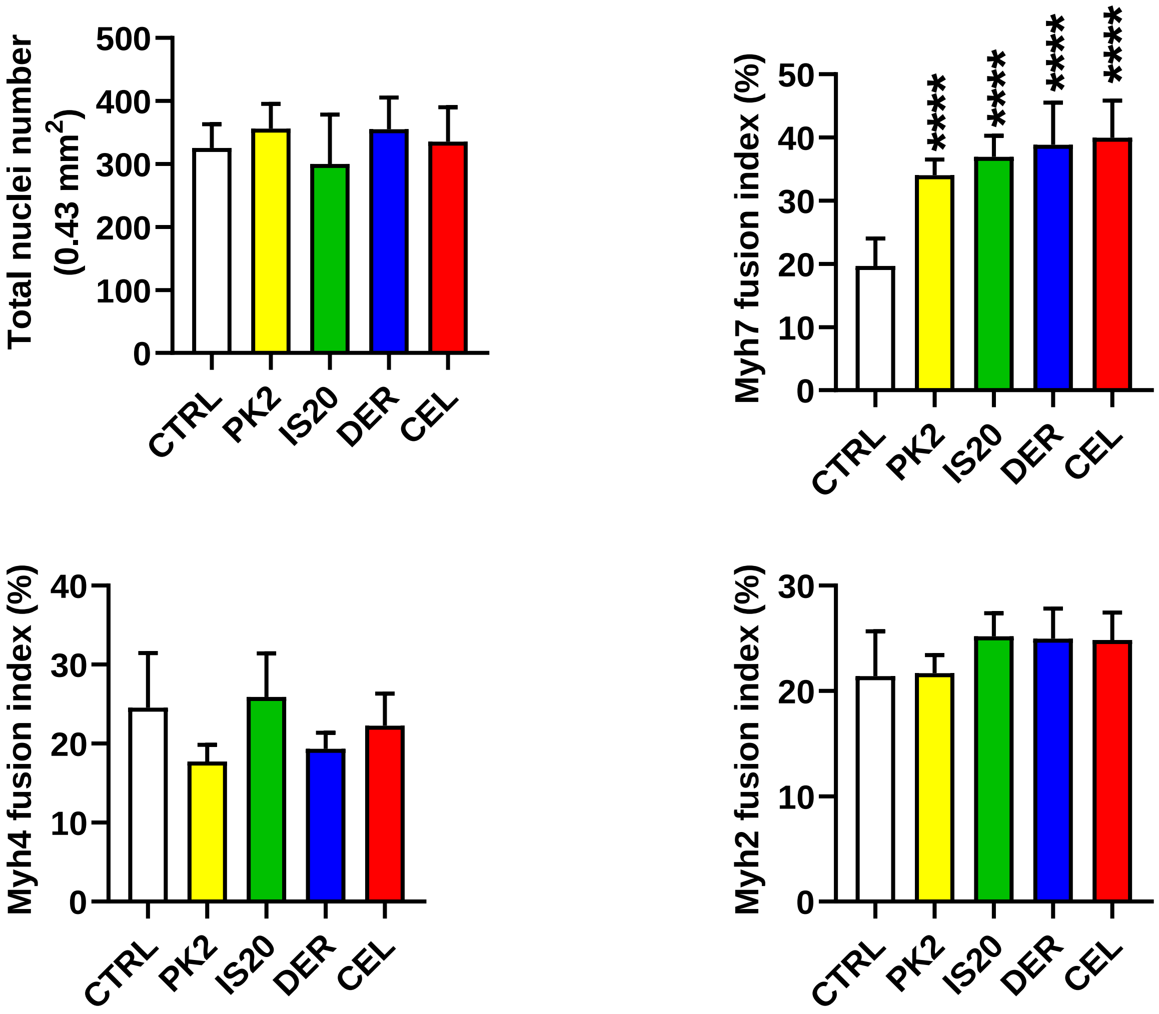

# Supplementary Fig. 9

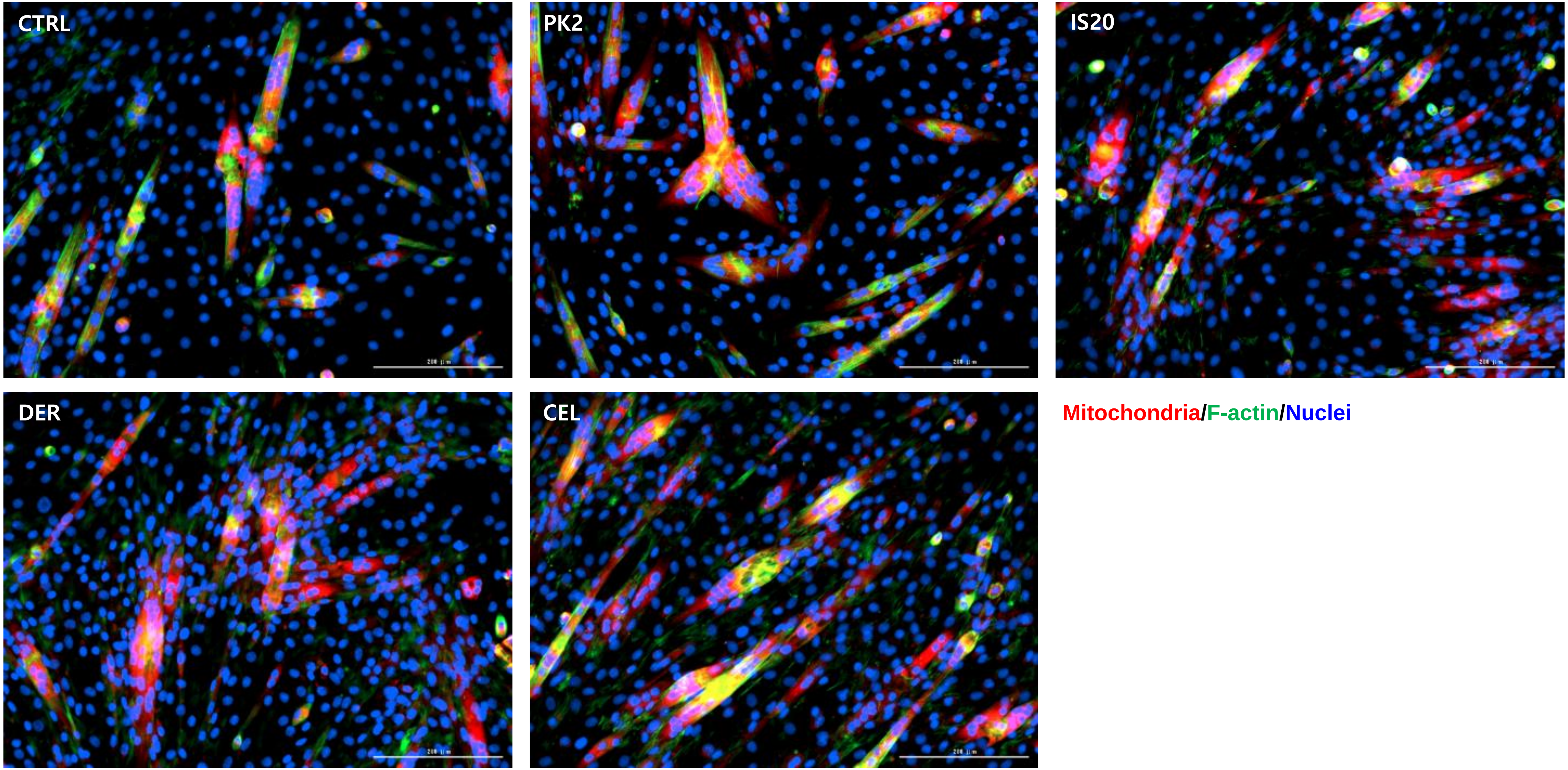

# Supplementary Fig. 10

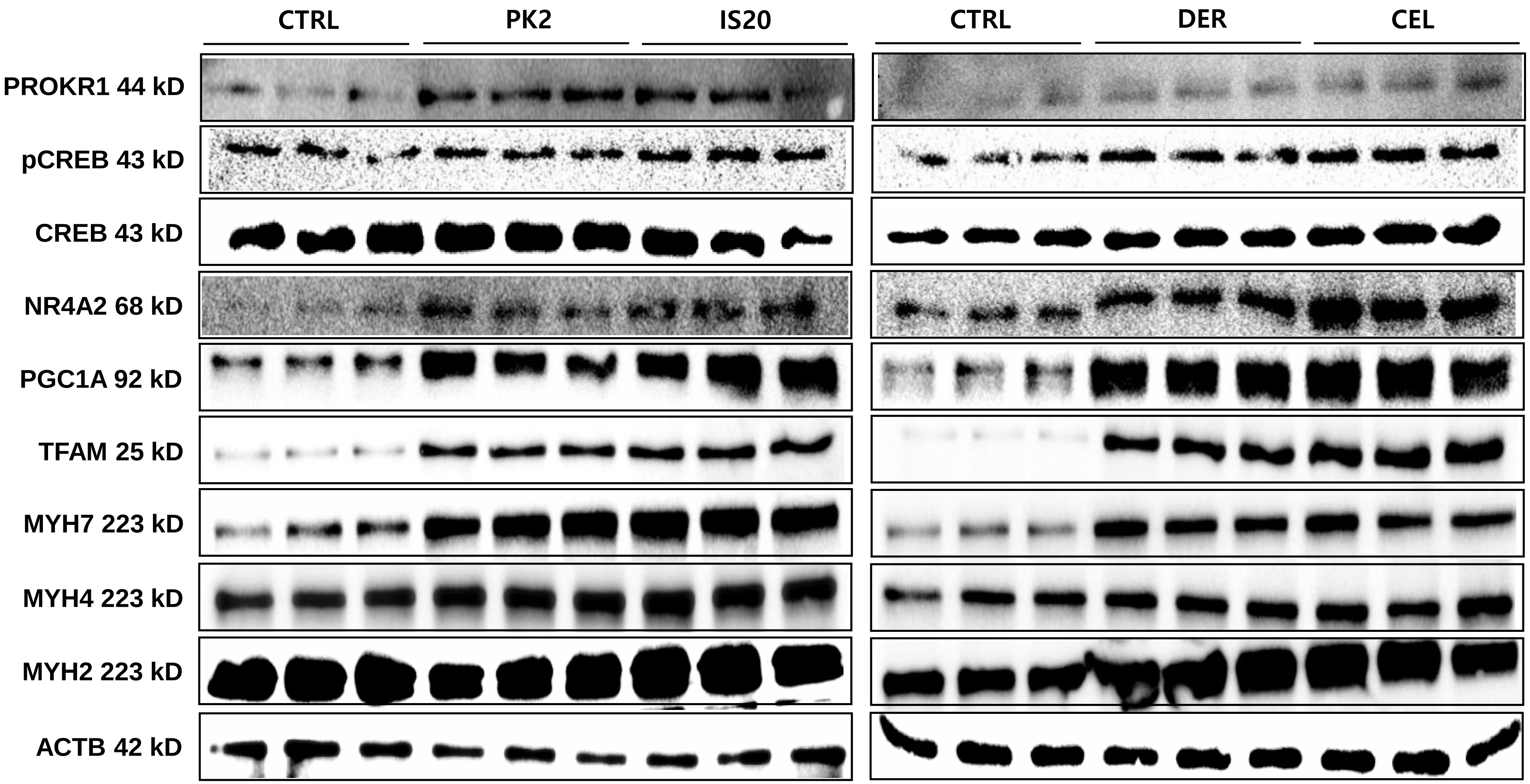

**Supplementary Fig. 11**

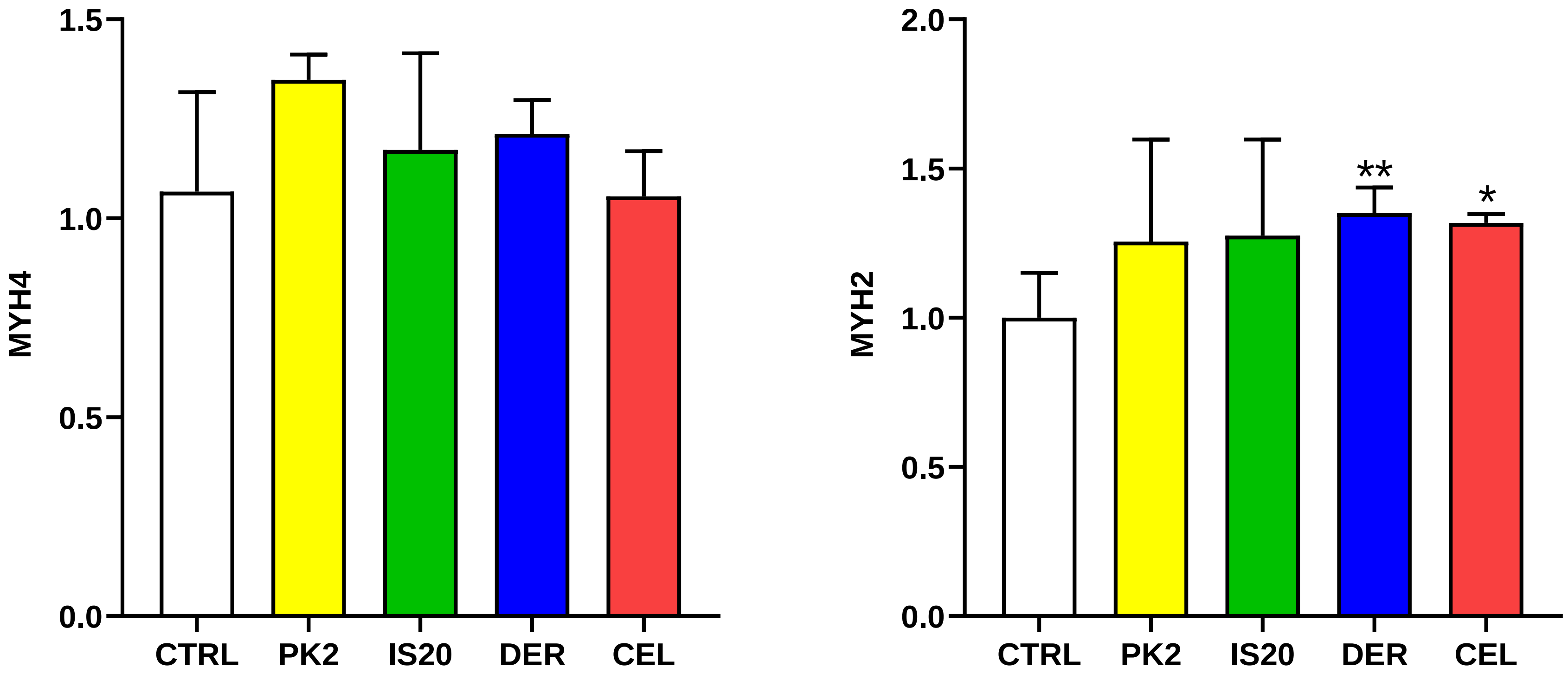

# Supplementary Fig. 12

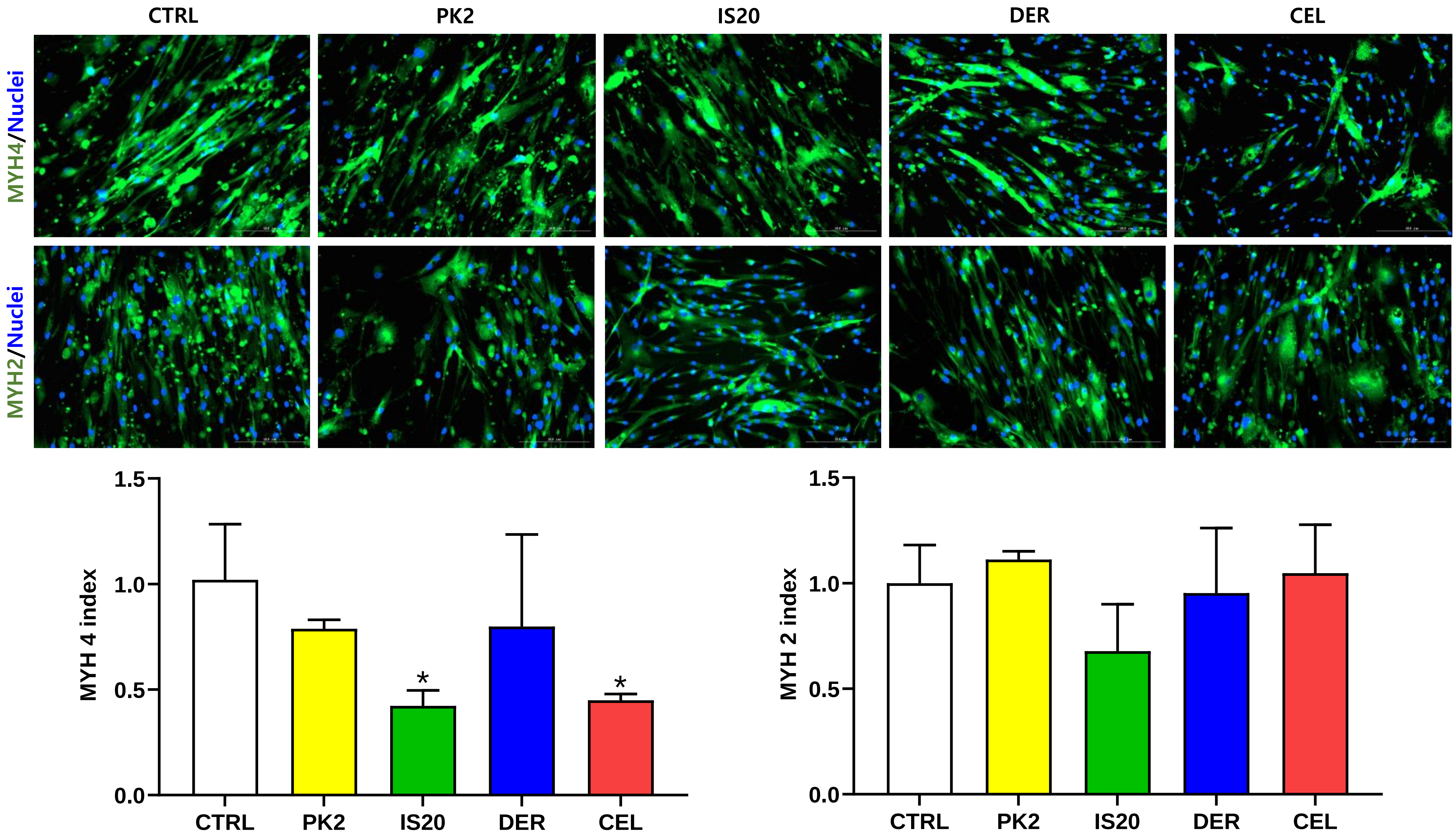

# Supplementary Fig. 13

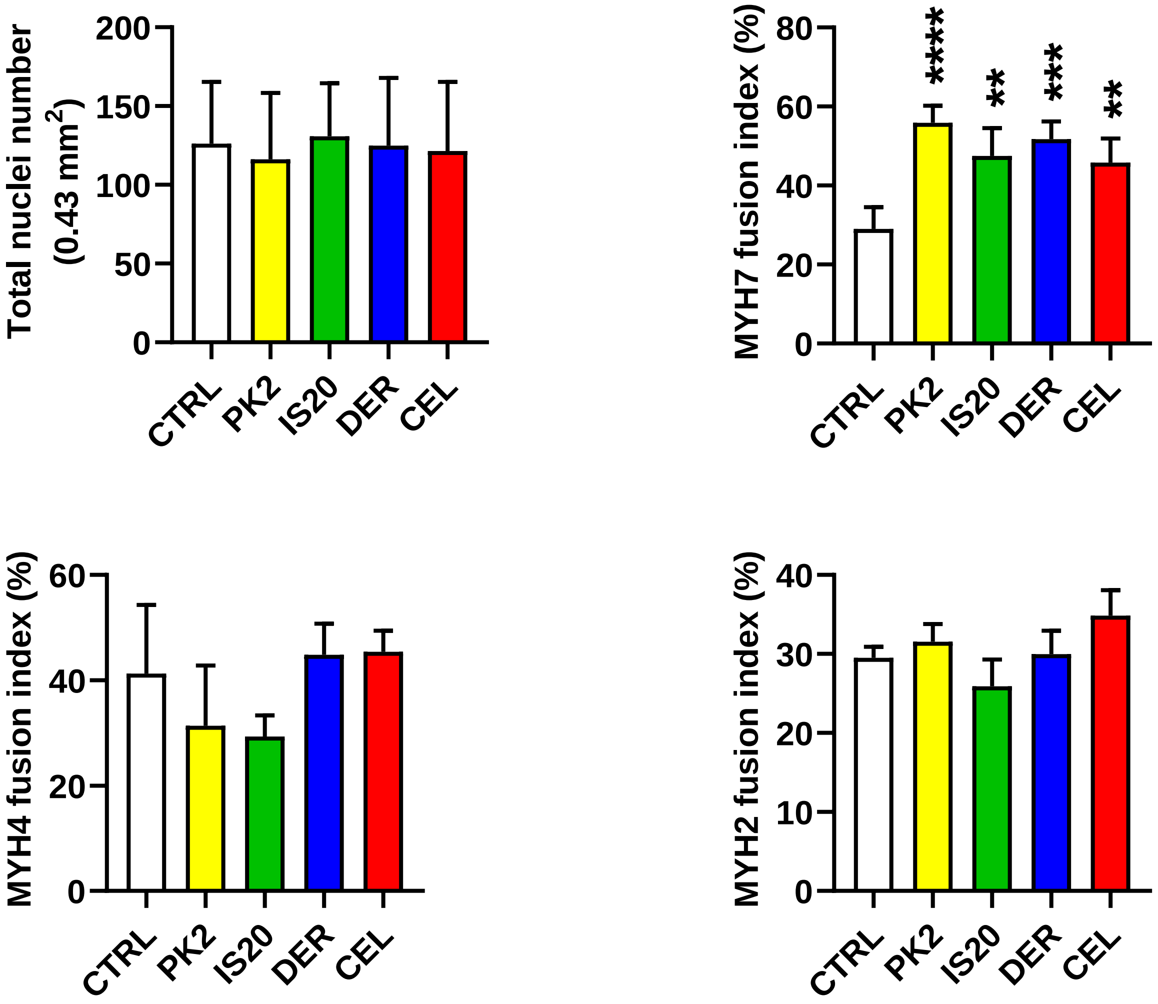

# Supplementary Fig. 14

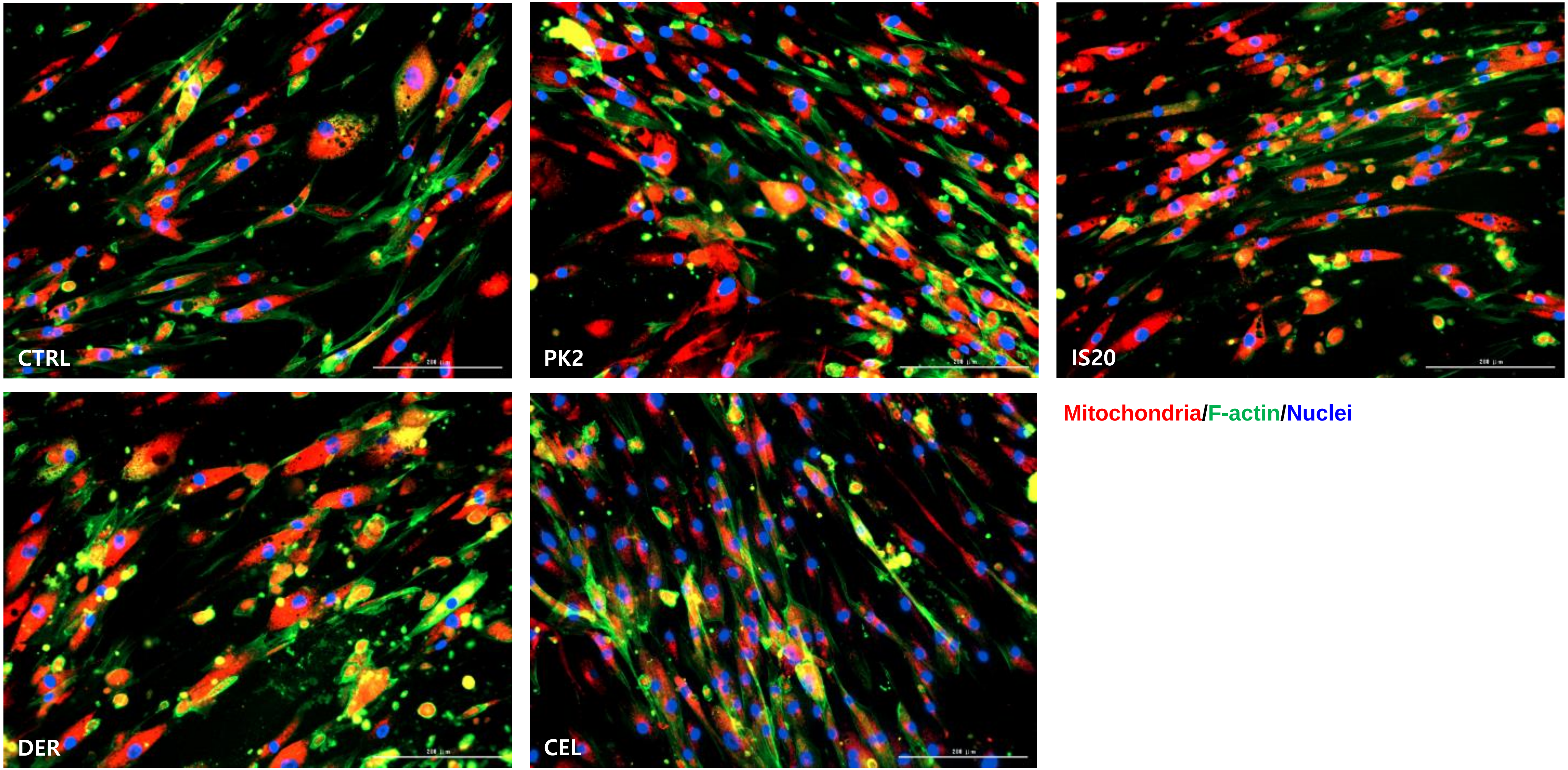

# Supplementary Fig. 15

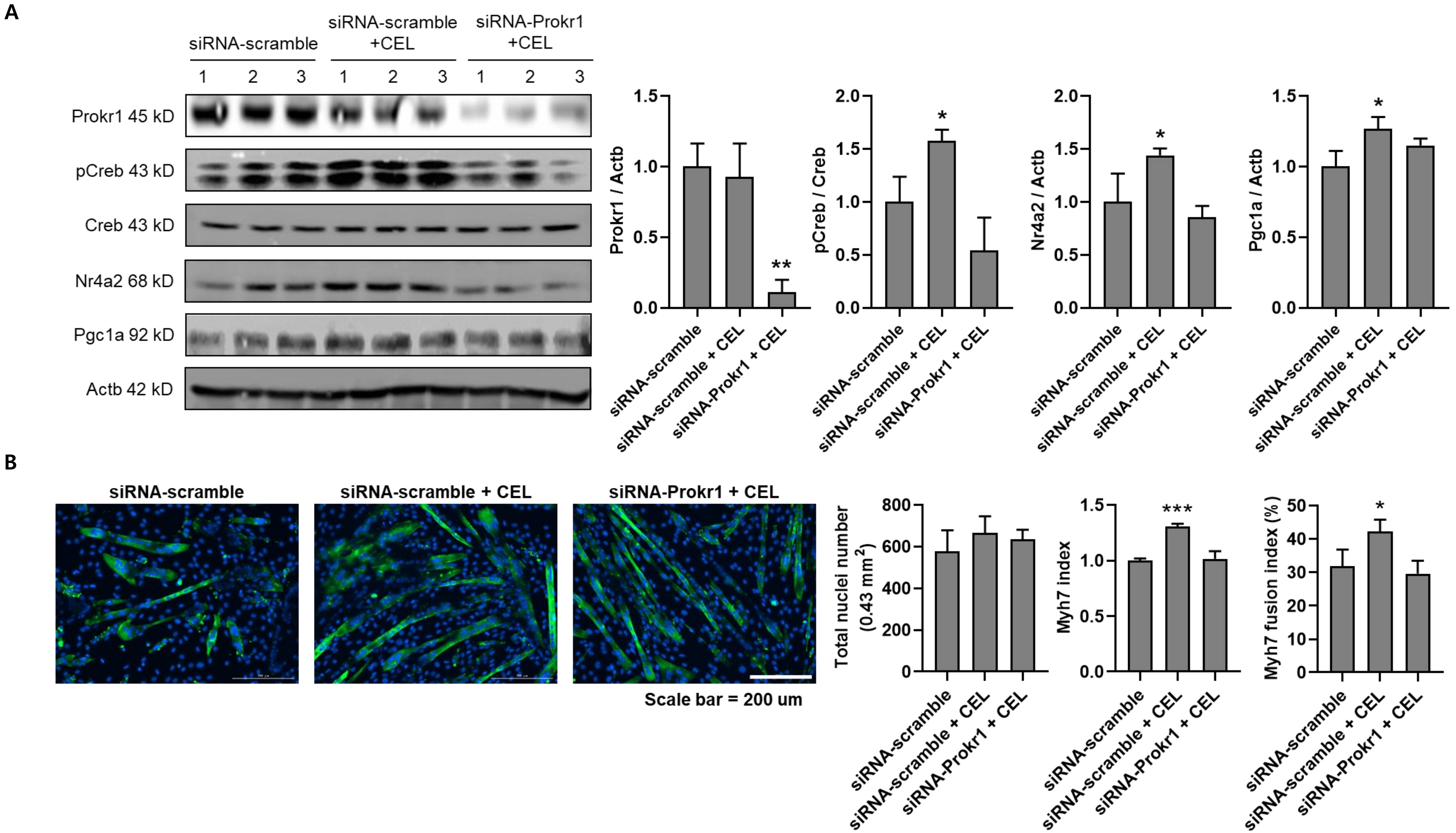

# Supplementary Fig. 16

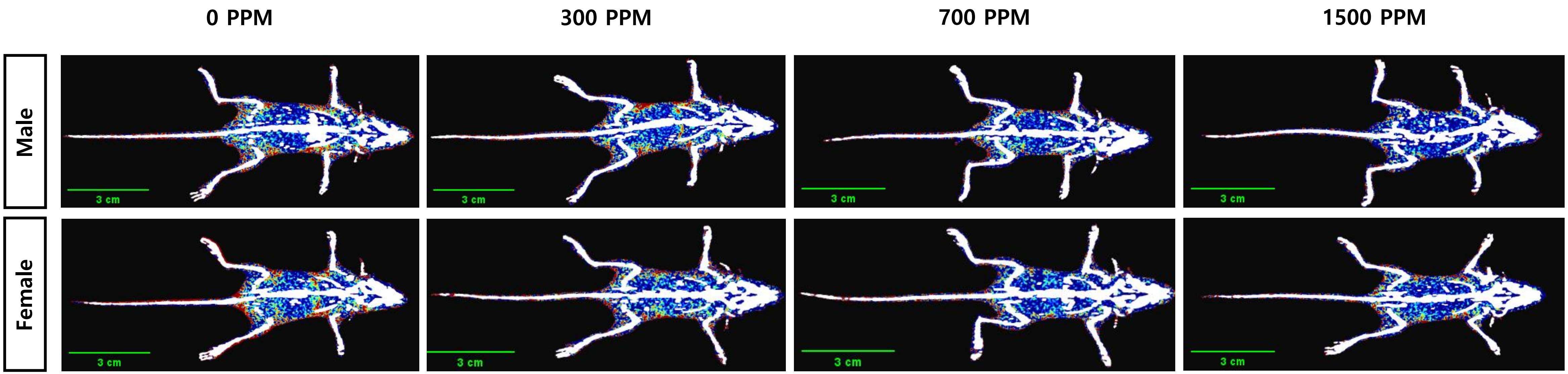

# Supplementary Fig. 17

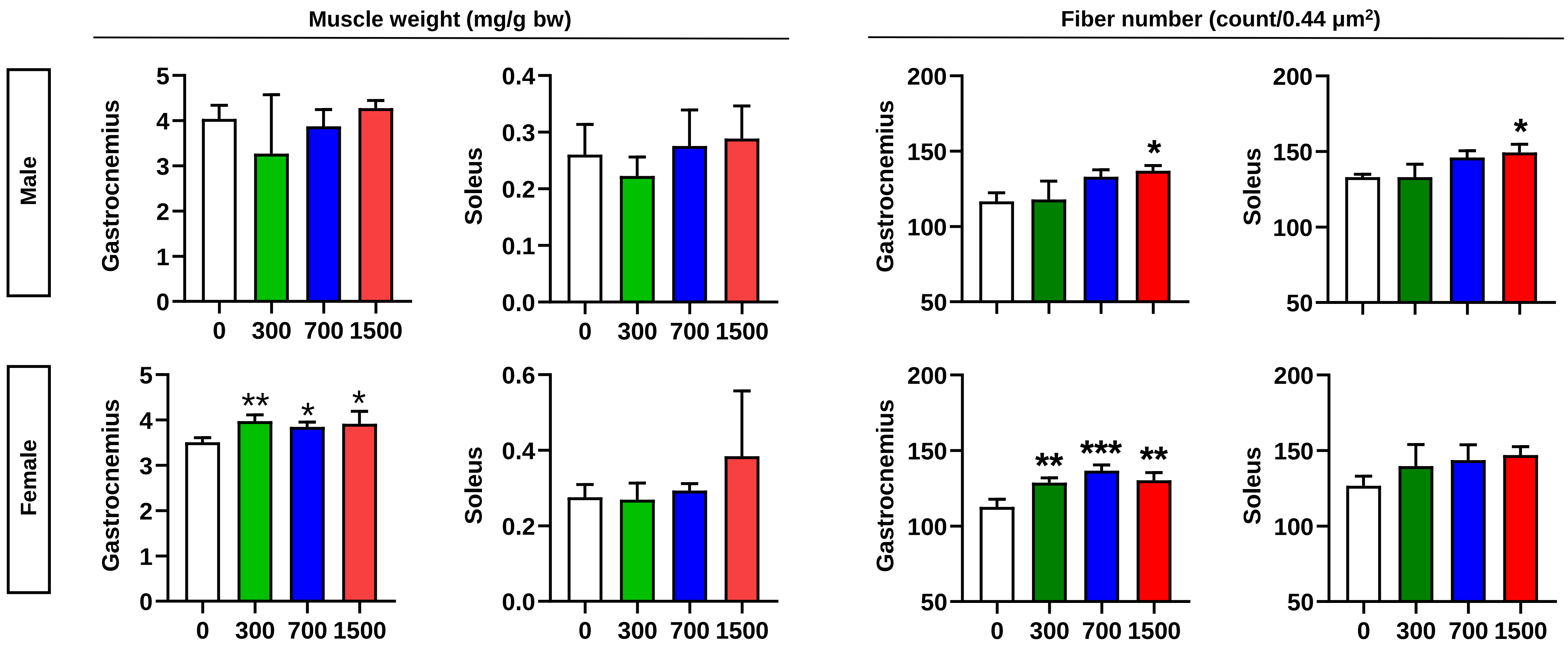

## Supplementary Fig. 18

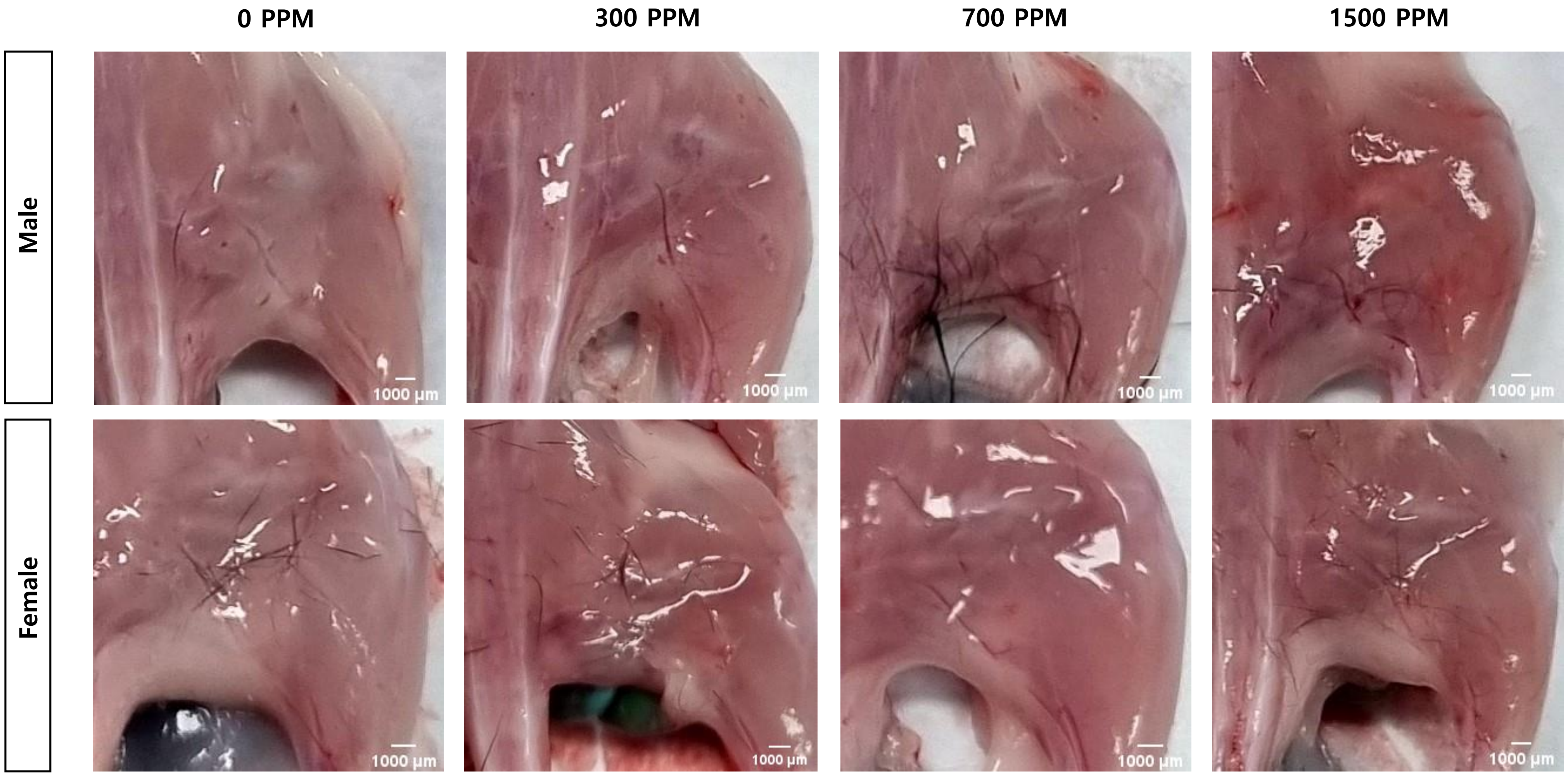

# Supplementary Fig. 19

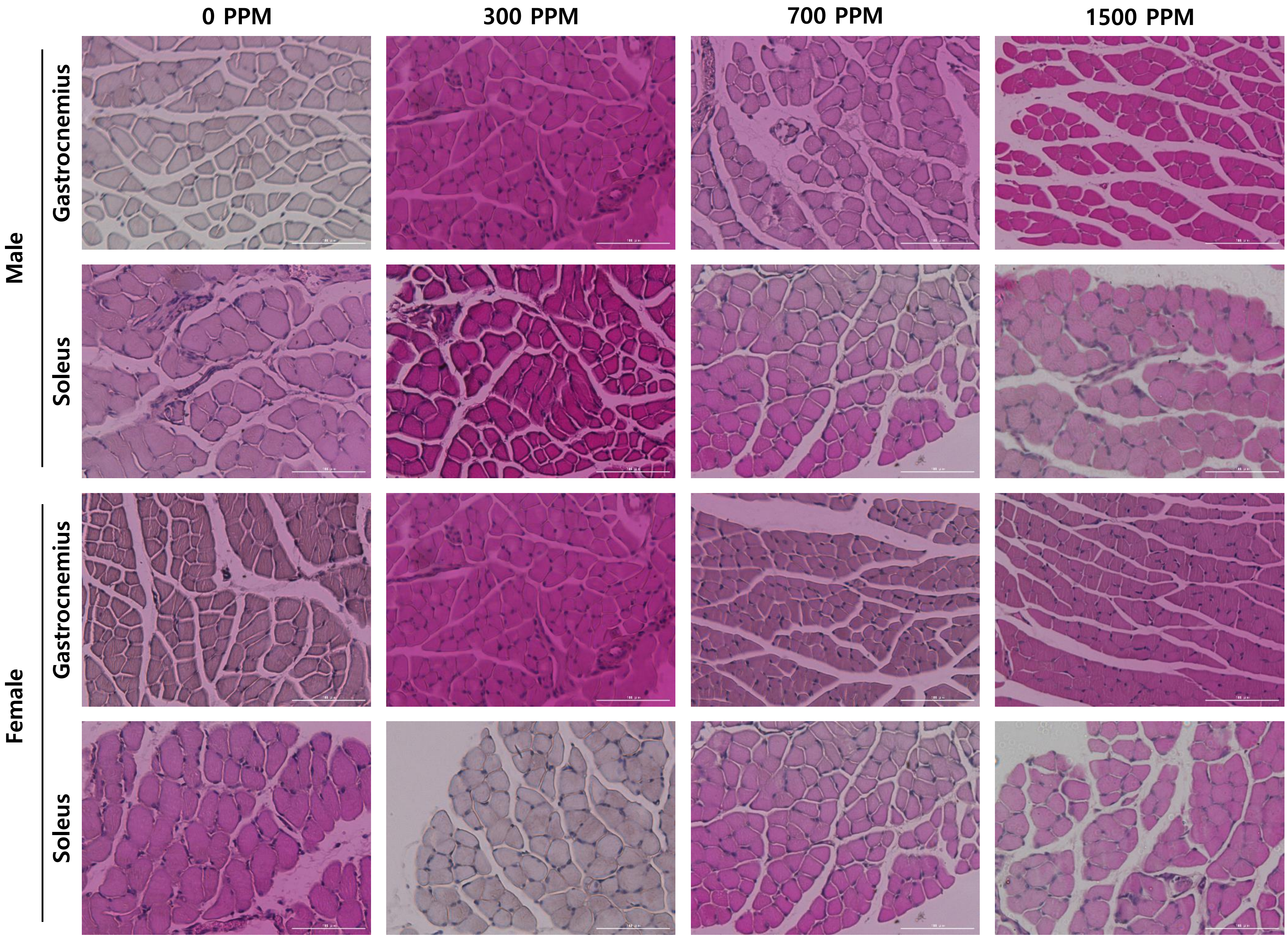

# Supplementary Fig. 20

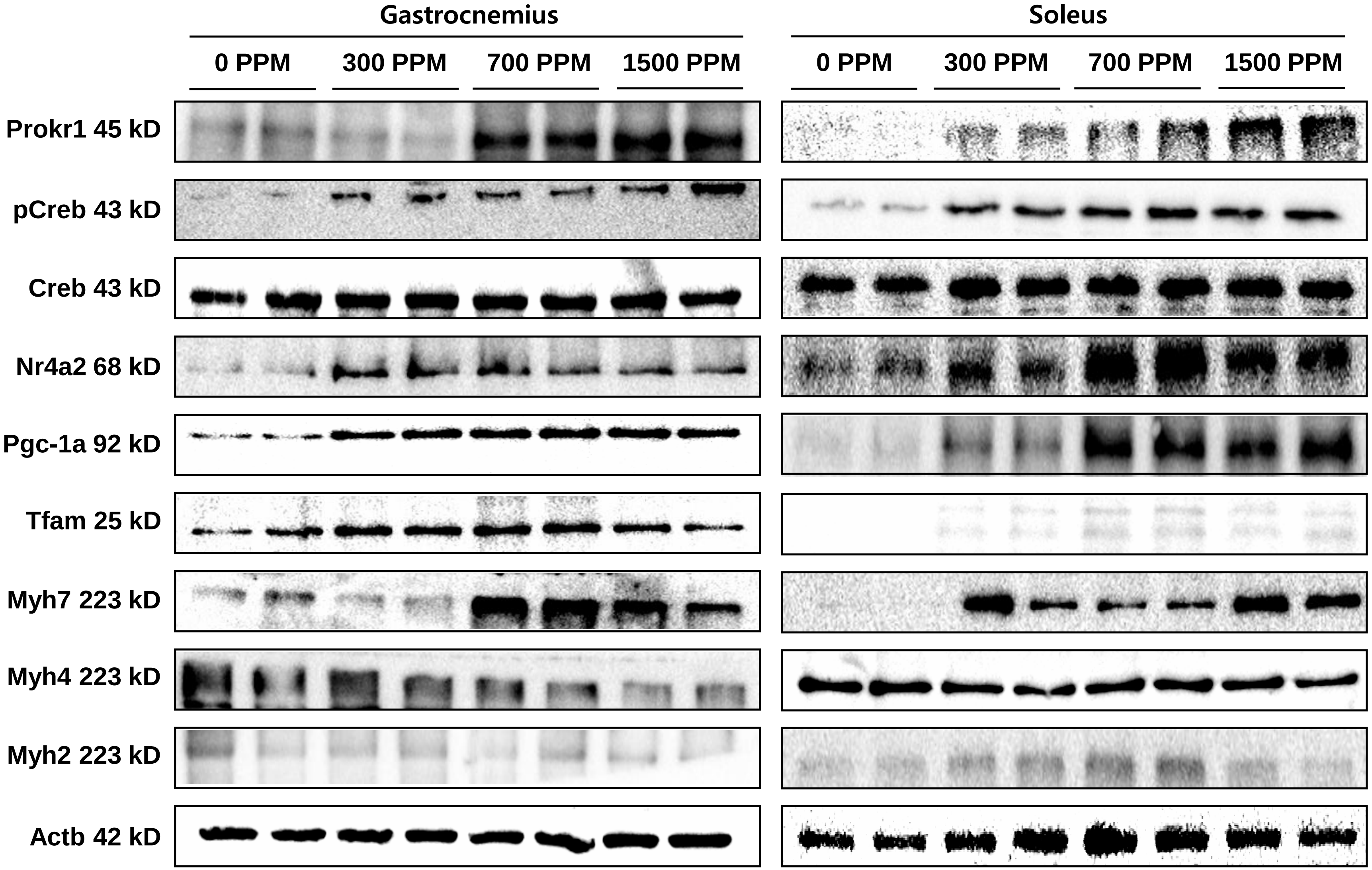

## Supplementary Fig. 21

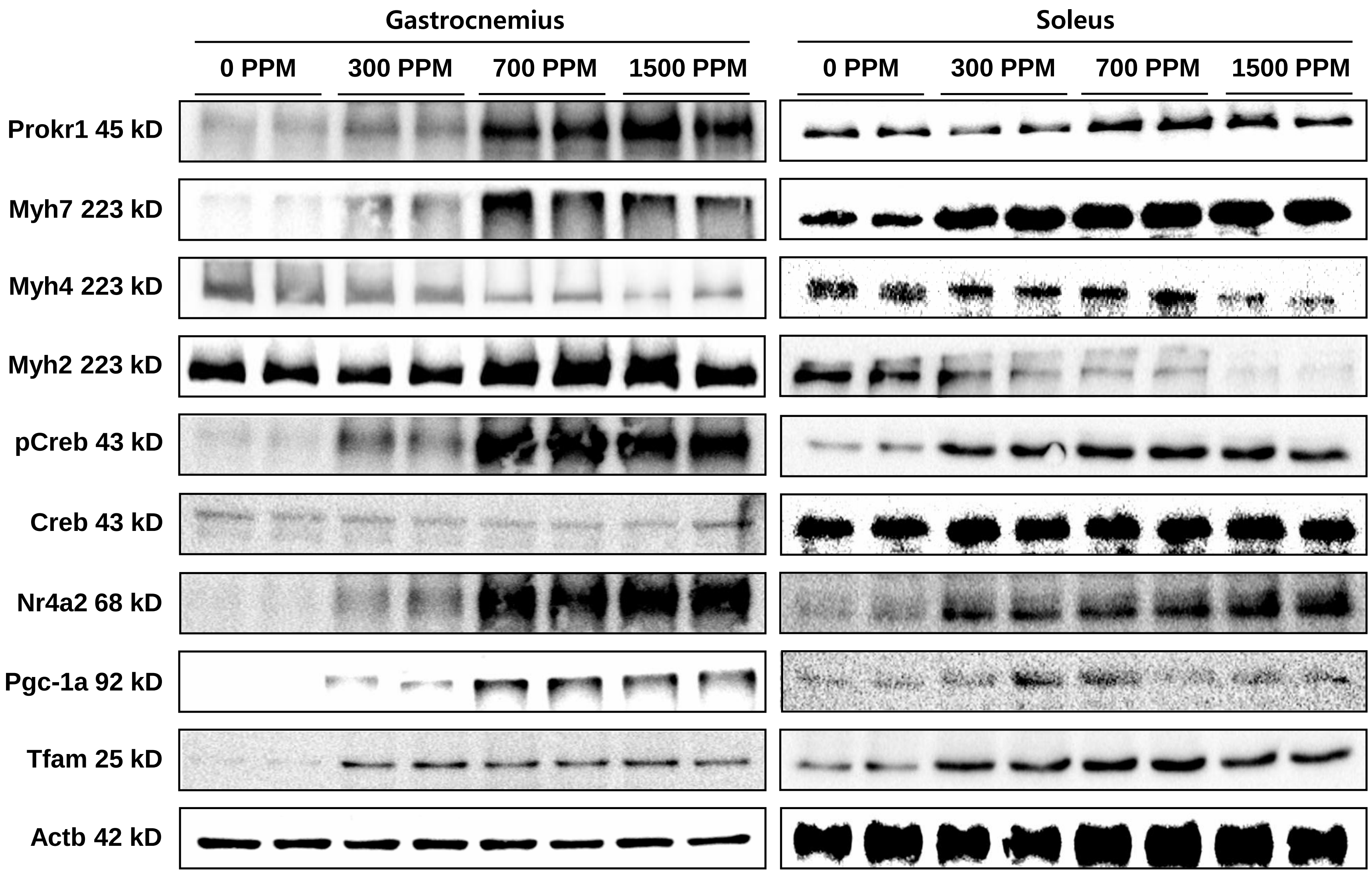

Supplementary Fig. 22

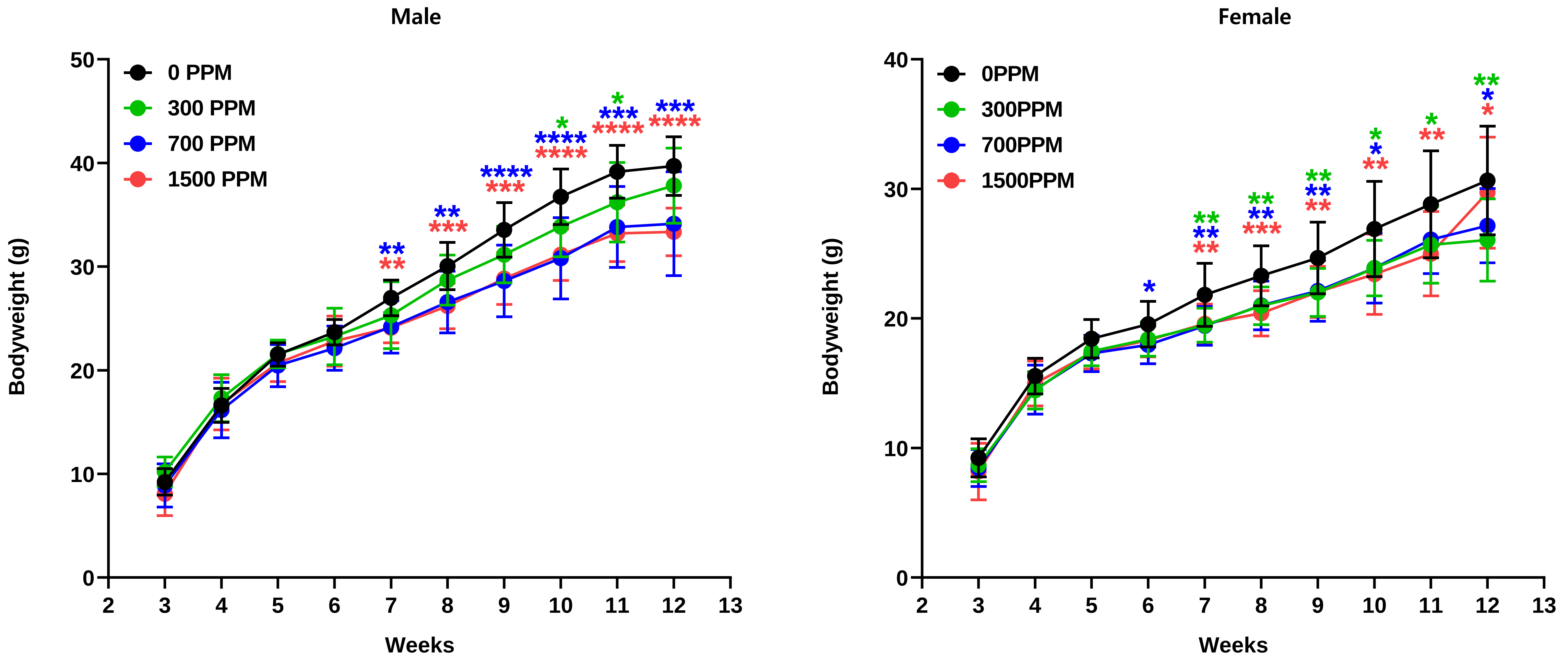

## Supplementary Fig. 23

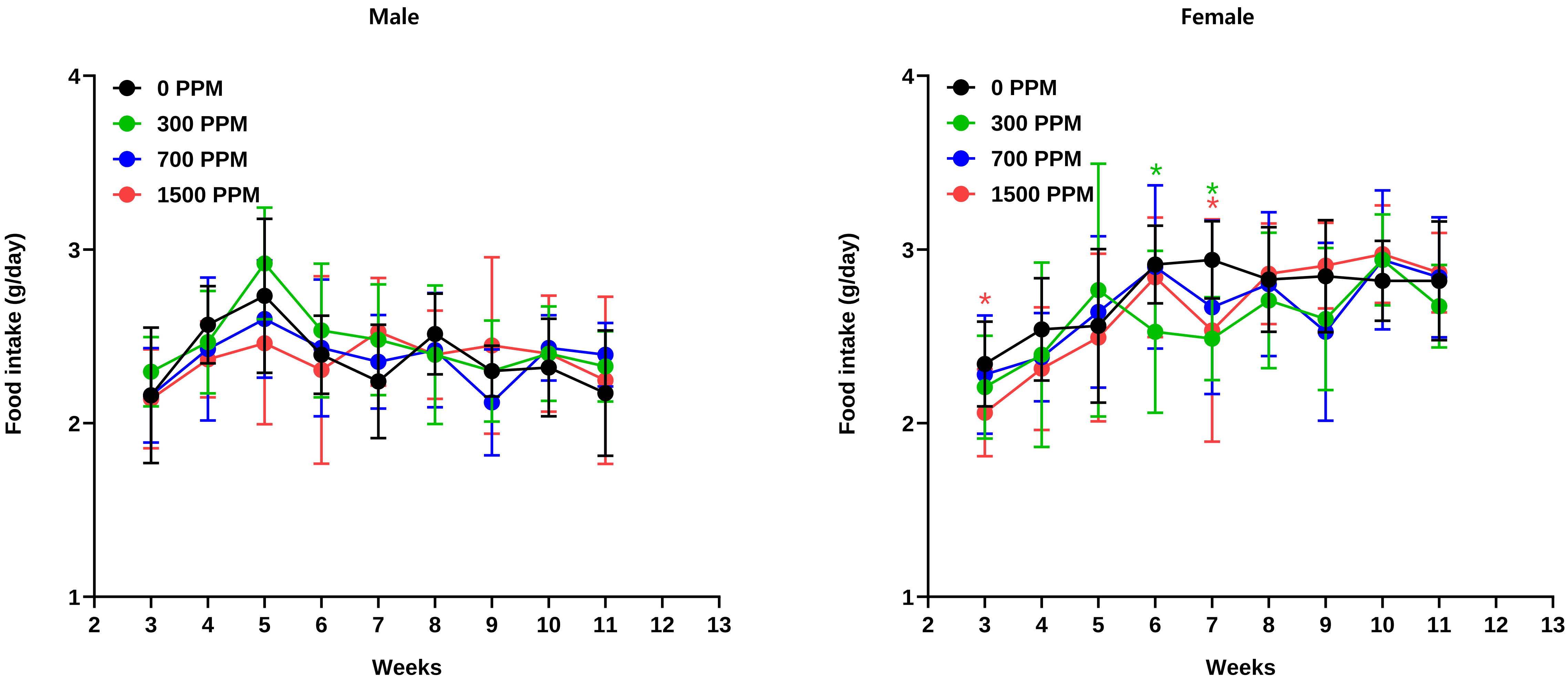

# Supplementary Fig. 24

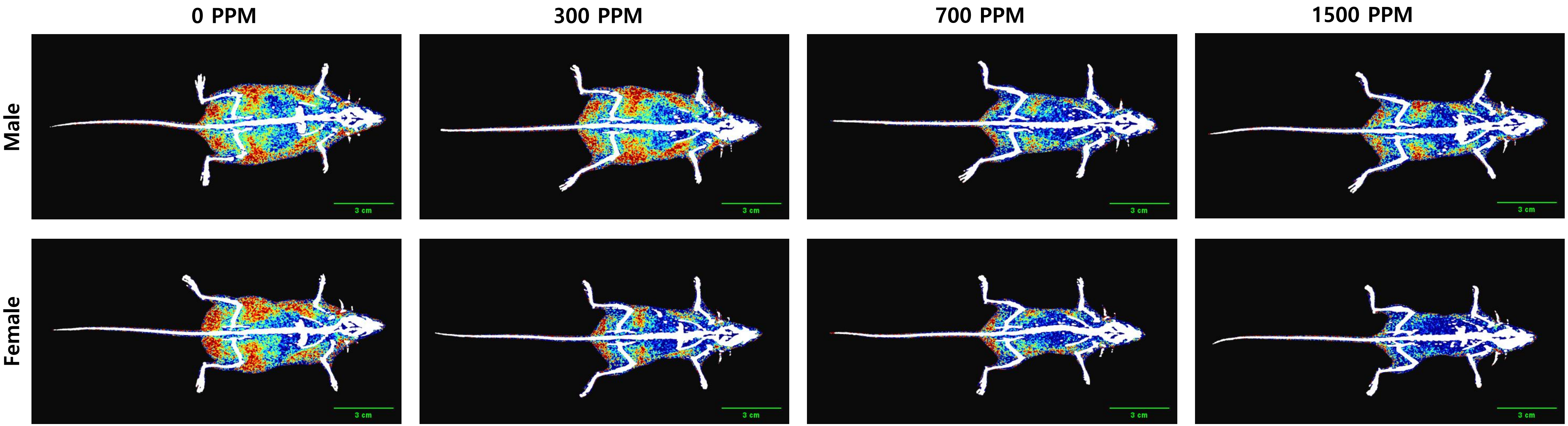

**Supplementary Fig. 25**

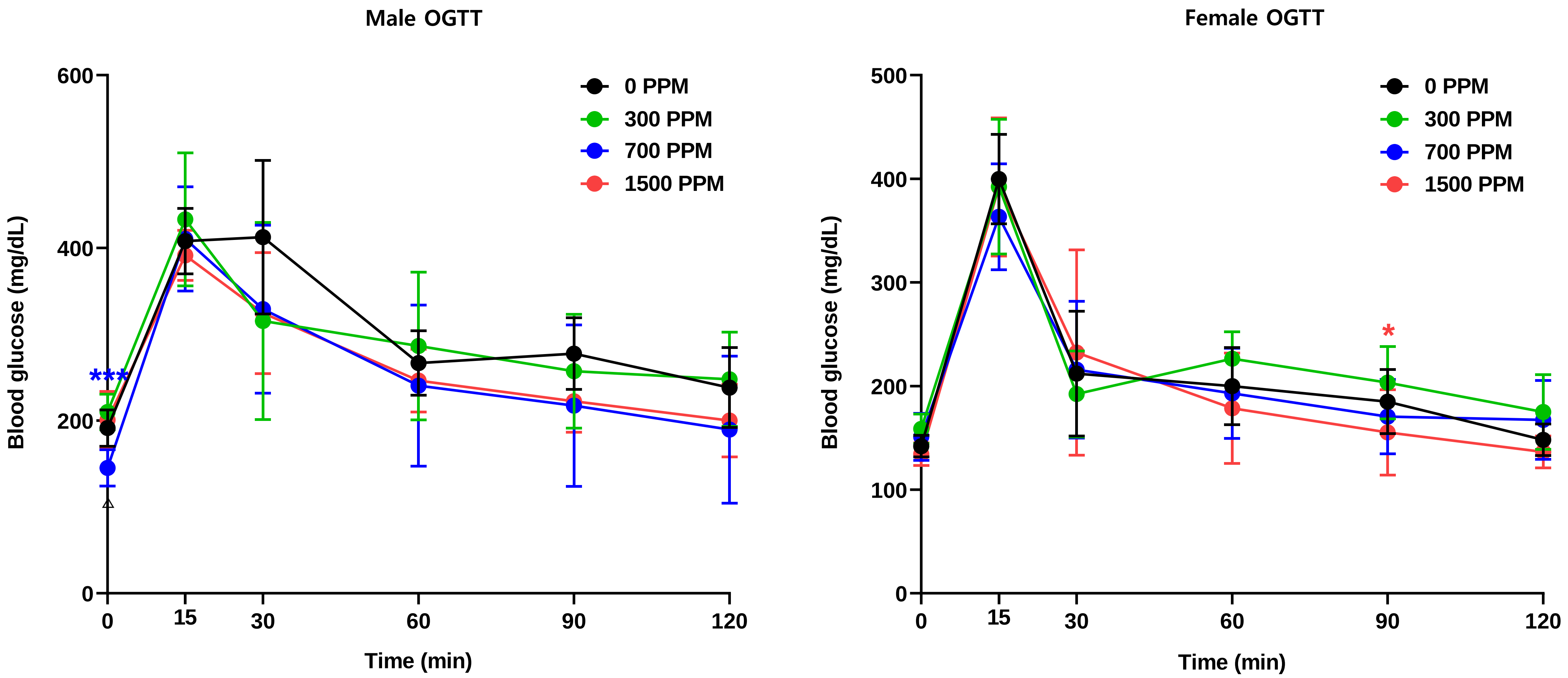

# Supplementary Fig. 26

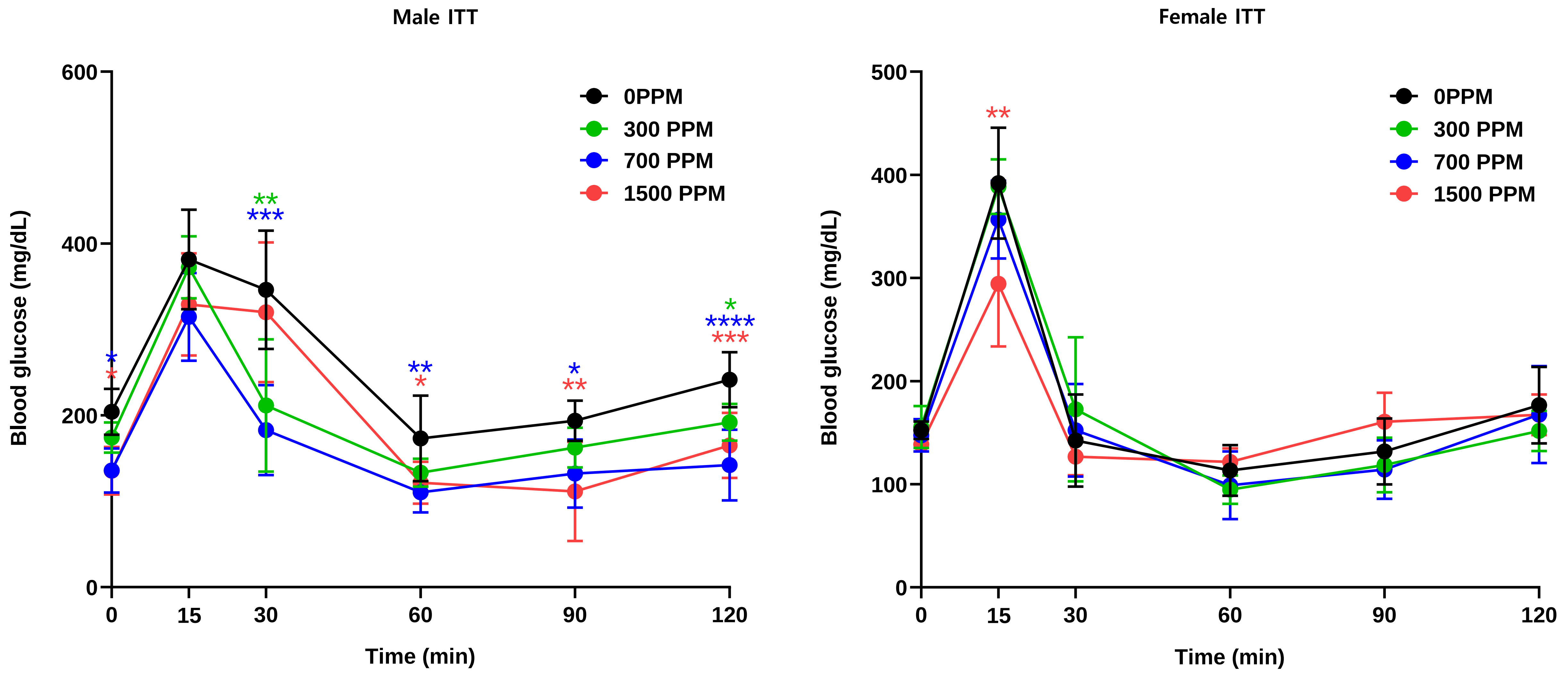

# Supplementary Fig. 27

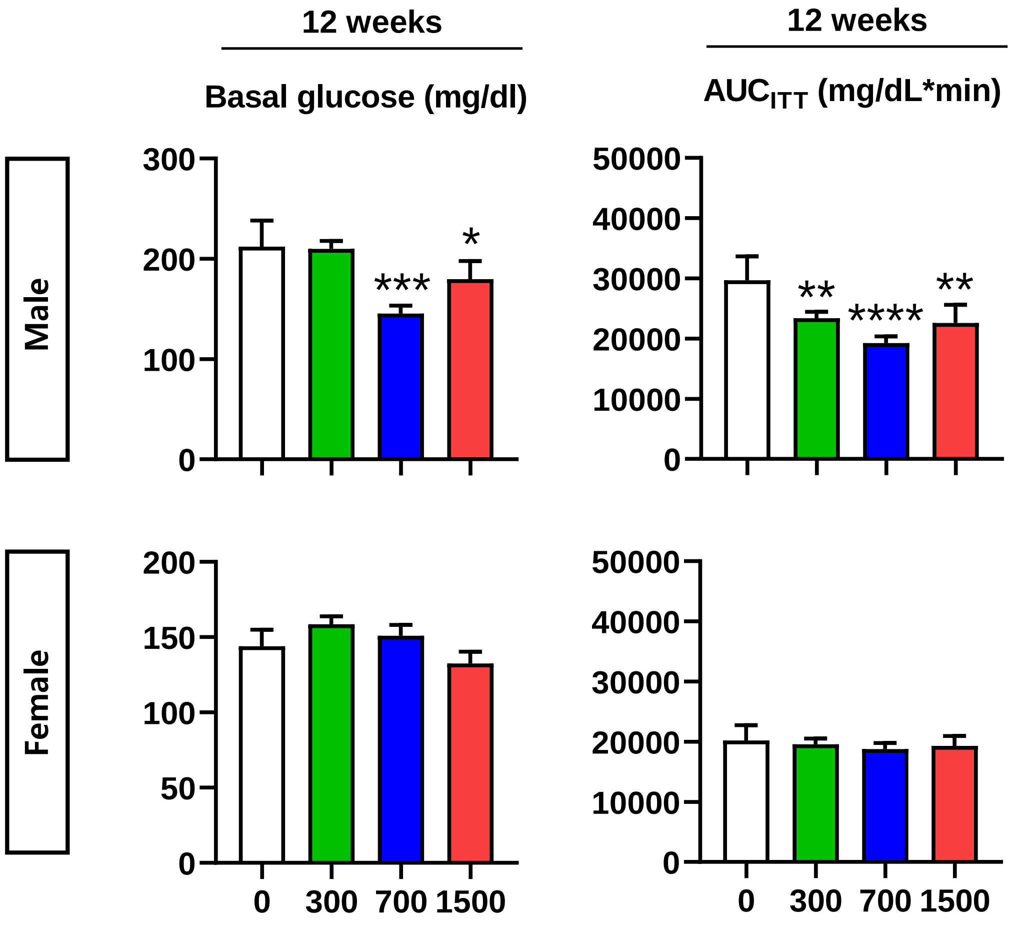

# Supplementary Fig. 28

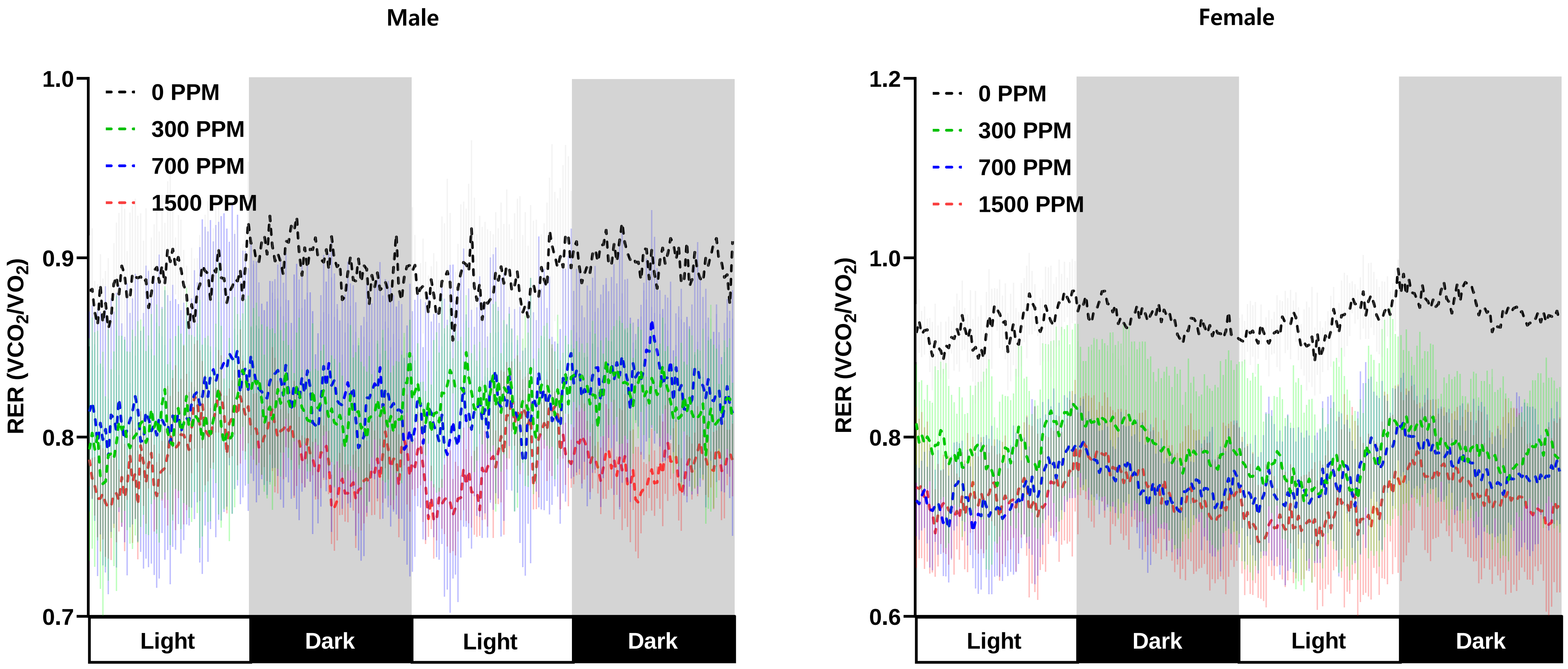

## Supplementary Fig. 29

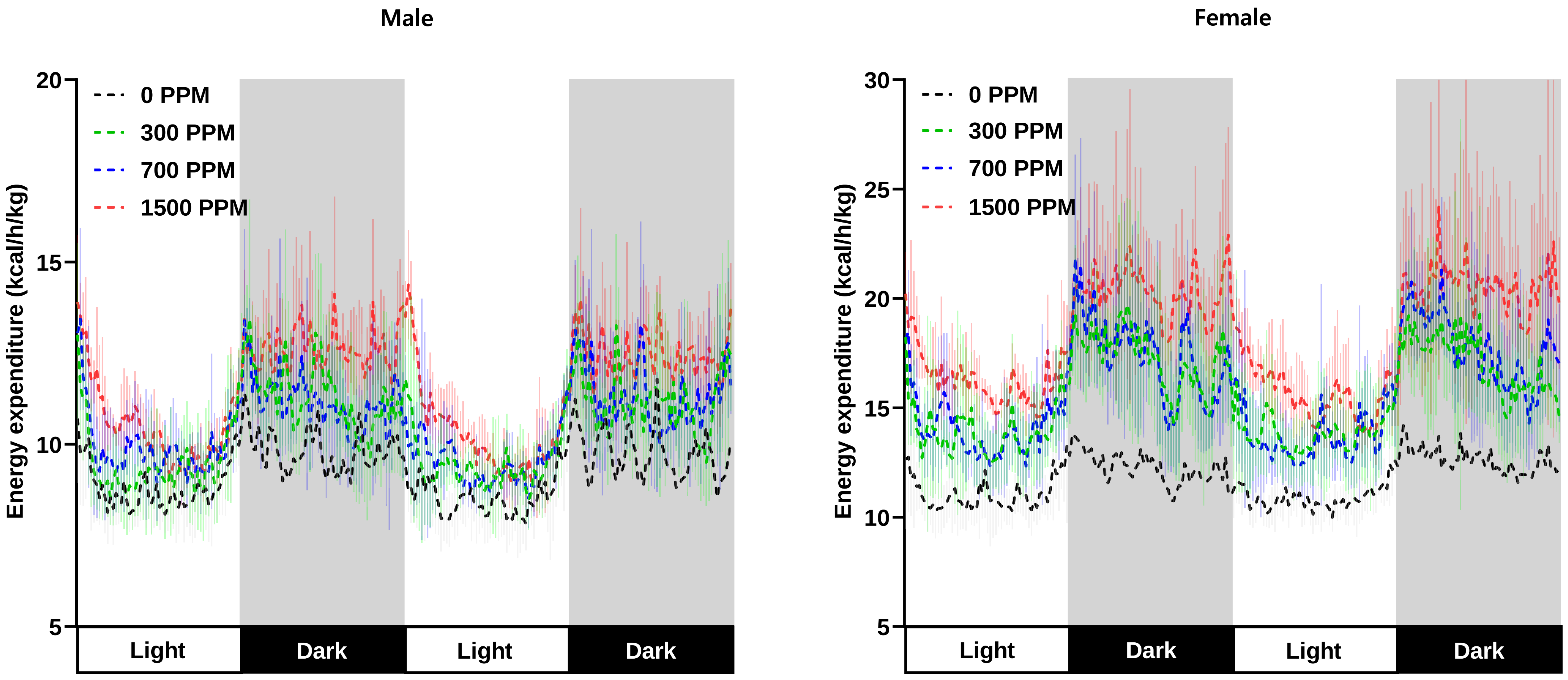

# Supplementary Fig. 30

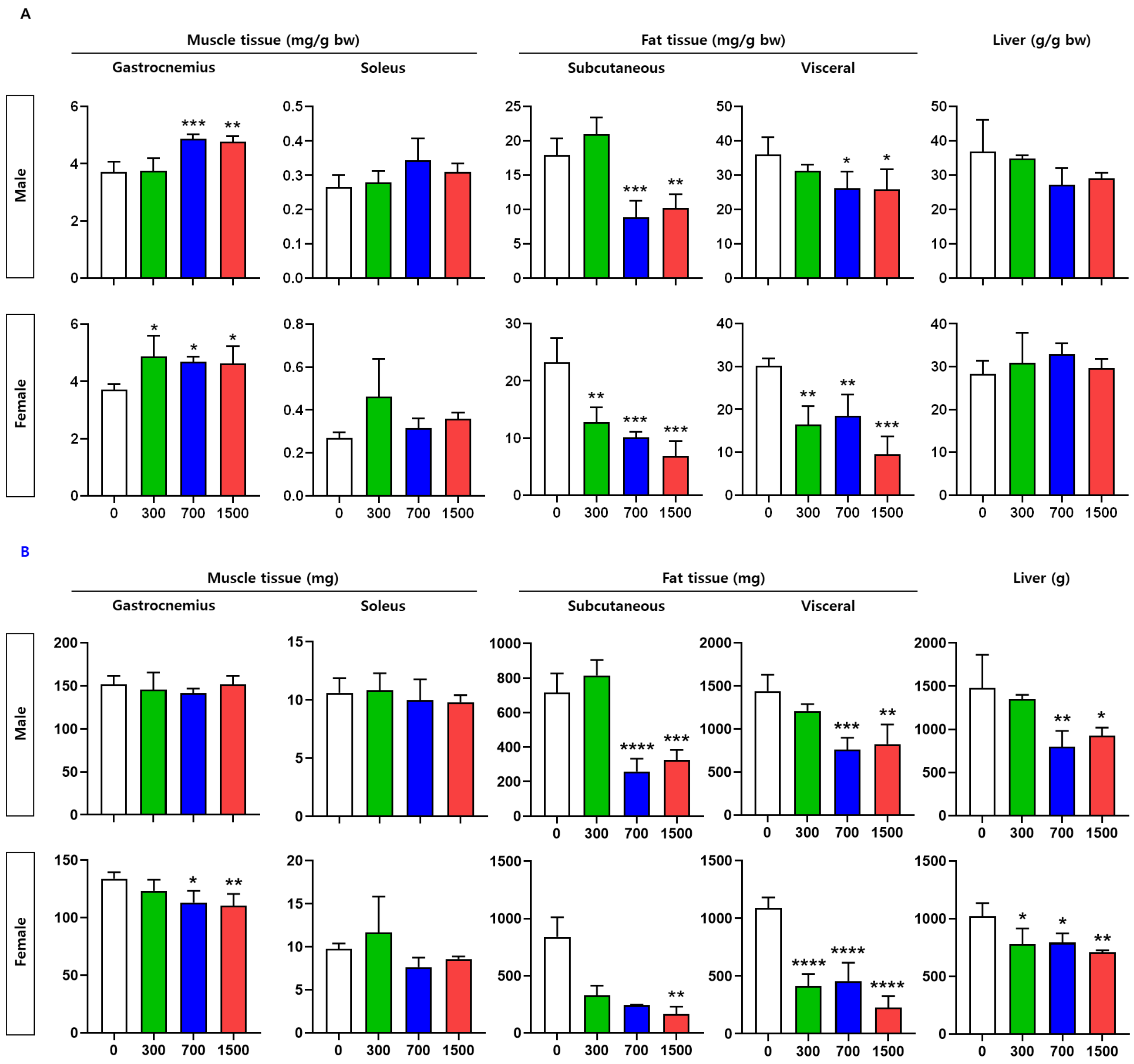

# Supplementary Fig. 31

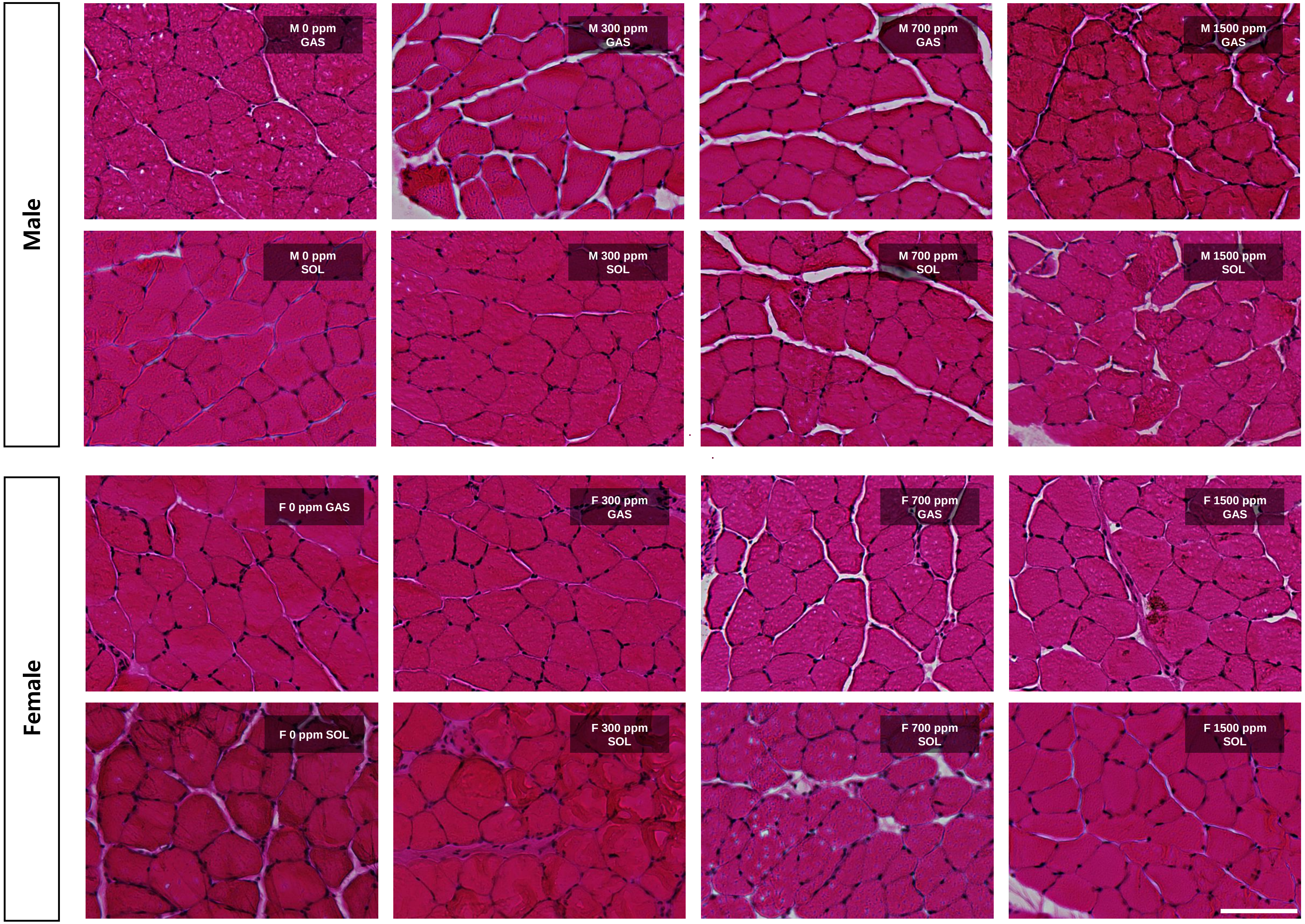

# Supplementary Fig. 32

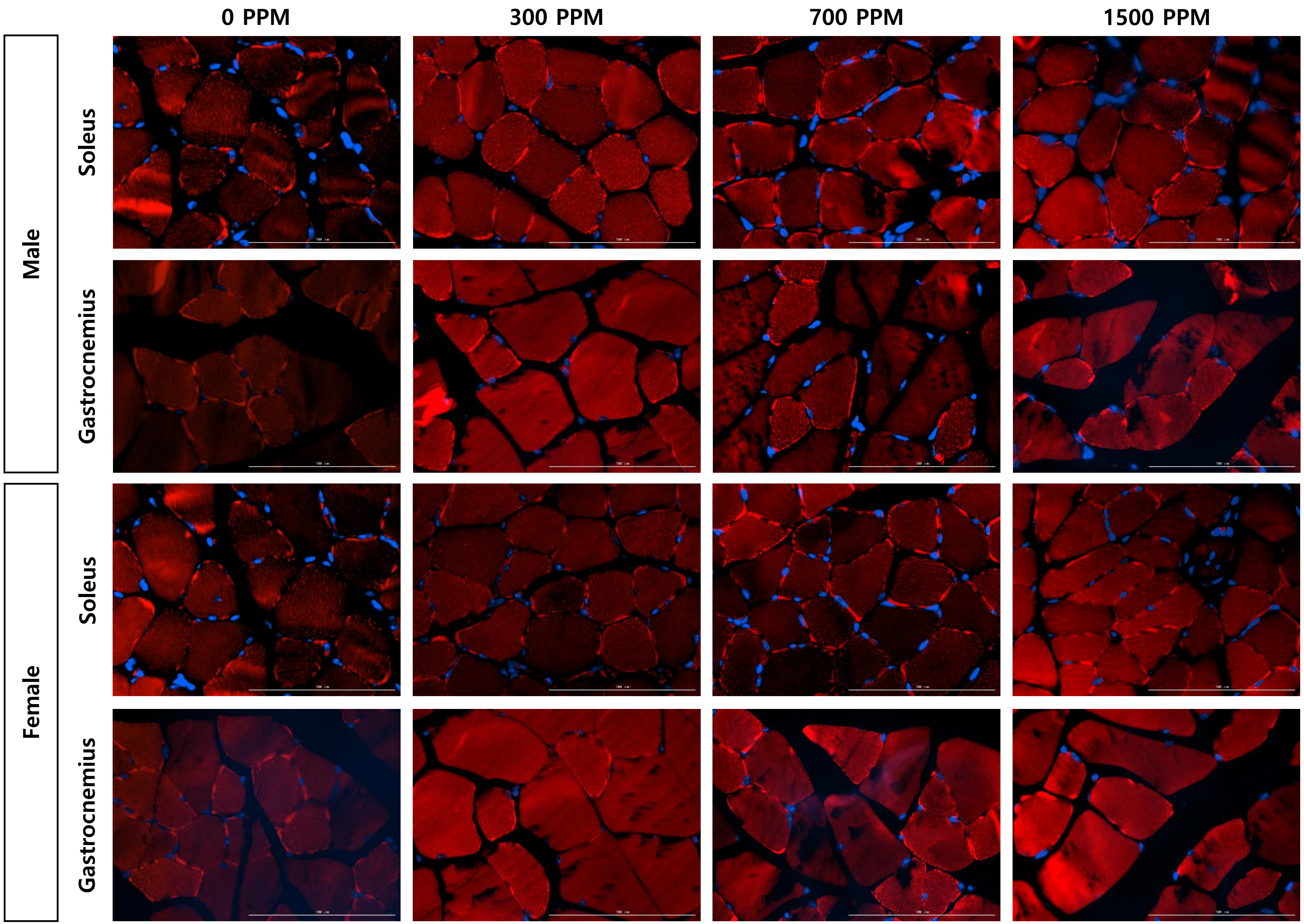

Supplementary Fig. 33

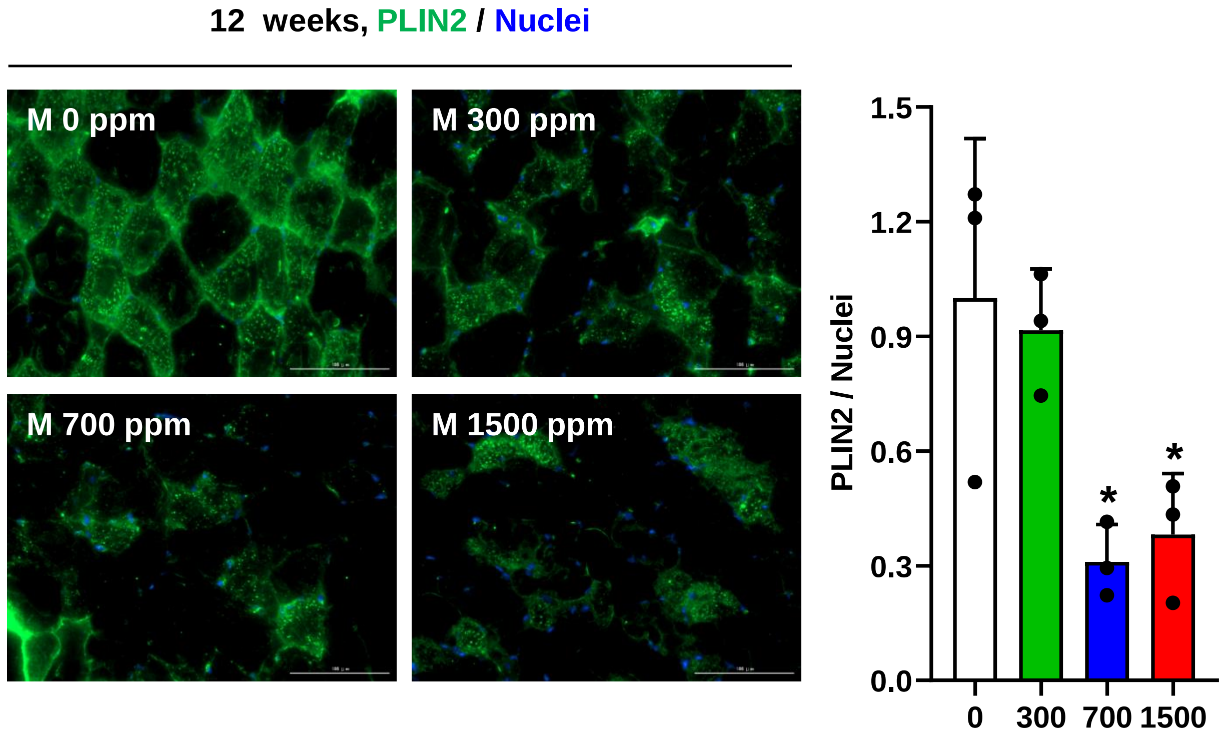


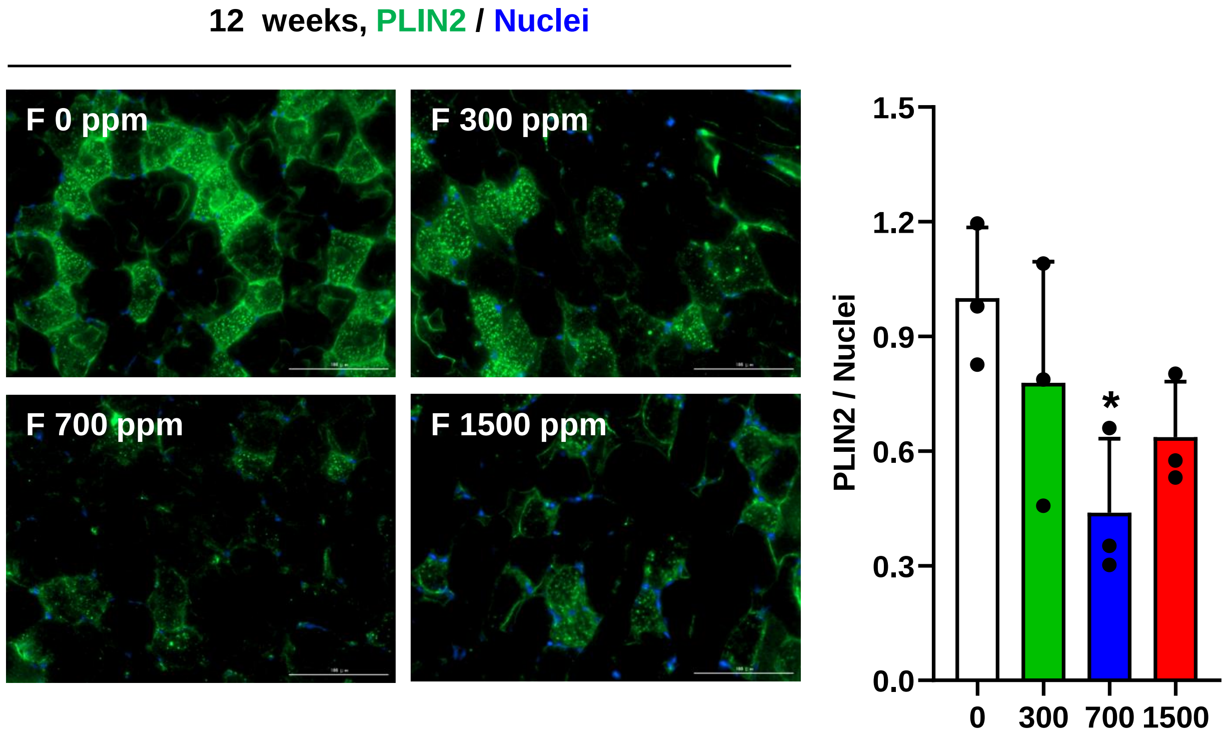

# Supplementary Fig. 34

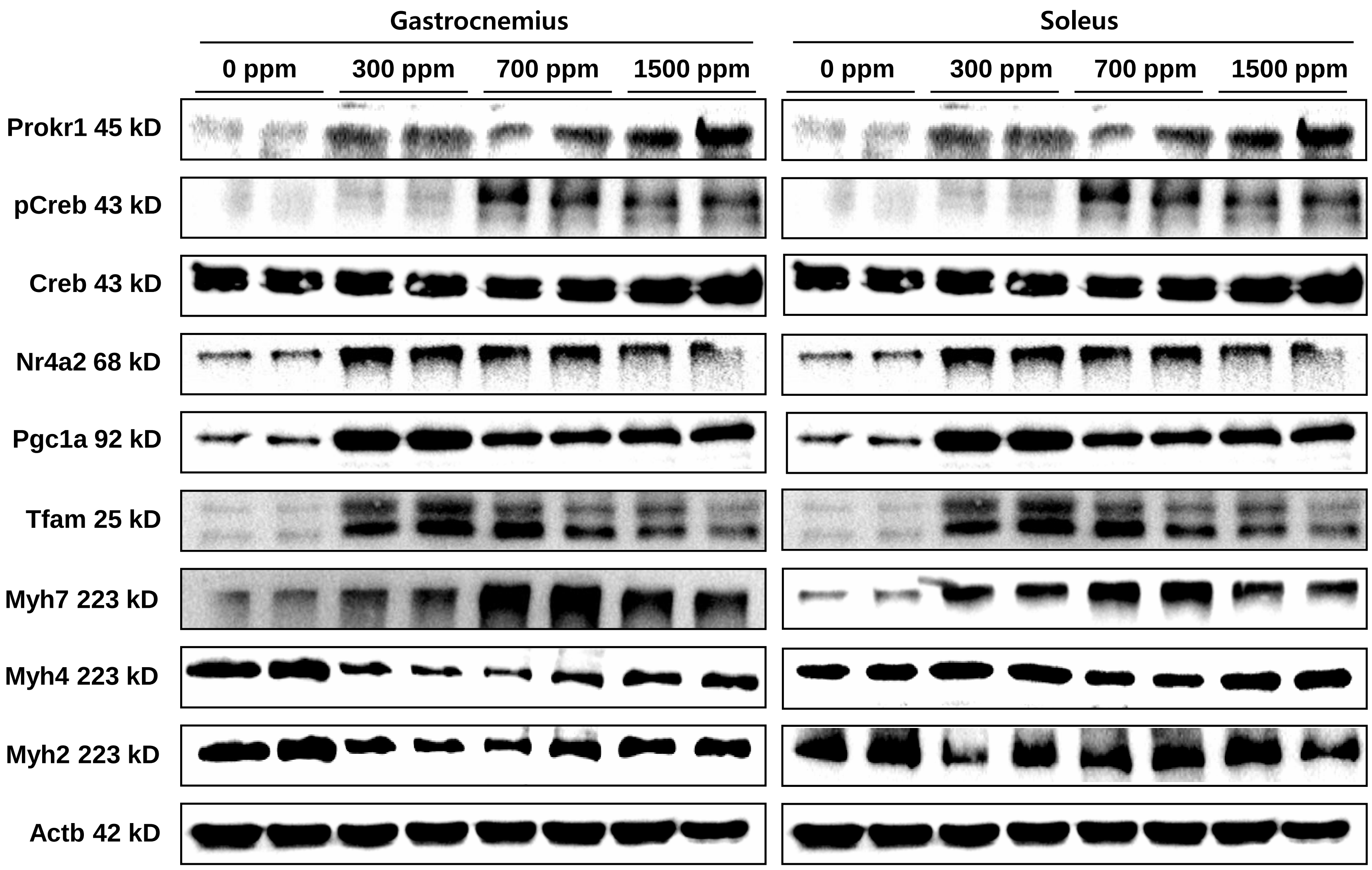

## Supplementary Fig. 35

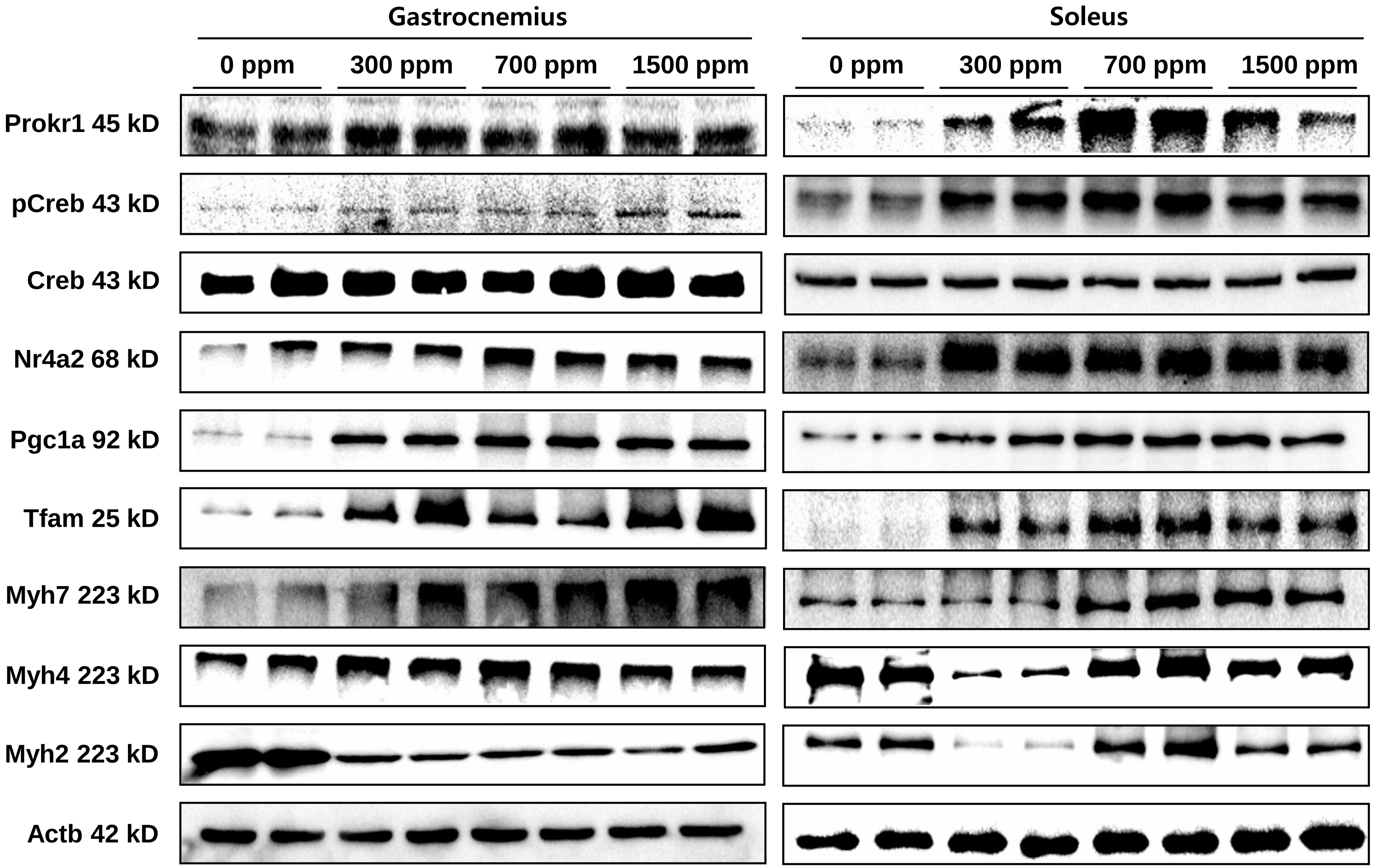

Supplementary Fig. 36

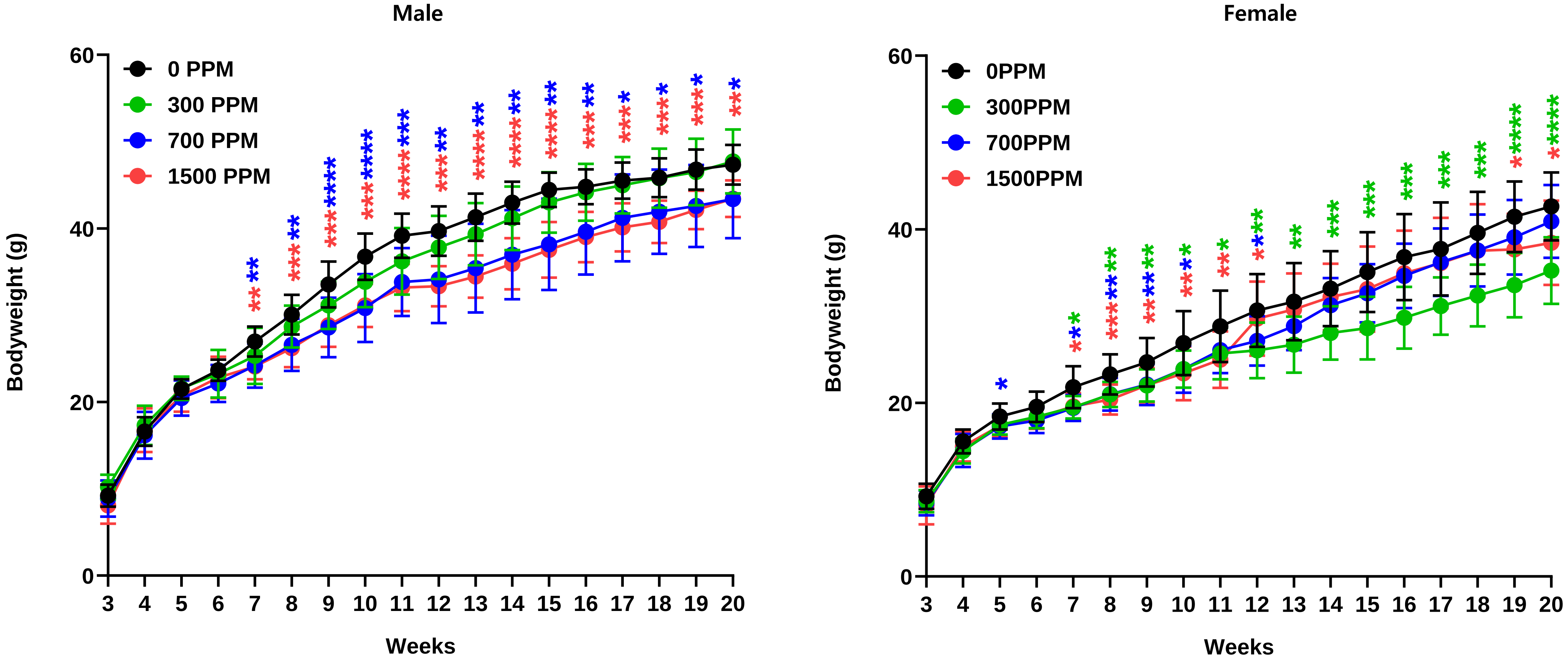

Supplementary Fig. 37

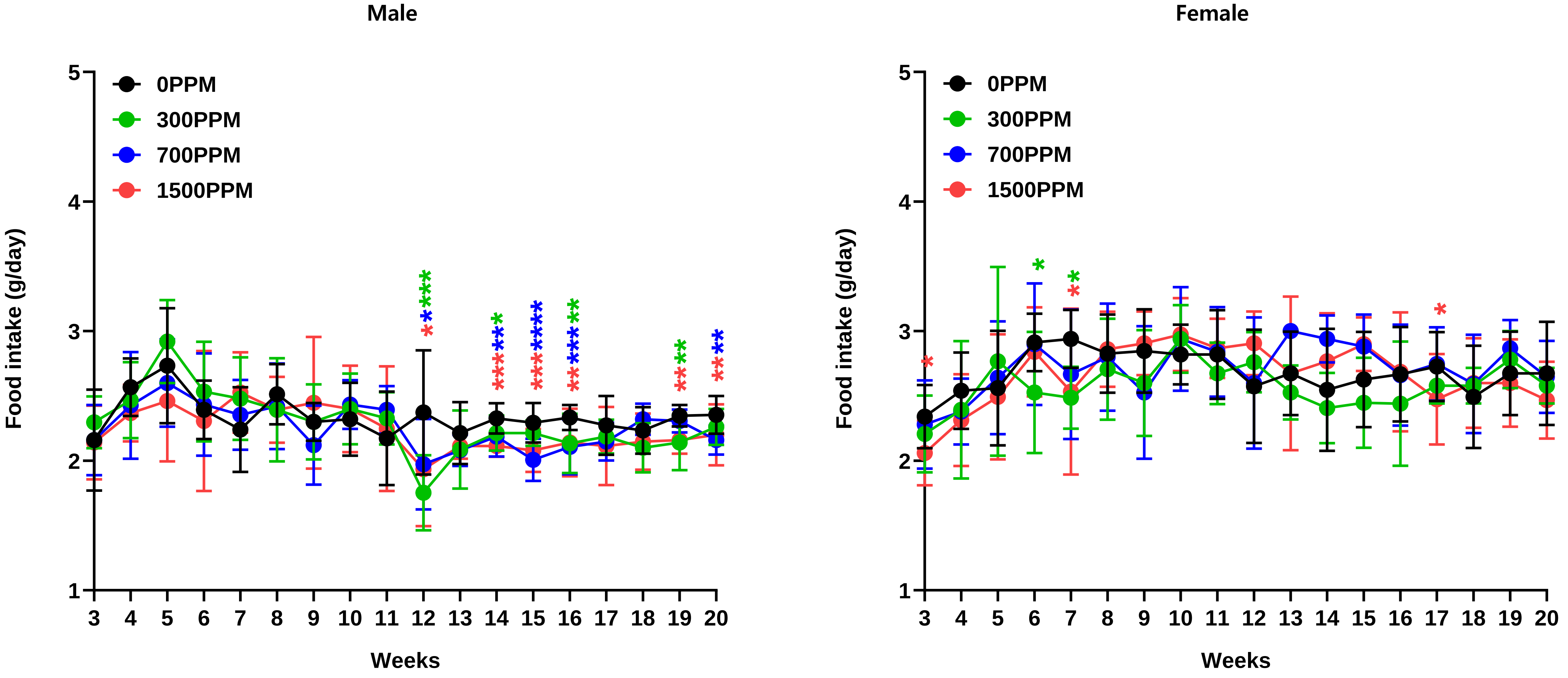

# Supplementary Fig. 38

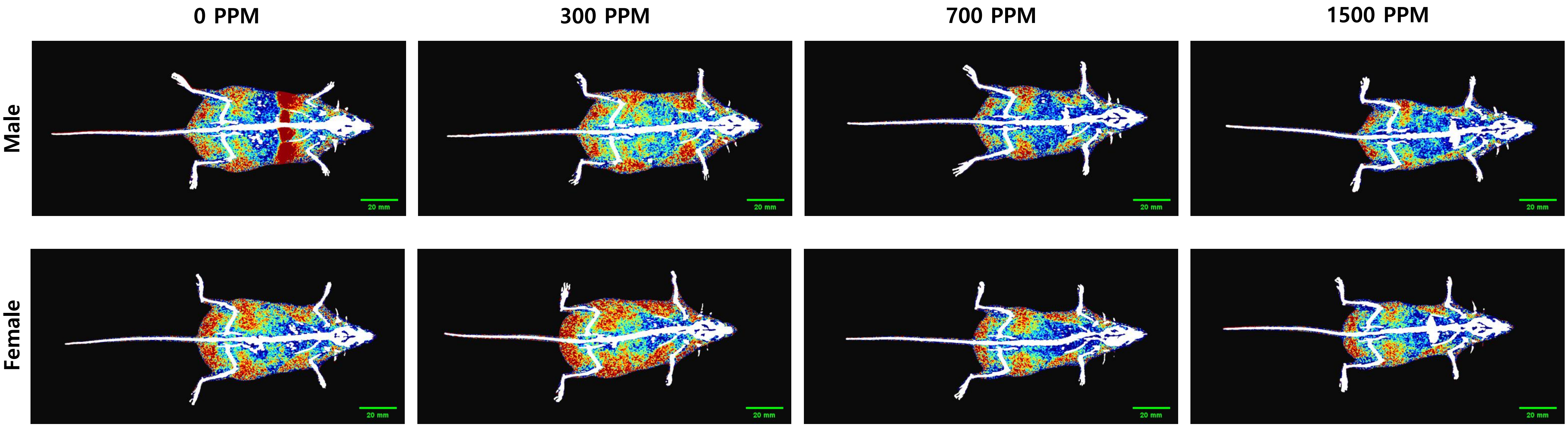

## Supplementary Fig. 39

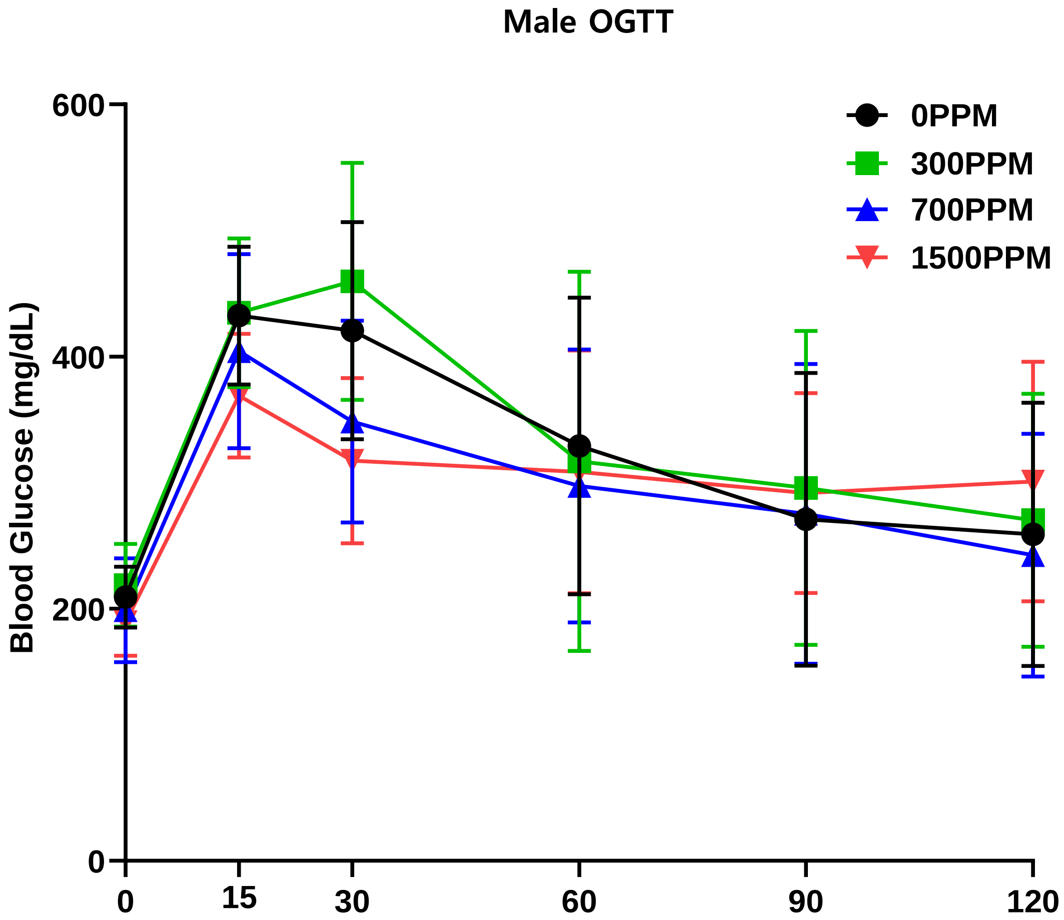


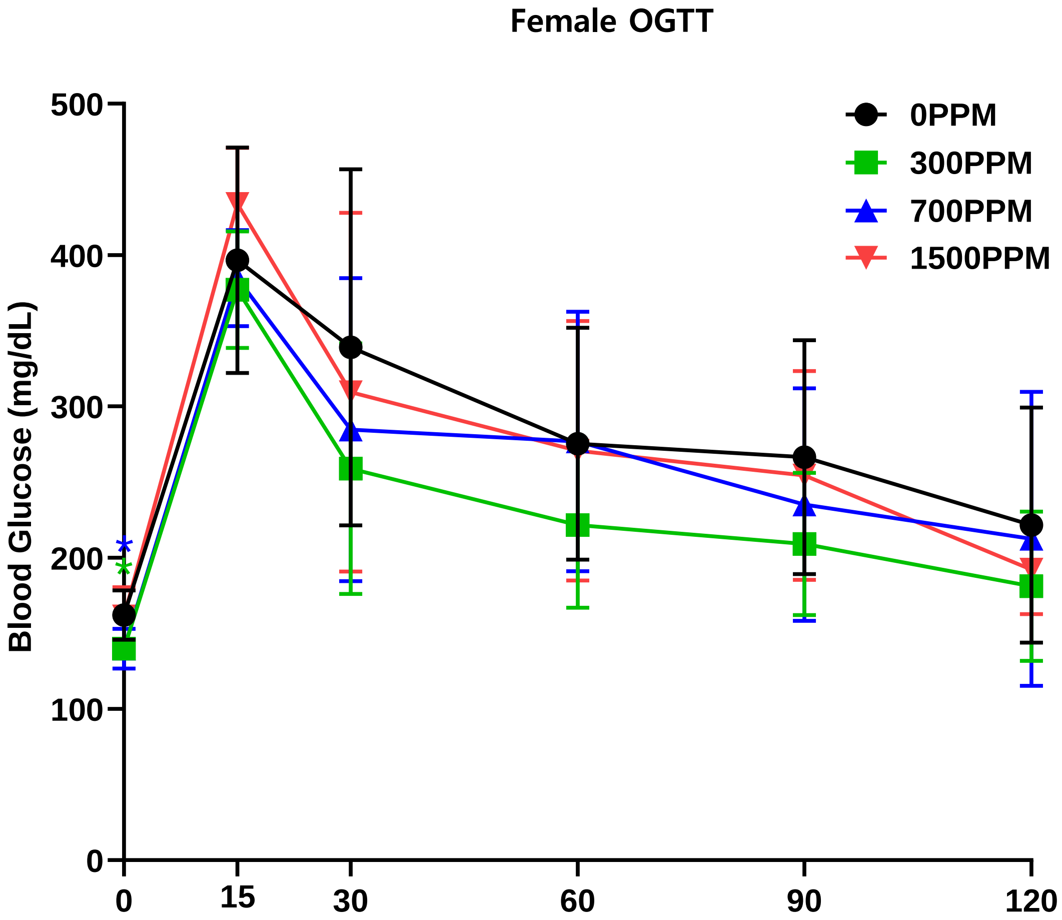

## Supplementary Fig. 40

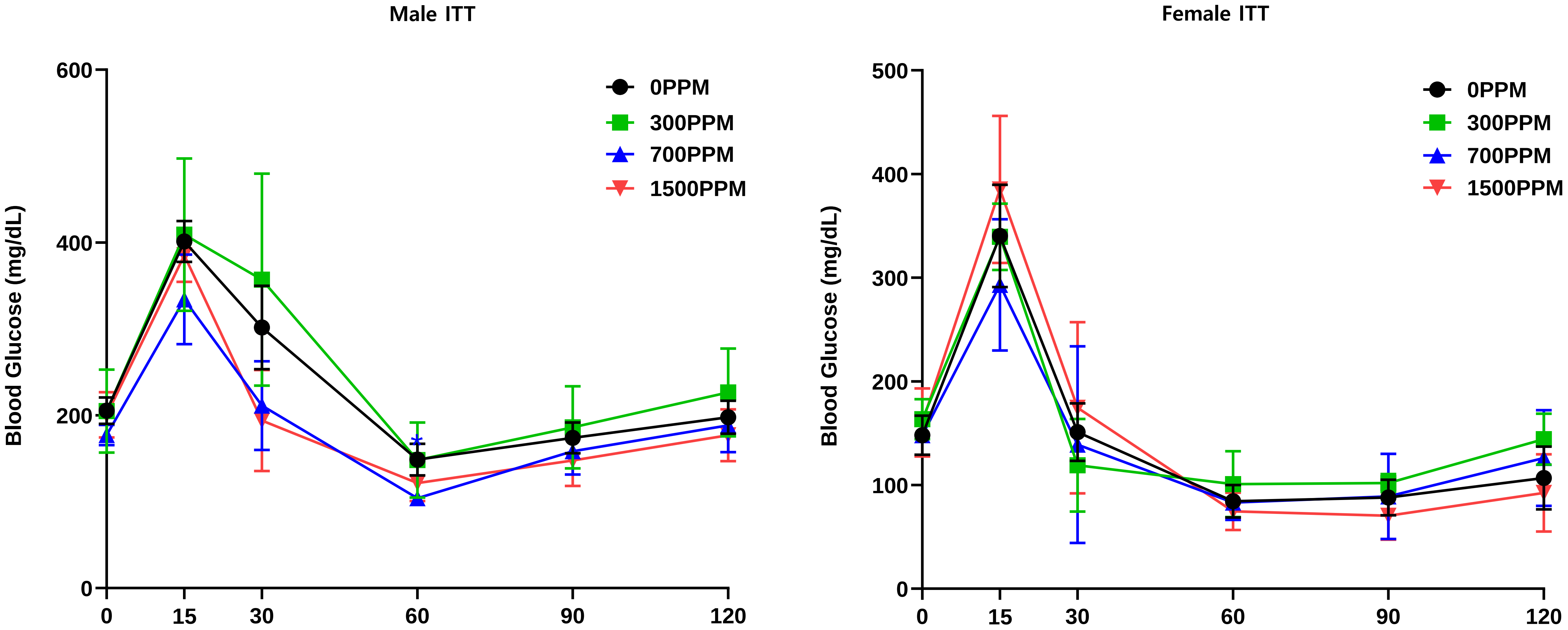

Supplementary Fig. 41

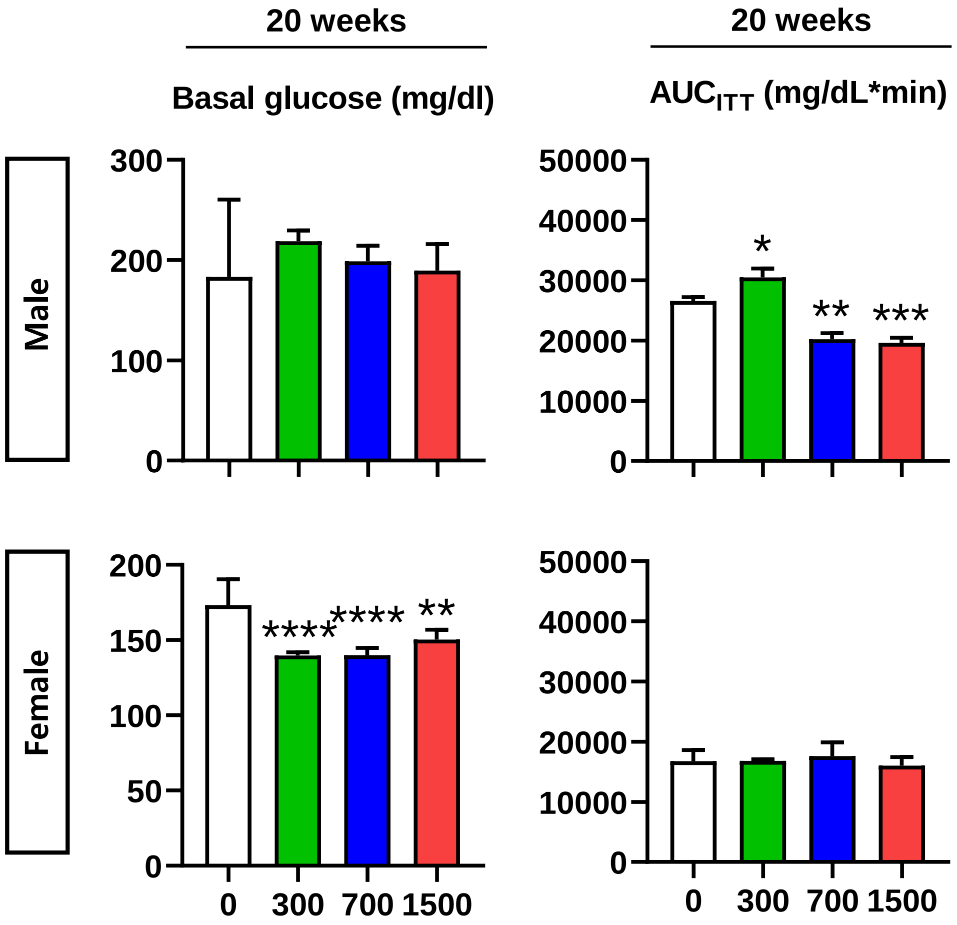

## Supplementary Fig. 42

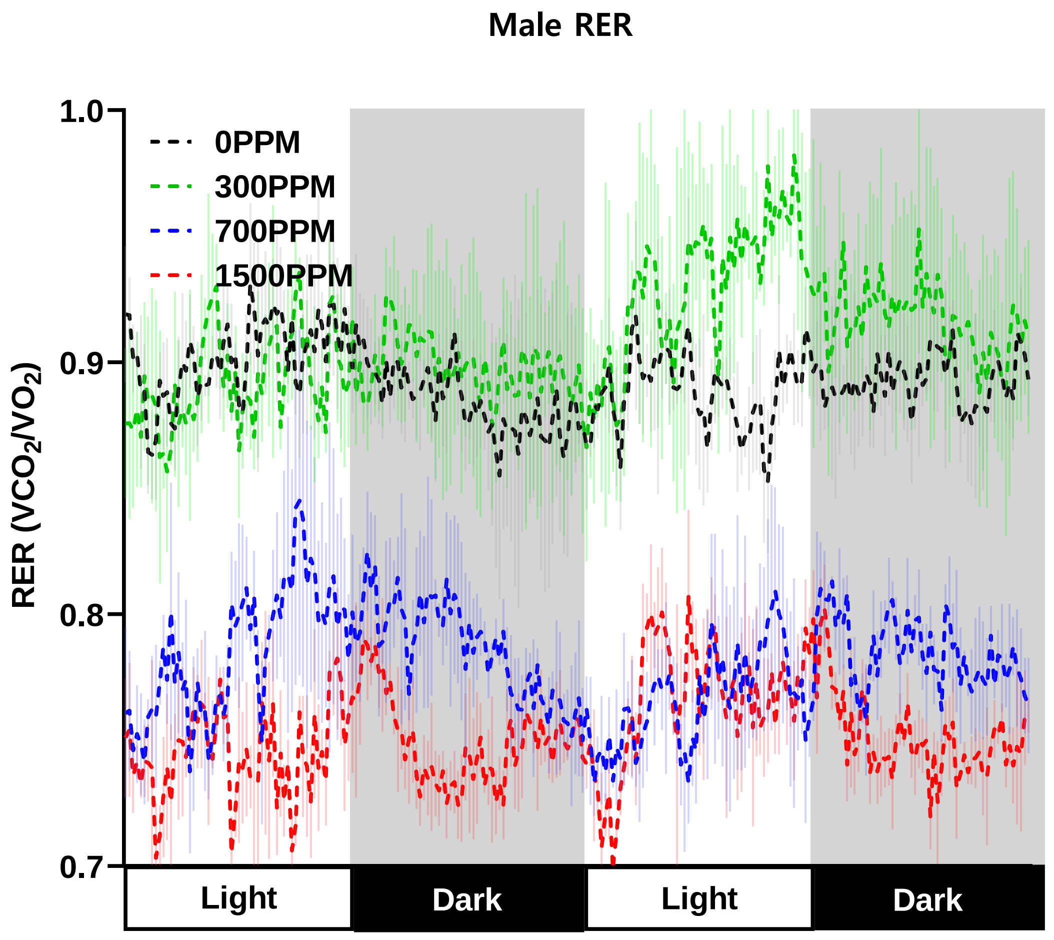


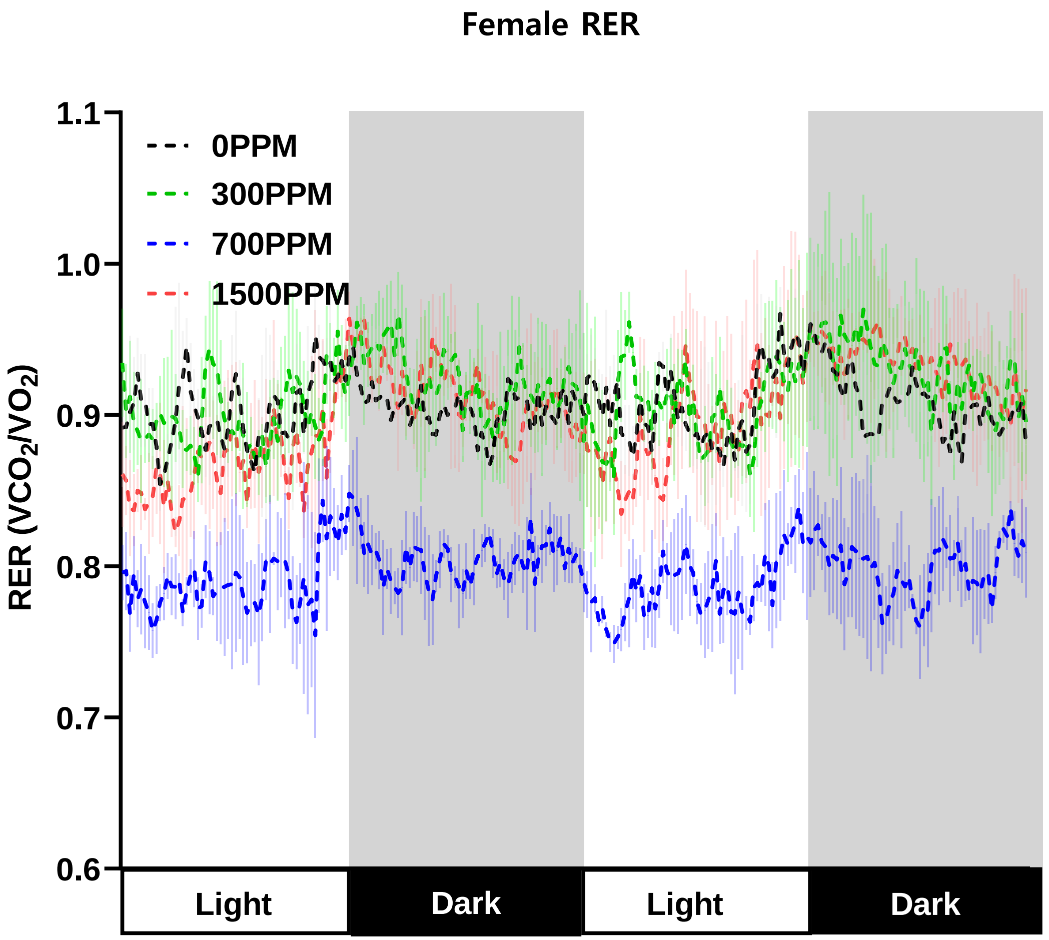

# Supplementary Fig. 43

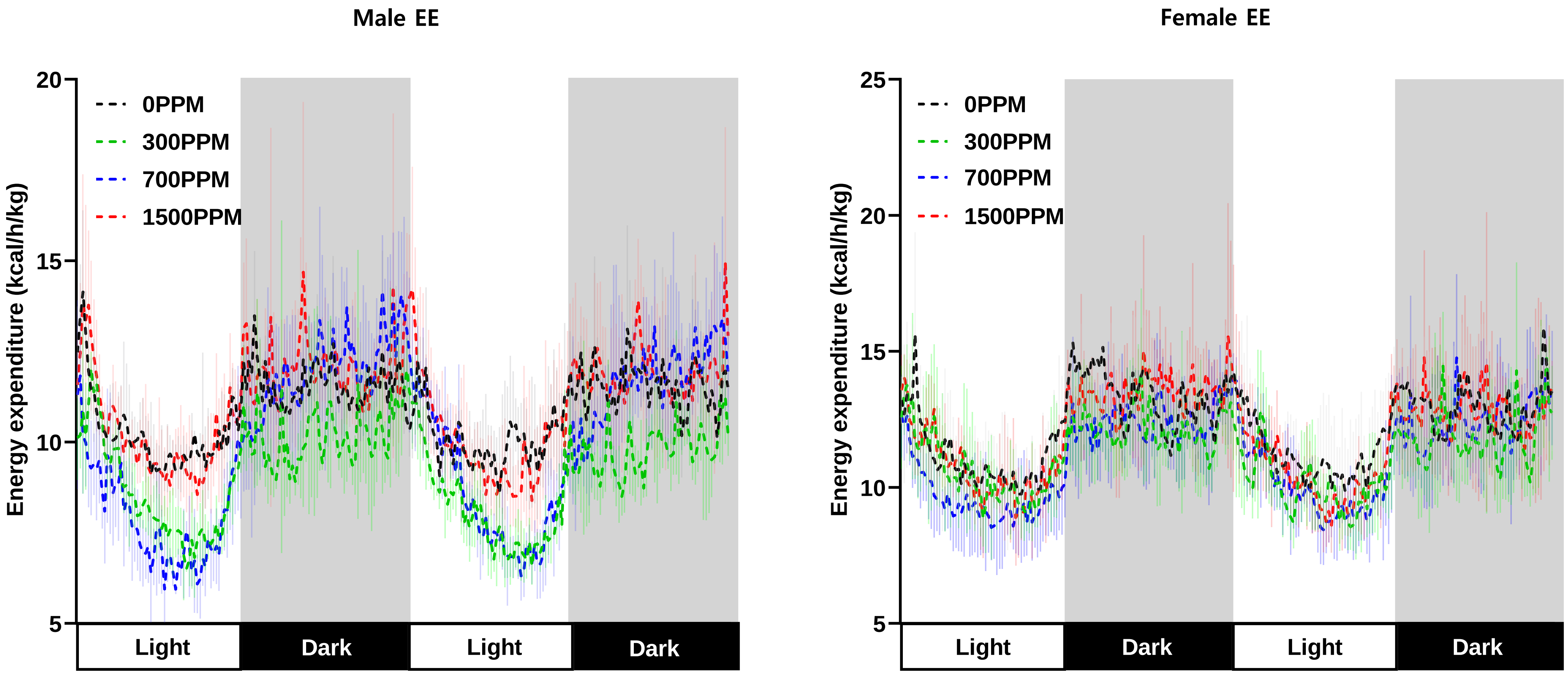

# Supplementary Fig. 44

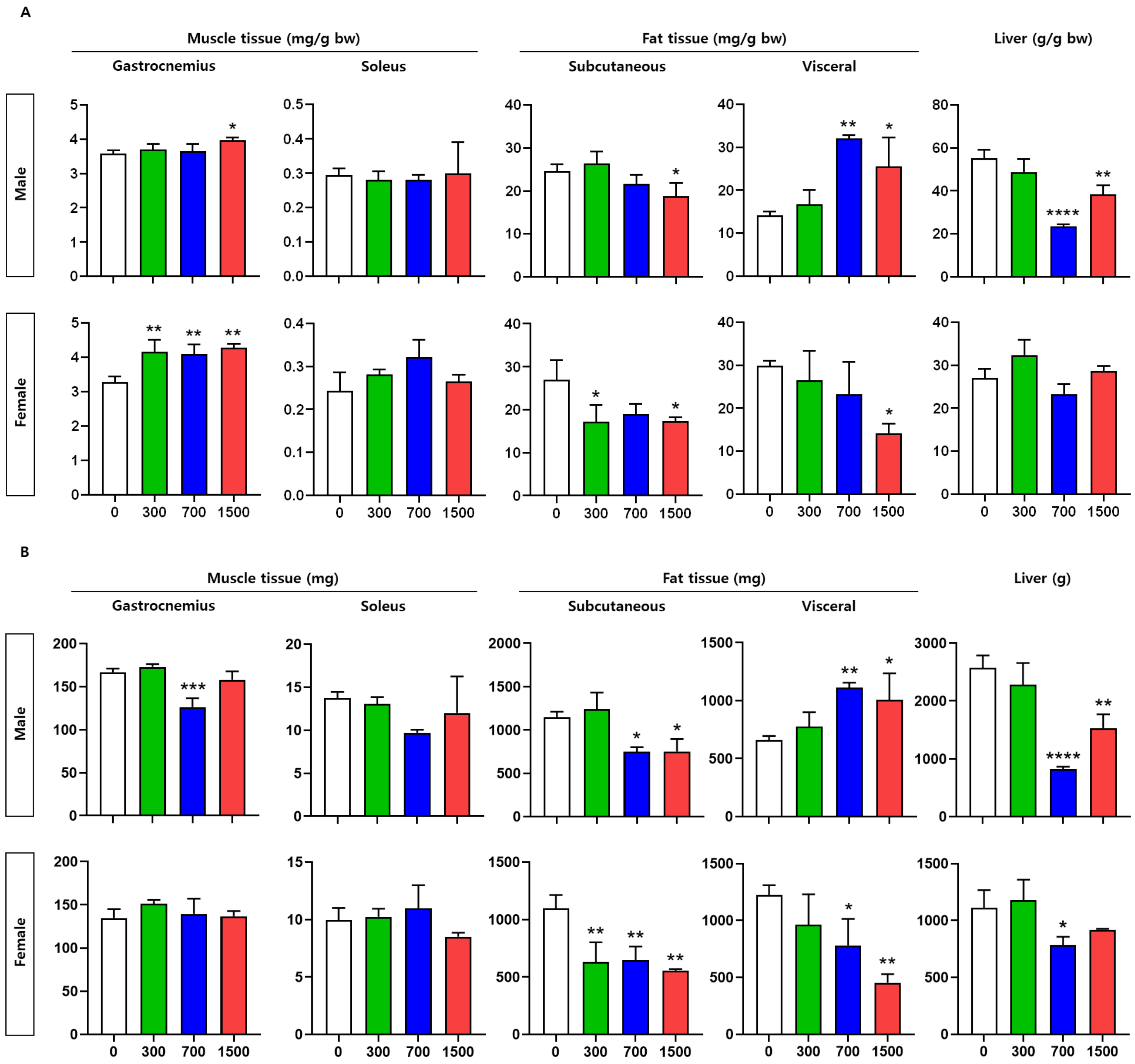

# Supplementary Fig. 45

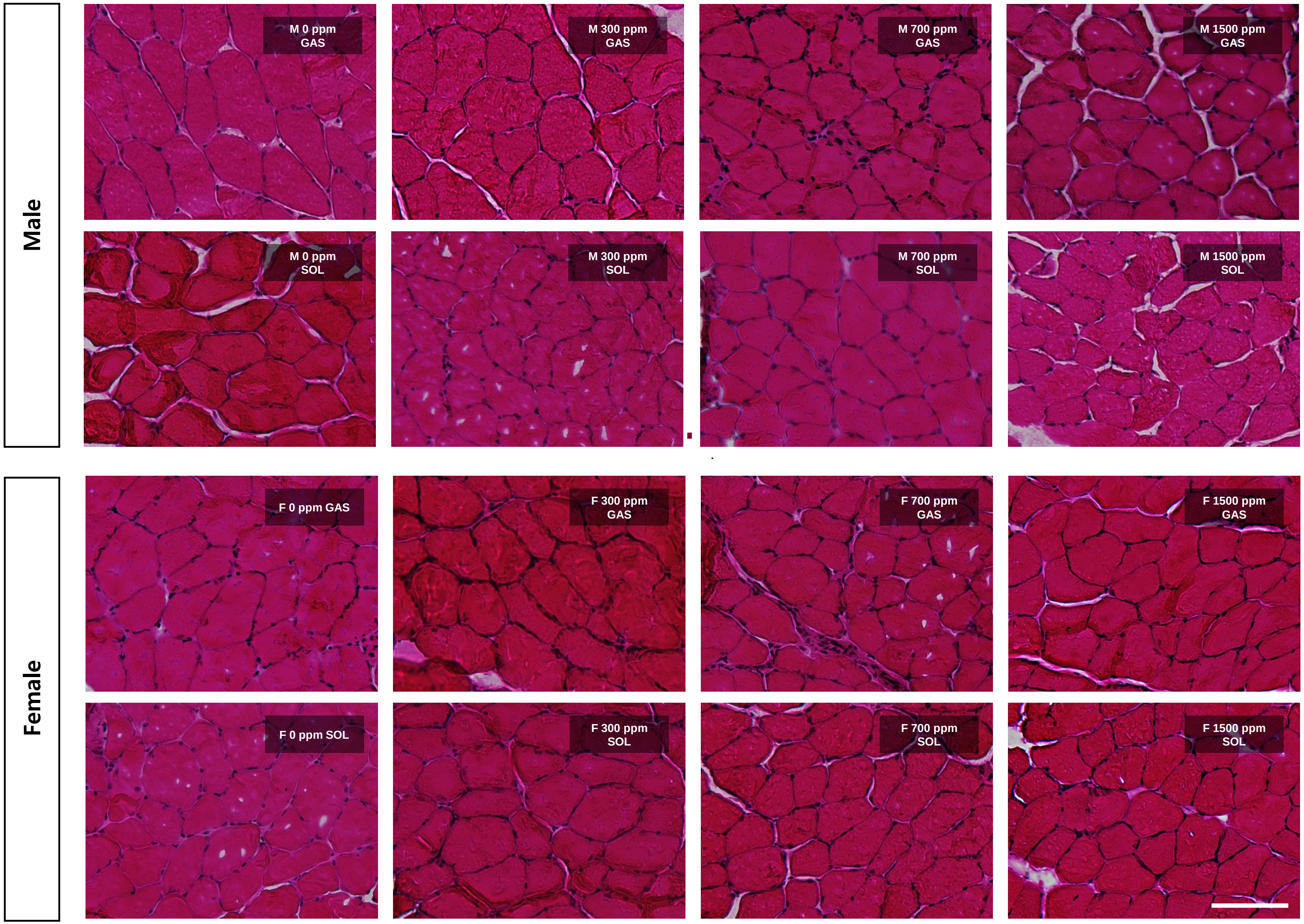

**Supplementary Fig. 46**

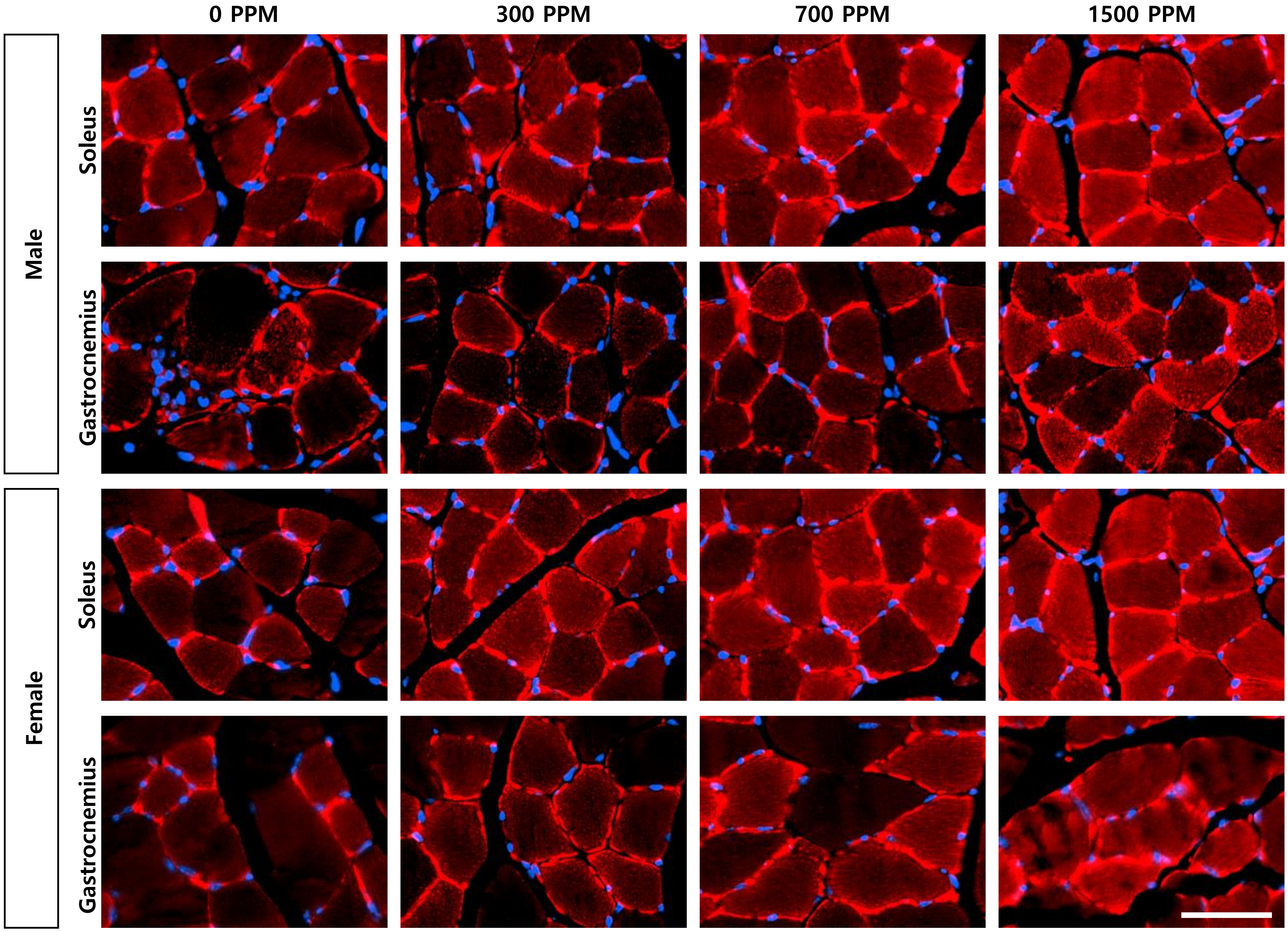

# Supplementary Fig. 47

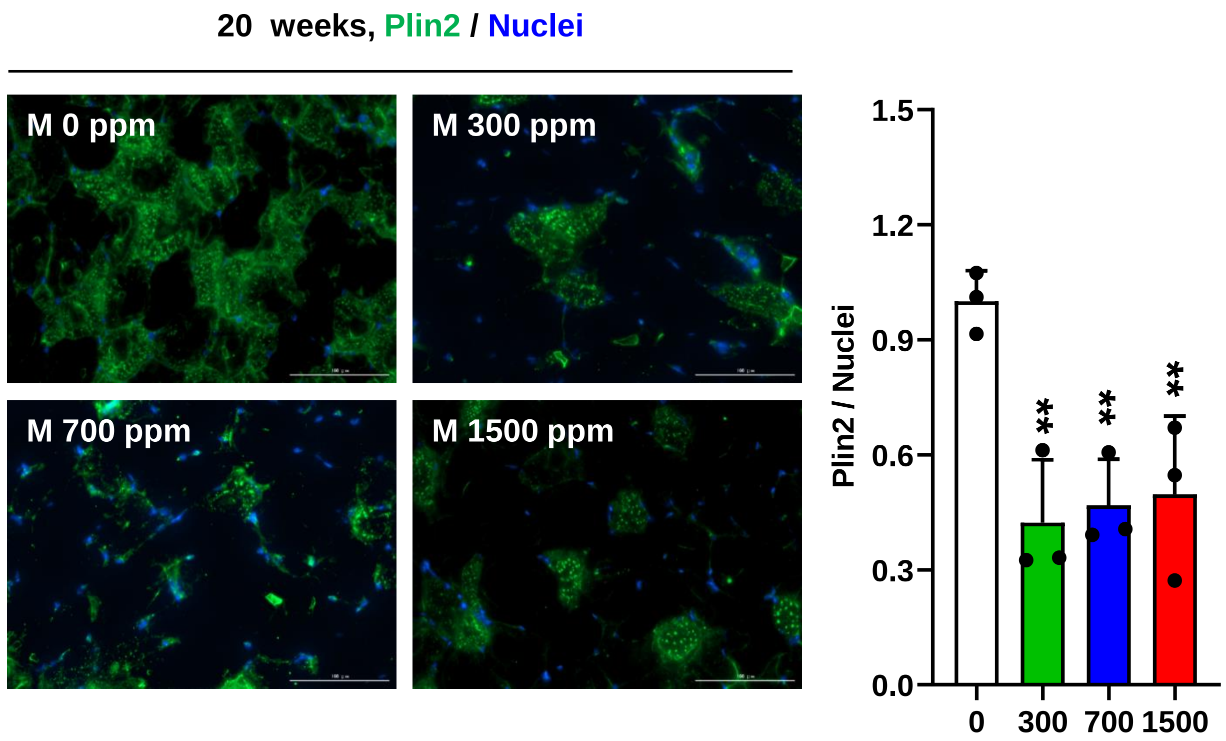


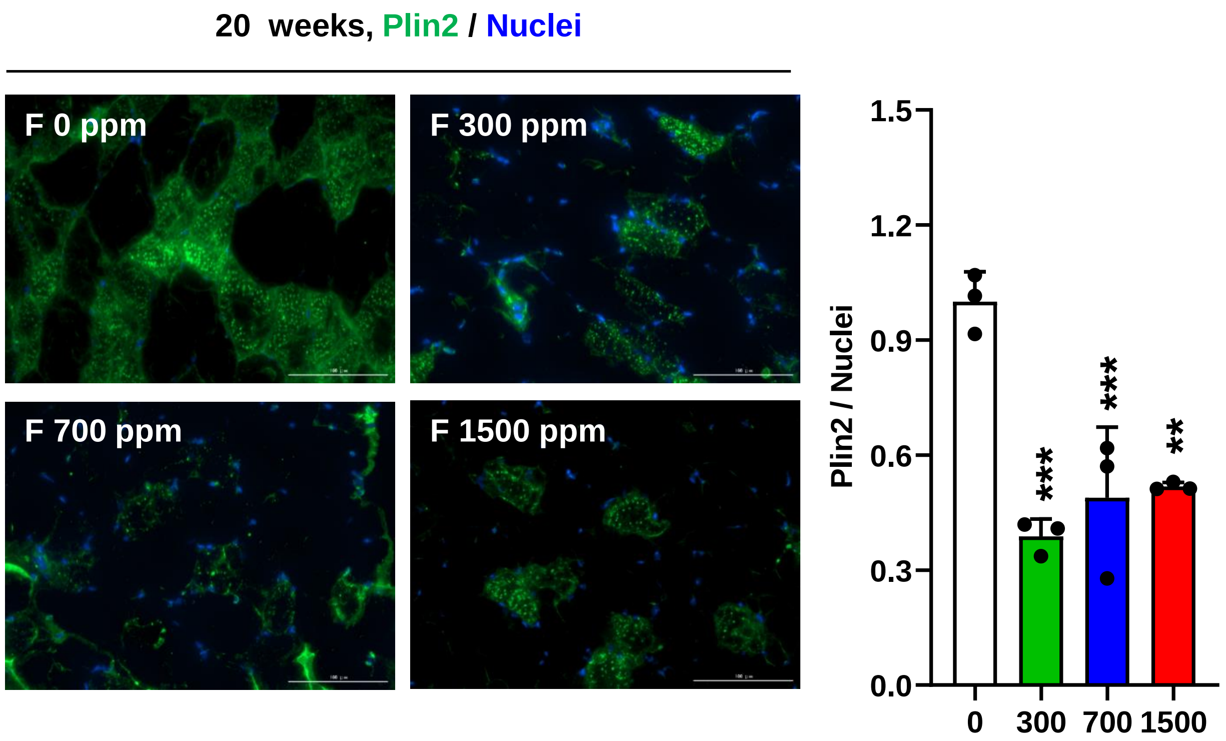

Supplementary Fig. 48

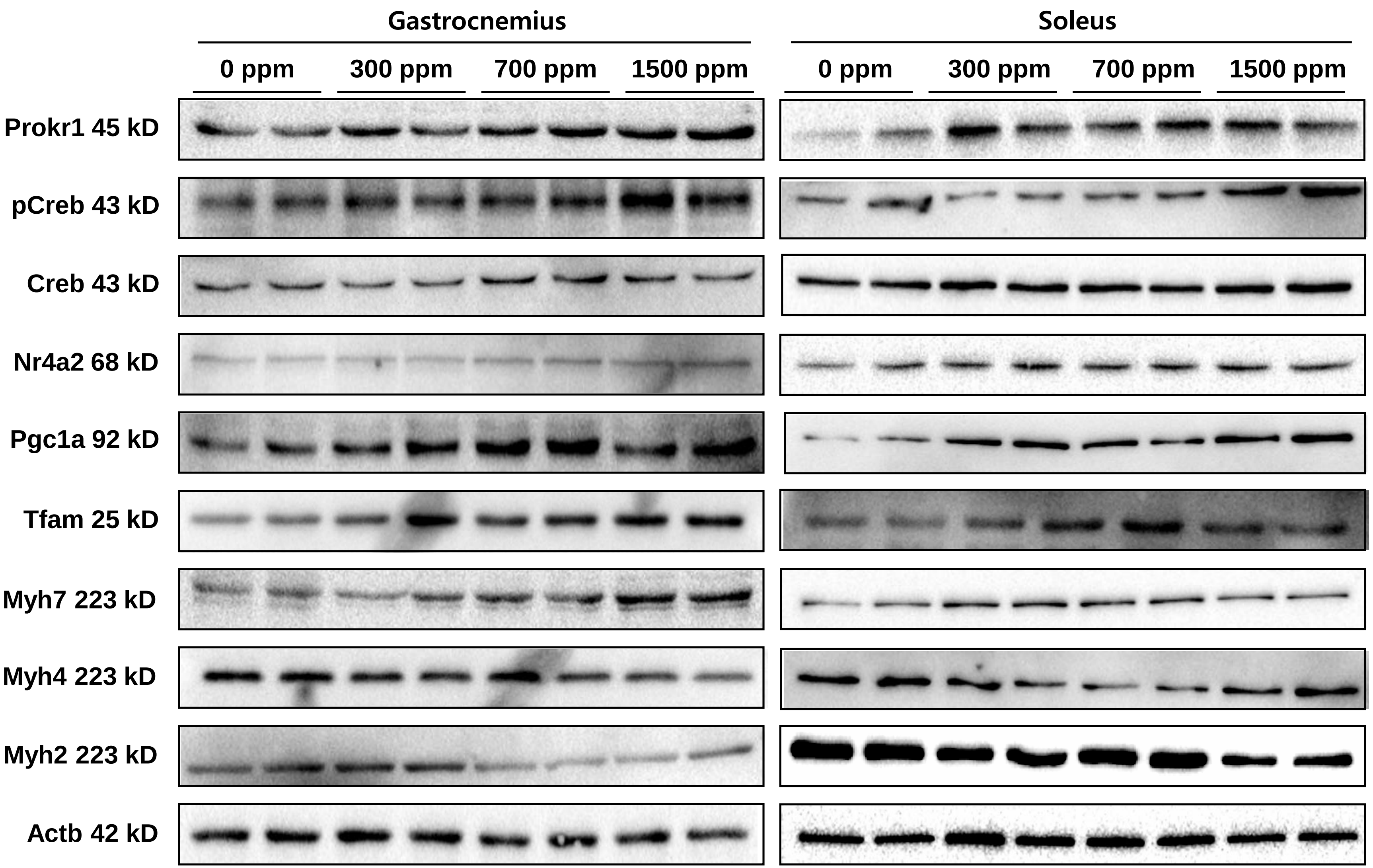

# Supplementary Fig. 49

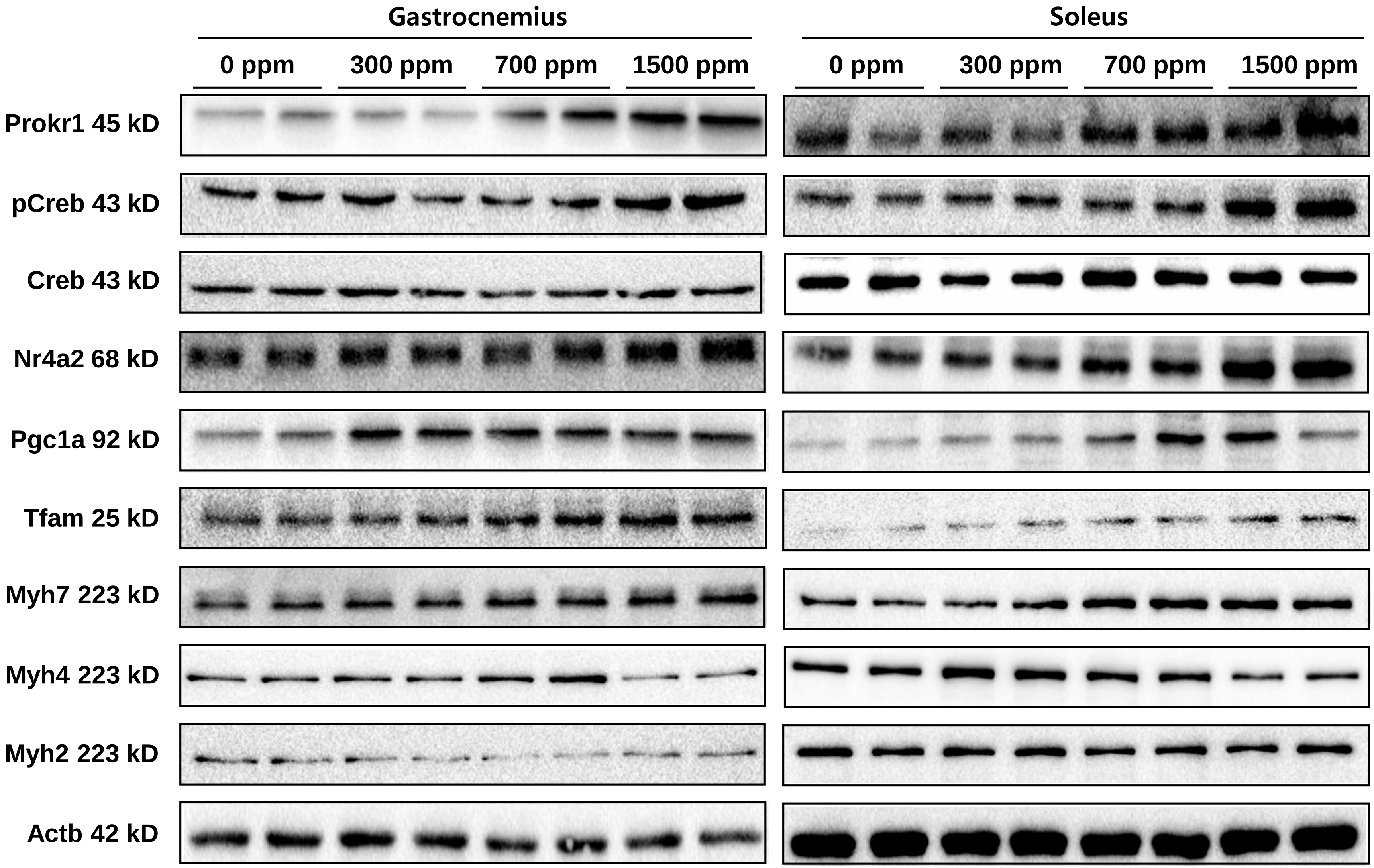

Supplement: Supplementary file 1 — Figure S1. Molecular docking of coxibs to human PROKR1. Figure S2. IP1 accumulation response in HEK293T cells. Figure S3. cAMP competition assay. Figure S4. Coxib‐induced oxidative muscle fibre specification. Figure S5. Western blotting of Prokr1 signalling molecules in C2C12 myotubes. Figure S6. Quantification of Myh4 and Myh2 in C2C12 myotubes. Figure S7. Immunocytochemistry of Myh4 and Myh2 in C2C12 myotubes. Figure S8. Total nuclei number and fusion index for C2C12 myotubes. Figure S9. Mitochondrial mass in C2C12 myotubes. Figure S10. Western blotting of PROKR1 signalling molecules in HSKMC myotubes. Figure S11. Quantification of MYH4 and MYH2 in HSKMC myotubes. Figure S12. Immunocytochemistry of MYH4 and MYH2 in HSKMC myotubes. Figure S13. Total nuclei number and fusion index for HSKMC myotubes. Figure S14. Mitochondrial mass in HSKMC myotubes. Figure S15. Attenuation of celecoxib‐induced oxidative muscle fibre specification in Prokr1 knock‐downed mouse myotubes. Figure S16. Body composition of neonatal mice. Figure S17. Relative muscle weights and muscle fibre counts of neonatal mice. Figure S18. Macroscopic examination of thigh muscle. Figure S19. H&E staining of neonate muscle tissues. Figure S20. Western blotting of Prokr1 signal molecules in male neonate muscle tissues. Figure S21. Western blotting of Prokr1 signal molecules in female neonate muscle tissues. Figure S22. Bodyweight changes of young adult mice. Figure S23. Food consumption of young adult mice. Figure S24. Body composition of young adult mice. Figure S25. Glucose tolerance of young adult mice. Figure S26. Insulin tolerance of young adult mice. Figure S27. Area under the glucose curve during ITT and basal (fasting) glucose levels of young adult mice. Figure S28. Respiratory exchange ratio of young adult mice. Figure S29. Energy expenditure of young adult mice. Figure S30. Organ weights of young adult mice. Figure S31. H&E staining of young adult mice muscle tissues. Figure S32. Mitochondria ma [file JCSM-16-e13704-s001.pdf]
